# Reverse Genetics Screen in Zebrafish Identifies a Role of miR-142a-3p in Vascular Development and Integrity

**DOI:** 10.1371/journal.pone.0052588

**Published:** 2012-12-21

**Authors:** Mukesh Kumar Lalwani, Meenakshi Sharma, Angom Ramcharan Singh, Rajendra Kumar Chauhan, Ashok Patowary, Naresh Singh, Vinod Scaria, Sridhar Sivasubbu

**Affiliations:** 1 Genomics and Molecular Medicine, CSIR-Institute of Genomics and Integrative Biology, Delhi, India; 2 G.N. Ramachandran Knowledge Center for Genome Informatics, CSIR-Institute of Genomics and Integrative Biology, Delhi, India; Medical College of Wisconsin, United States of America

## Abstract

MicroRNAs are a well-studied class of non-coding RNA and are known to regulate developmental processes in eukaryotes. Their role in key biological processes such as vasculature development has attracted interest. However, a comprehensive understanding of molecular regulation of angiogenesis and vascular integrity during development remains less explored. Here we identified miRNAs involved in the development and maintenance of vasculature in zebrafish embryos using a reverse genetics approach. Using a combination of bioinformatics predictions and literature based evidences we mined over 701 Human and 329 Zebrafish miRNAs to derive a list of 29 miRNAs targeting vascular specific genes in zebrafish. We shortlisted eight miRNAs and investigated their potential role in regulating vascular development in zebrafish transgenic model. In this screen we identified three miRNAs, namely miR-1, miR-144 and miR-142a-3p that have the potential to influence vascular development in zebrafish. We show that miR-142a-3p mediates vascular integrity and developmental angiogenesis *in vivo.* Overexpression of miR-142a-3p results in loss of vascular integrity, hemorrhage and vascular remodeling during zebrafish embryonic development, while loss of function of miR-142a-3p causes abnormal vascular remodeling. MiR-142a-3p functions in part by directly repressing *cdh5* (*VE-cadherin).* The vascular abnormalities that results from modulation of miR-142a-3p are reminiscent of *cdh5* perturbation in zebrafish embryos. We also demonstrate that the action of miR-142a on *cdh5* is potentially regulated by Lmo2, an important transcription factor, known for its role in vasculature development. The miR142a-3p mediated control of *cdh5* constitutes an additional layer of regulation for maintaining vascular integrity and developmental angiogenesis. These findings have implications in development, wound repair and tumor growth.

## Introduction

Endothelial cells (ECs), a major component of the blood vessels, provide a monolayer interface between the blood in the lumen and the surrounding tissues [1]. The ECs are multifunctional cells with selective permeability that facilitate the passage of metabolites and leukocytes into adjoining tissues [2]. These cells also enable the formation of new blood vessels from existing ones through a process known as angiogenesis [3]. The ECs perform these functions by regulating cell-cell adhesions between neighboring cells [3], [4]. Endothelial cell injury or dysfunction has been associated with a variety of pathological and disease conditions ranging from atherosclerosis, inflammation, tumor metastasis, hypertension and stroke [5].

Vascular Endothelial Cadherin (VE-cad), an endothelial-specific transmembrane component of the adheren junction complex, has emerged as an important regulator of endothelial cell-cell adhesion with well-defined roles in angiogenesis, vascular remodeling and permeability [2]. VE-cad is expressed in all ECs of the vasculature and promotes cell-cell adhesion through dimerization of its extracellular amino-terminal repeats [6]. The cytoplasmic domain of VE-cad facilitates cell signaling through interactions with members of the armadillo repeat family of proteins including β-catenin, plakoglobin and p120 [6], [7]. VE-cad mediated ECs permeability is known to be regulated largely by phosphorylation of tyrosine residues in VE-cad, β-catenin and p120 or through vascular endothelial growth factor (VEGF) mediated clathrin dependent internalization of VE-cad [6], [8].

Studies involving inactivation of VE-cad in animal models have established the importance of this protein in endothelial cell biology. VE-cad deficient mice die mid-gestation with major defects in vascular development [9]. Investigations in VE-cad deficient mice embryos have revealed the proper development of primitive vascular plexus; however further development of the vessels was hampered leading to severe defects in extraembryonic vasculature [10]. In model organisms such as the zebrafish, downregulation of VE-cad did not affect vasculogenesis, however impaired vascular connections and inhibition of vascular sprouting activity was observed [11]. VE-cad downregulation in zebrafish has also been shown to inhibit tumor neovascularization without affecting the development of intersegmental and subintestinal vessels [12]. Collectively these studies have proposed that VE-cad is not essential for primary vasculogenesis but is indispensable for the subsequently remodeling and morphogenesis of vessels. Further studies have revealed additional functions of VE-cad during early zebrafish cardiac development [13].

MicroRNAs (miRNAs) a class of 17–25 nucleotide, genome encoded non-coding RNAs have emerged as key regulators of normal physiological processes of vertebrate development such as apoptosis, epithelial to mesenchymal transition, hematopoiesis and vasculogenesis, and have been extensively studied in human and model organisms [14]–[16]. MiRNAs are known to regulate expression of protein coding genes at posttranscriptional level either through repression of protein translation or degradation of target mRNAs [17]. Mice homozygous for a hypomorphic allele of Dicer, an enzyme essential for the biogenesis of most miRNAs, develop gross abnormalities of blood vessels in the embryo and in the yolk sac [18]. The vascular specific miRNA, miR-126 has been shown to play a role in endothelial tube organization and maintenance of blood vessel integrity, both *in vitro* and *in vivo* [19]–[21]. Zebrafish *meunier* mutant has diminished miR-144/451 expression with retarded erythrocyte maturation, showed partial rescue of mutant phenotype solely by miRNA overexpression [22]. The erythroid specific miR-144 has been shown to negatively regulate embryonic α*-hemoglobin* (α*-E1*), by targeting the 3′-UTR of *Kruppel-like factor D* (*klfd*) gene physiologically [23]. These studies suggest potential functions of miRNAs during embryonic development and organogenesis [24]. Deciphering the role of miRNAs during vasculature development would enable better understanding of the vascular biology in normal and pathological conditions.

In this study we investigated the role of miRNAs involved in regulating vascular development and maintenance. Using a combination of bioinformatics predictions and literature based evidences we mined over 701 Human and 329 Zebrafish miRNAs. We derived a list of miRNAs targeting putative vascular specific genes in zebrafish. We shortlisted eight miRNAs and investigated their potential role in regulating vascular development using zebrafish as a model organism. Of these, three miRNAs, miR -144, miR-1 and miR-142a-3p revealed specific non-overlapping phenotypes affecting vascular development. Detailed investigation of miR-142a-3p revealed that overexpression of miR-142a-3p resulted in a cerebral/trunk hemorrhage along with abnormal cranial vasculature remodeling in developing zebrafish embryos. Using an antisense morpholino designed against miR-142a-3p, we successfully rescued miR- 142a-3p overexpression phenotype and demonstrate the specificity of the miRNA-induced phenotype. We also noticed that loss of function of miR-142a-3p resulted in vascular remodeling of intersegmental vessels (Se) in developing zebrafish embryos. We show that miR-142a-3p interacts with *VE-cadherin* (*cdh5)* gene in zebrafish by targeting the binding sites in the 3′UTR of *cdh5* mRNA. Overexpression of miR-142a-3p in developing zebrafish embryos resulted in significant reduction of endogenous *cdh5* gene at transcript as well as protein level. In parallel, down regulation of the endogenous miR-142a-3p resulted in increased expression of endogenous *cdh5* gene, suggesting a direct interaction between miR-142a-3p and *cdh5* gene to regulate vascular integrity and remodeling in developing zebrafish embryos. Furthermore we show that the action of miR-142a on *cdh5* is in part regulated by Lmo2, an important transcription factor, known for its role in vasculature development.

## Results

### Selection of miRNAs Involved in Vasculature Development

**Figure 1:**
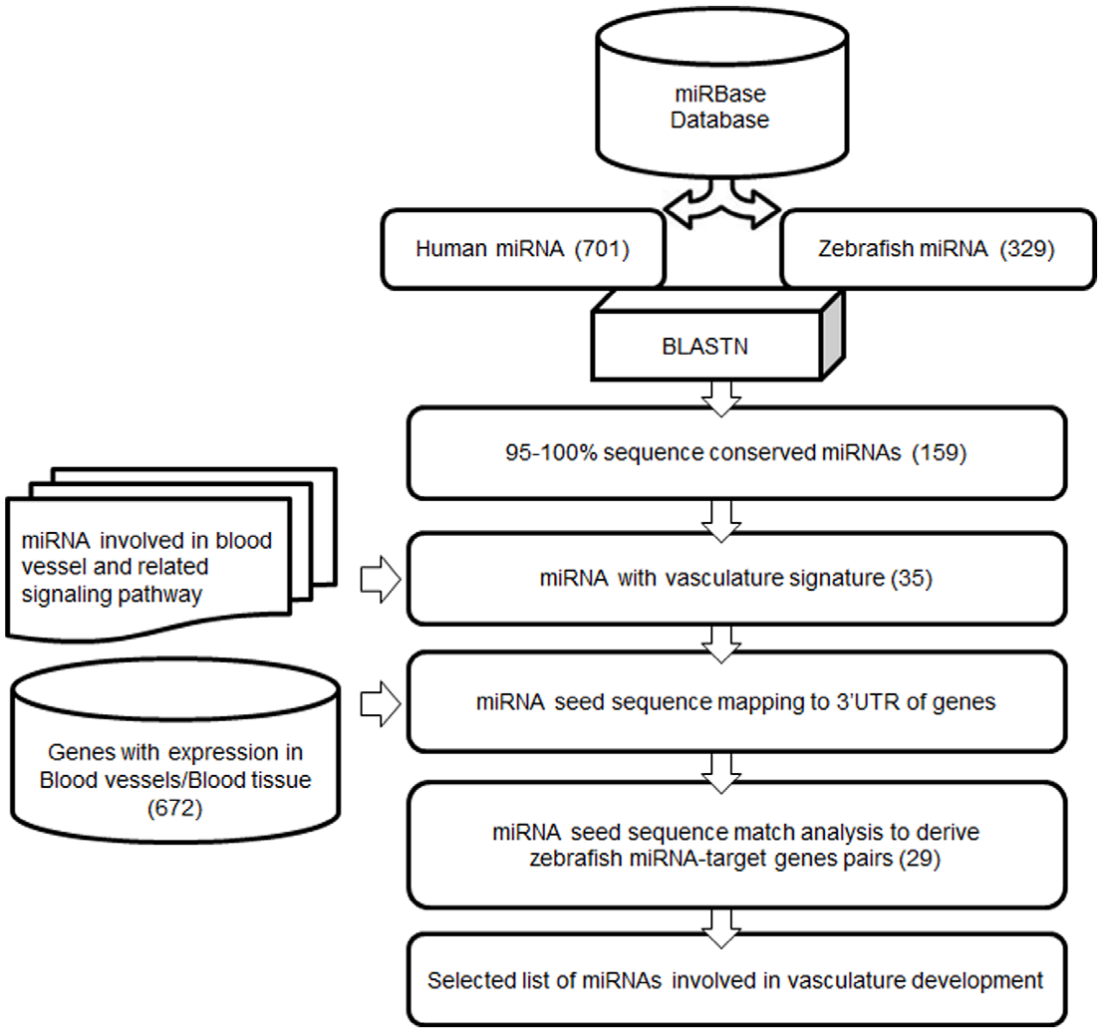
*In silico* pipeline for selection of miRNAs involved in vasculature development.

A total of 701 Human and 329 Zebrafish mature miRNA sequences obtained from miRBase were investigated using BLASTN for sequence conservation [25], [26]. We identified 159 miRNAs with 95–100% sequence conservation between zebrafish and human for our study (Figure 1, Table S1). The list was further refined by retaining only those miRNA that were either directly or indirectly indicated from literature or meta-studies to be associated with blood vessel development process. The above enrichment generated a list of 35 miRNAs that had putative function in vascular development and were labeled as miRNAs with “vasculature signature” (Figure 1, Table S2). This prioritized list of 35 miRNAs was used for downstream miRNAs-gene target pair prediction. Separately we generated a dataset of 672 zebrafish genes, from the Zebrafish Information Network (www.zfin.org), with putative expression in blood and blood vessel tissue (Figure 1, Table S3) [27]. These 672 zebrafish genes were used for performing miRNA seed sequence match analysis to derive putative zebrafish miRNA-gene target pairs (Table S4) [28]. Based on the analysis described above, a list of 29 miRNAs targeting zebrafish genes were generated (Figure 1, Table S4). We performed a ‘gain of function’ reverse genetic screen on eight miRNAs, to investigate their potential role in zebrafish vascular development.

### Overexpression of Selected miRNAs in Zebrafish Embryos Induces Specific Vascular Phenotypes

**Figure 2:**
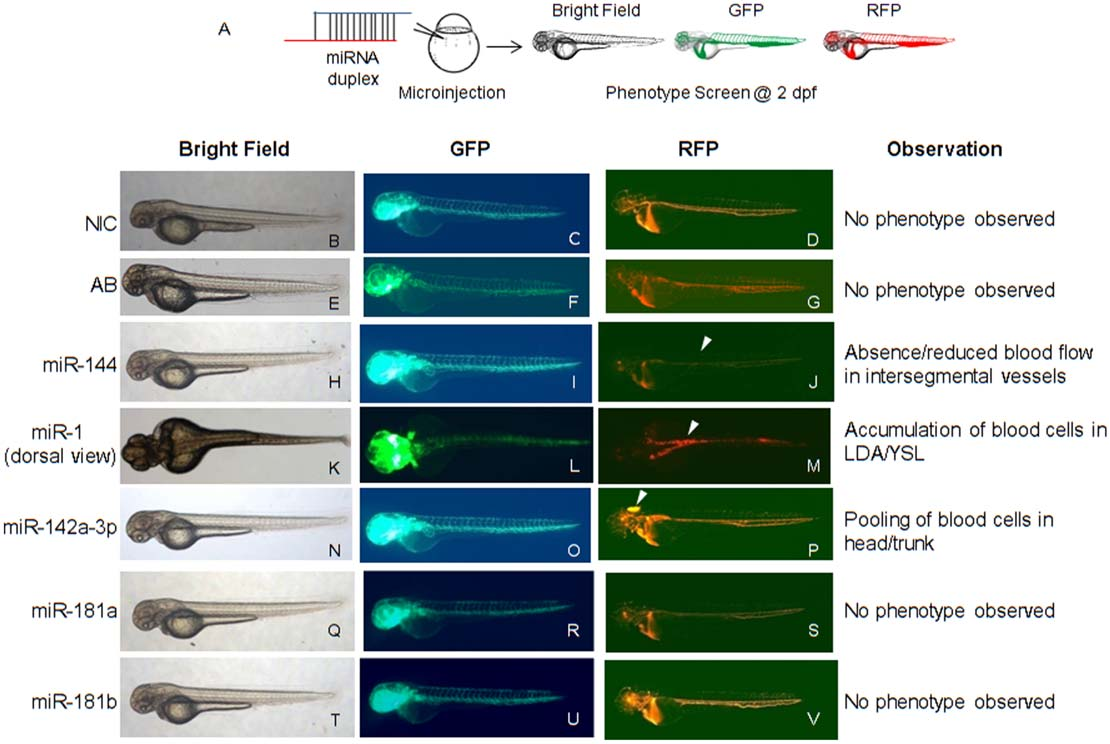
Overexpression of miRNAs in zebrafish embryos induces specific vascular phenotypes. A - Schematic of the experimental approach. MiRNA duplexes were ectopically overexpressed in Tg*(fli1:EGFP, gata1a: dsRed)* zebrafish embryos through microinjection at 1–2 cell stage. The injected embryos were scored at 2 dpf under microscope for visual observation of phenotype under bright field, GFP and RFP filters. Representative images of miRNA-injected Tg*(fli1:EGFP, gata1a: dsRed)* zebrafish embryos are displayed. B,C,D - Non-injected control (NIC) zebrafish embryos with normal vascular development. E,F,G - Zebrafish embryos injected with miRNA annealing buffer display normal vascular development. H,I,J - Zebrafish embryos injected with miR-144 display reduced or absence of blood in intersegmental vessels. K,L,M - Zebrafish embryos injected with miR-1 display accumulation of blood cells in LDA/YSL. N,O,P - Zebrafish embryos injected with miR-142a-3p display pooling of blood cells in head/trunk region. Q,R,S and T,U,V - Zebrafish embryos injected with miR-181a and miR-181b respectively display no visible phenotype. Zebrafish embryos injected with miR-221, miR-222 and miR-451 display no observable phenotype (figure not shown). Arrowheads indicate the site of vascular defects. The embryos were imaged at 2.5× magnification.

Synthetic double stranded miRNA duplexes overexpressed ectopically by microinjection at 1–2 cell stage of zebrafish embryos are expected to be processed inside the cells and mimic endogenous mature miRNA [22], [24], [29]. We have used a similar strategy to characterize the *in vivo* function of eight prioritized miRNAs (miR-144, miR-1, miR-142a-3p, miR-181a, miR-181b, miR-221, miR-222 and miR-451), and investigated their potential effect on vascular development using double transgenic Tg*(fli1:EGFP, gata1a: dsRed)* zebrafish embryos (Figure 2). Microinjection of miRNA duplex annealing buffer in Tg*(fli1:EGFP, gata1a: dsRed)* zebrafish embryos resulted in no observable phenotype till two days post fertilization (dpf). Microinjection of duplex miR-144 (20 µM) resulted in a reduction/absence of blood flow in trunk intersegmental vessel (Se) at 2 dpf in approximately 68% of injected animals (n = 63/92). Microinjection of duplex miR-1 (10 µM) in zebrafish embryos showed accumulation of blood cells in lateral dorsal aorta (LDA) and yolk syncitial layer (YSL) with disrupted blood flow at 2 dpf in approximately 60% of injected animals (n = 56/93). Microinjection of duplex miR-142a-3p (10 µM) induced cerebral/trunk hemorrhage and pericardial edema in approximately 55% of injected animals (n = 113/206). Overexpression of miR-181a, miR-181b, miR-221, miR-222 and miR-451 (10 µM) resulted in no observable phenotype in zebrafish embryos at 2 dpf. Thus, delivery of 10–20 µM duplex miRNA in developing zebrafish embryos does not generate non-specific effects. Ectopic overexpression of miRNAs in zebrafish embryos resulted in a variety of non-overlapping developmental phenotypes without any accompanying gross morphological side effects and three of the eight tested miRNAs showed specific vasculature phenotypes in zebrafish.

### Overexpression of miR-142a-3p Induces Cerebral/trunk Hemorrhage and Vascular Remodeling in Zebrafish Embryos

**Figure 3:**
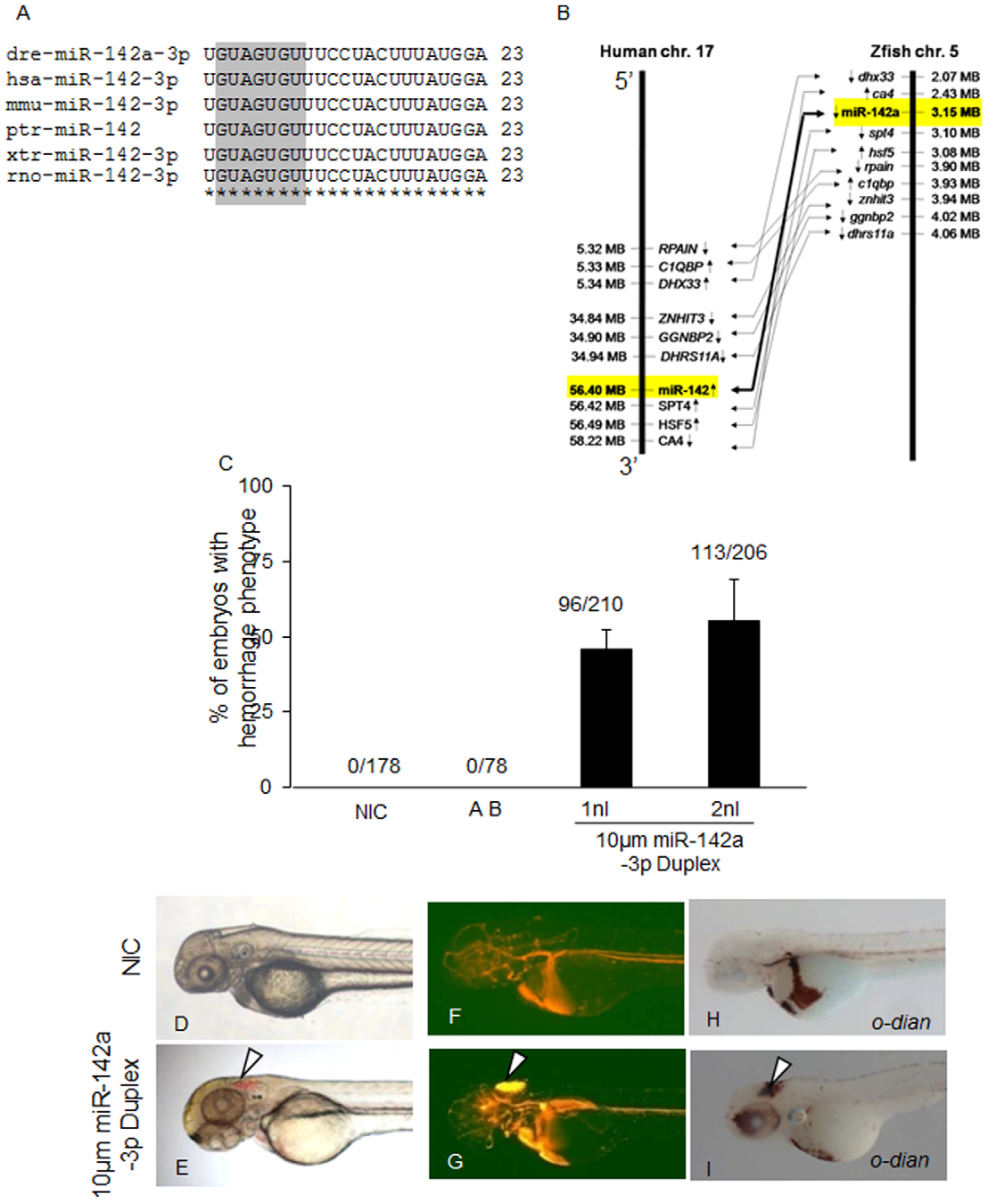
Overexpression of miR-142a-3p in zebrafish embryos induces hemorrhage and vascular remodeling. A - Sequence alignment of mature miR-142a-3p from different species is highlighted with seed sequence in gray color. B – Synteny analysis of human miR-142 on chromosome 17 with zebrafish miR-142a on chromosome 5. Arrow in genes indicates strand information on chromosome. C - Bar graph showing cerebral hemorrhage phenotype in miR-142a-3p duplex injected Tg*(fli1:EGFP, gata1a: dsRed)* zebrafish embryos at 2 dpf. Data is represented as mean percentage ± SD (standard deviation) collected over 3 independent experiments and n is number embryos analyzed. D - I - Representative image of cerebral hemorrhage phenotype in Tg*(fli1:EGFP, gata1a: dsRed)* zebrafish embryos at 2 dpf. D,F,H - Non-injected control embryos (NIC) and E,G,I - miR-142a-3p injected embryos. H,I - Embryos stained with *o-dianisidine*. D–I - 2.5× magnification.

The miR-142a-3p has been identified in different organisms with 100% similarity at nucleotide sequence level (Figure 3A). Also the miR-142a genomic loci share syntenic relationship between human and zebrafish (Figure 3B). Ectopic overexpression of miR-142a-3p duplex in Tg*(fli1:EGFP, gata1a: dsRed)* zebrafish embryos resulted in hemorrhage in head and trunk region, and cranial vessel remodeling in 45–55% of the injected embryos at 2 dpf (Figure 3C *).* Amongst the embryos with hemorrhages, majority displayed cranial hemorrhage and a minor proportion (5–8%) had hemorrhage in the trunk region. The cranial and trunk hemorrhage phenotypes were evident under a microscope with bright field and Red Fluorescent Protein (RFP) filter (Figure 3D–3G). The hemorrhage phenotype was also confirmed by *o-dianisidine* staining of erythrocytes (Figure 3H, 3I).

**Figure 4:**
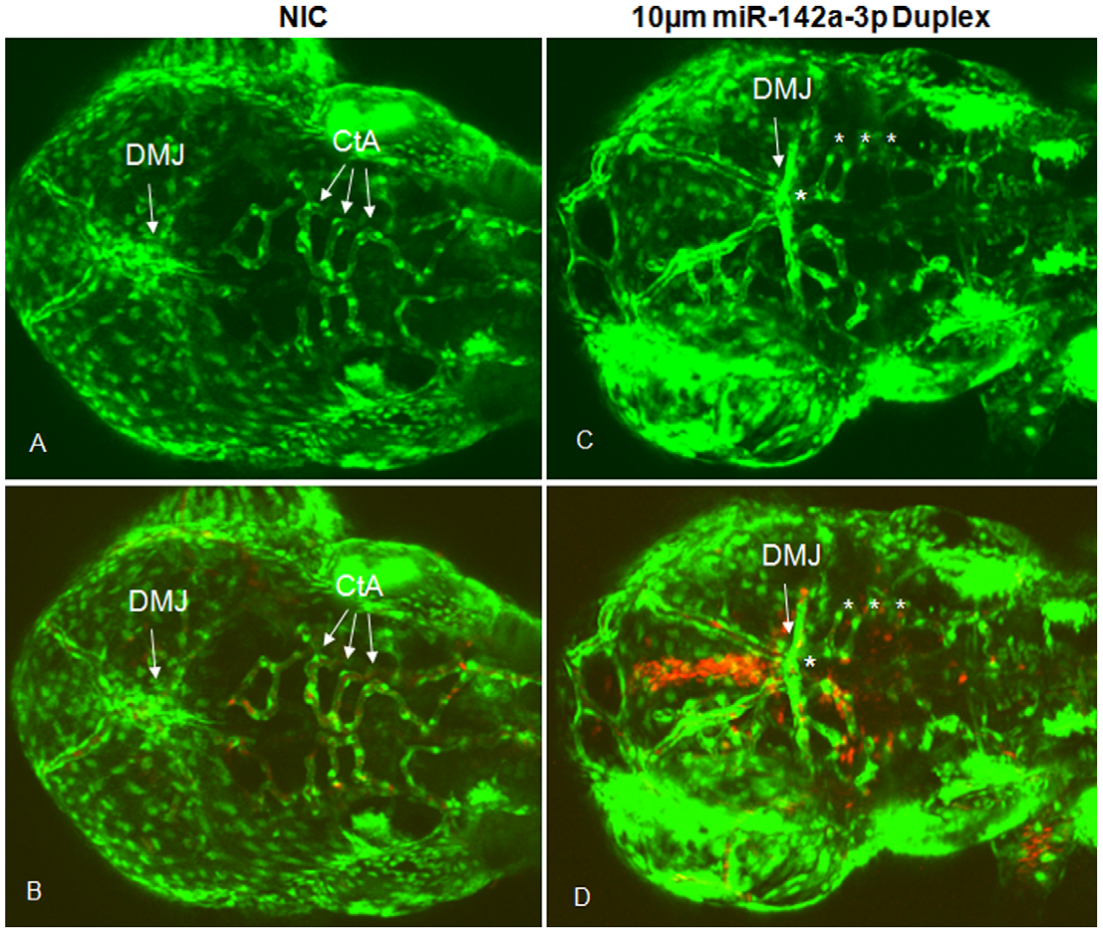
Overexpression of miR-142a-3p in zebrafish embryos induces vascular defects. A – D - Confocal images (GFP and GFP/RFP merged) of 10 µM miR-142a-3p duplex injected Tg*(fli1:EGFP, gata1a: dsRed)* embryos at 2 dpf (Dorsal View of head) depicting dorsal midbrain junction (DMJ) and central arteries (CtA) (10× magnification). A, B - non-injected control embryos and C, D - 10 µM miR-142a-3p duplex injected embryos. A, C – GFP, B, D – GFP/RFP merged. Asterisk sign indicate the site of vascular defect.

Analysis of the organization of vasculature in the head region of miR-142a-3p duplex injected embryos with confocal microscope revealed multiple vascular remodeling defects in the miR-142a-3p duplex injected embryos (Figure 4A–4D). The defects were noticed in dorsal midbrain junctions (DMJ) and central arteries (CtA) that extended from the basal communicating artery (BCA) to irrigate forebrain and midbrain [30]. In few embryos, the hemorrhage phenotype was also accompanied by pericardial edema and slow blood flow (data not shown).

To rule out the possibility of non-specific effect generated by the action of any one of single strand of the miRNA we also conducted separate microinjections of the sense and antisense strand of miR-142-3p. We did not observe any distinct phenotype in zebrafish embryos injected with the single strand of the miRNA (data not shown). The specificity of the miR-142a-3p duplex induced hemorrhage phenotype was tested by injecting an antisense morpholino targeting the mature form of miR-142a-3p. The optimal dose of the morpholino was determined by injecting zebrafish embryos with 10–500 µM of miR-142a-3p MO (Figure S1A). We observed that in embryos receiving 10 µM miR-142a-3p duplex alone, 47% (64/134) of embryos display the hemorrhage phenotype. However upon co-injection of 10 µM miR-142a-3p duplex and 100 µM miR-142a-3p MO, the hemorrhage phenotype was rescued in approximately 50% of embryos (35/129) (Figure S2).

### Loss of miR-142a-3p Leads to Intersegmental Vessel (Se) Remodeling

**Figure 5:**
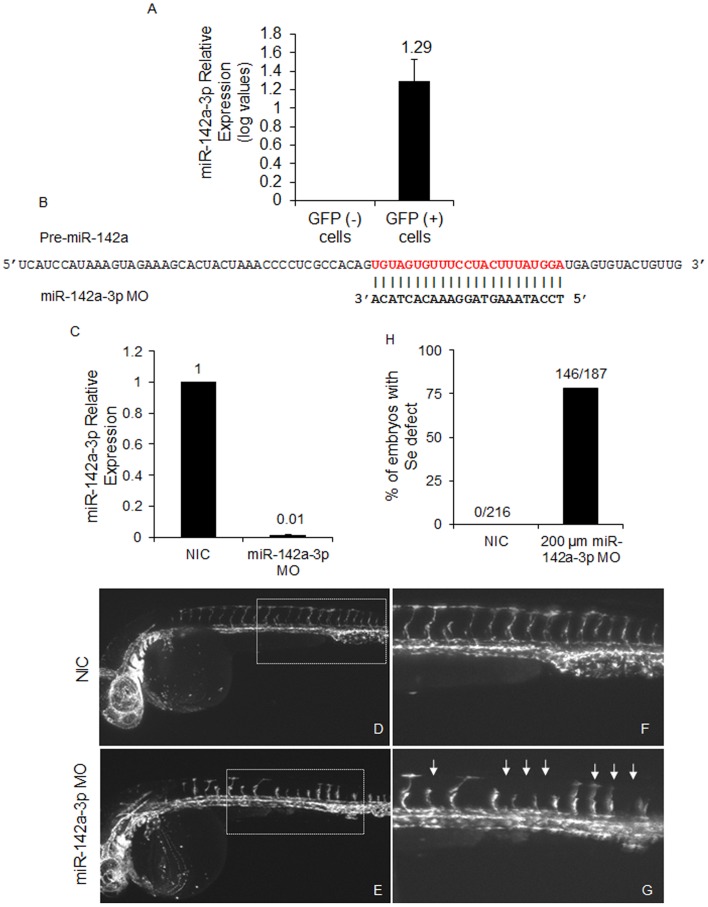
Loss of miR-142a-3p leads to intersegmental vessel remodeling. A –Relative quantification of miR-142a-3p in GFP positive (+) and GFP negative (–) cells of 2 dpf Tg*(fli1:EGFP, gata1a: dsRed)* zebrafish embryo by real-time PCR. Total RNA was isolated from 2 dpf FAC sorted GFP (–) and GFP (+) cells of 2 dpf Tg*(fli1:EGFP, gata1a: dsRed)* zebrafish embryos. 200 ng of total RNA was used for preparing cDNA using QuantiMir kit, SBI, USA and diluted by 1∶1 for RT-PCR assay. The assay has been performed using ΔΔCT method (ΔΔCT = (CT miRNA - CT reference RNA) - (CT calibrator - CT reference RNA) as described previously [45]. U6 and beta-actin was used as calibrator and reference control respectively. Data collected from 3 independent experiments is represented as mean fold change ± SD. B – Schematic alignment of miR-142a-3p MO with pre-miR-142a. Mature miR-142a-3p sequence is in red color. C - Relative quantification of mature miR-142a-3p in non-injected control (NIC) and embryos injected with 200 µM miR-142a-3p MOs at 2 dpf. The assay was performed as described by manufacturer (QuantiMir kit, SBI, USA). The relative expression of miR-142a-3p was normalized to miR-26a. Data collected from 3 independent experiments is represented as mean fold change ± SD. D - G - Representative image of 28–30 hpf Tg*(fli1:EGFP, gata1a: dsRed)* zebrafish embryos. Inset displaying intersegmental vessels from non-injected control and 200 µM miR-142a-3p morpholino injected embryos. Images are arranged in a lateral view and inset displaying 11–15 intersegmental vessels from the trunk region. The images are taken at 5× and 10× magnifications. Arrowheads indicate regions with vascular defects. H - Bar graph showing embryos with inter-segmental vessel defects in non-injected control (NIC) and 200 µM miR-142a-3p morpholino injected embryos at 28–30 hpf.

Previously we noticed that ectopic overexpression of miR-142a-3p duplex in zebrafish embryos caused hemorrhage in head and trunk region and abnormal cranial vessel remodeling. We argued that if the hemorrhage and vessel remodeling is specifically caused by over expression of miR-142a-3p then the down regulation of the endogenous levels of miR-142a-3p should also cause a phenotype in the same tissue i.e. blood vessel. Therefore, we analyzed the effects of blocking the mature form of endogenous miR-142a-3p using a morpholino (MO) antisense oligonucleotide (miR-142a-3p MO). First, in order to check the endogenous expression of miR-142a-3p in blood vessel, we examined the relative expression levels of miR-142a-3p in FAC sorted endothelial cells expressing GFP under the promoter of *fli1* transcription factor using 2dpf Tg*(fli1:EGFP, gata1a: dsRed)* developing embryo. We observed that miR-142a-3p was ∼ 22.5 fold over expressed in GFP positive cells compared to GFP negative cells suggesting its enriched expression in endothelial cells (Figure 5A). Microinjection of miR-142a-3p MO at concentration of 200 µM caused specific vascular phenotype (Figure S1B – S1G). The miR-142a-3p MO injected animals at 200 µM has displayed reduced expression level of endogenous miR-142a-3p to 0.01±0.007 (Figure 5C) In the miR-142a-3p knockdown embryos (28–30 hpf), the primary vasculature appeared normal, however the intersegmental vessels (Se) displayed defects and abnormal remodeling in a significant proportion (≥78%) of the injected embryos (Figure 5D –5H, Figure S1B – S1G). No visible off target effects were noticed in the injected embryos.

Optical sectioning microscope analysis revealed that intersegmental vessels (Se) in non-injected control embryos sprout and extend dorsally, making connections from dorsal aorta (DA) to dorsal longitudinal anastomotic vessel (DLAV). However in miR-142a-3p knockdown embryos, the intersegmental vessels sprout and extend dorsally but either fails to make contacts with the DLAV (data not shown) or are irregularly patterned (Figure 5D –5G and S1B – S1G). These data demonstrate that morpholino mediated knockdown of endogenous miR-142a-3p in zebrafish embryos do not affect primary vasculogenesis however angiogenesis and intersegmental vessels (Se) remodeling were affected.

### Potential Targets of miR-142a-3p

**Figure 6:**
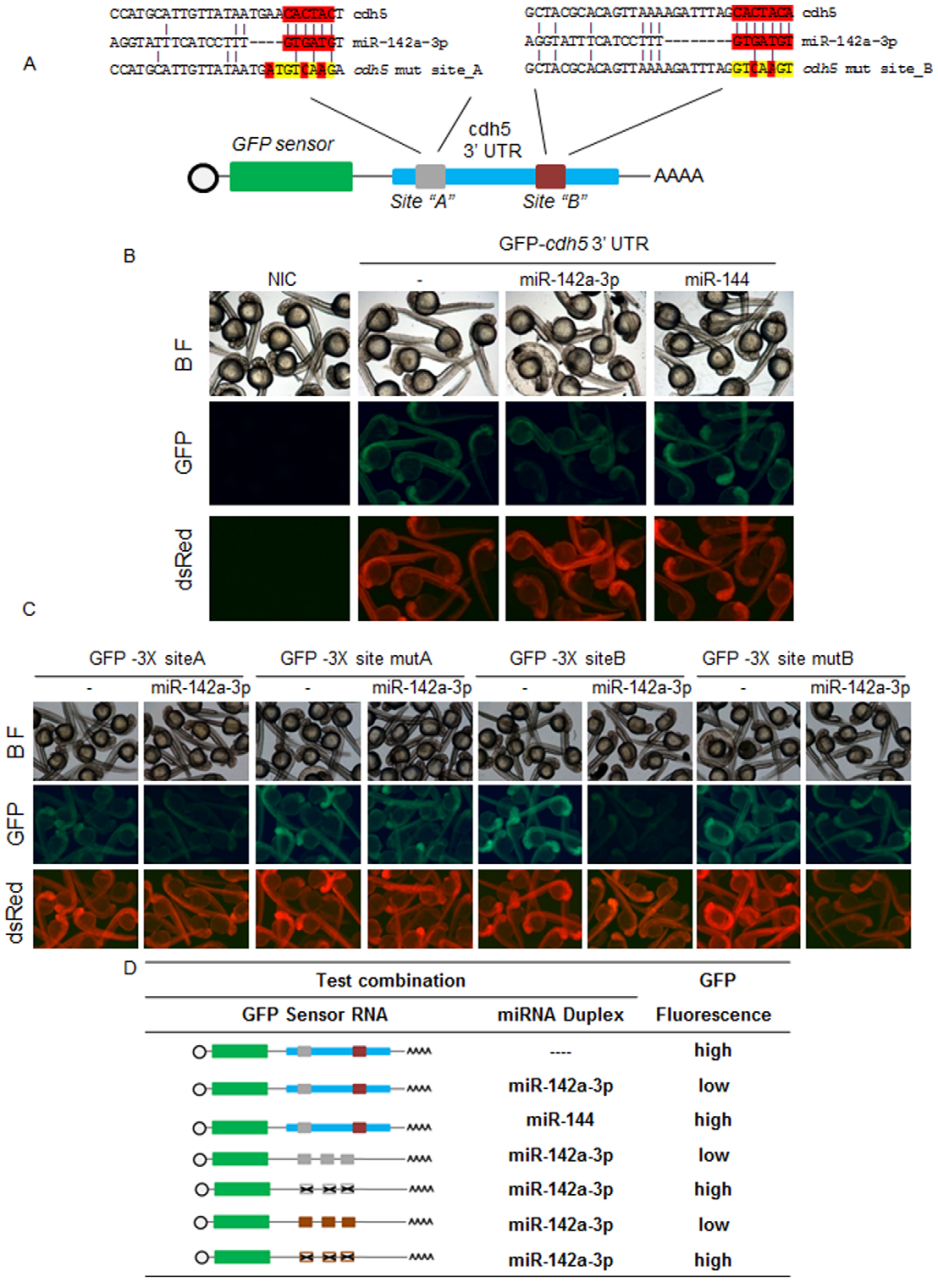
In zebrafish, miR-142a-3p regulates cdh5 via its two predicted binding sites. A - Schematic of GFP-*cdh5* 3′UTR transcript containing two miR-142a-3p predicted binding site (site “A”, grey; site “B”, brown). Predicted miRNA seed sequences binding sites are enlarged and boxed in red. Selected nucleotides were mutated in the seed region for destroying miR-142a-3p binding site is displayed in yellow color. B and C - Silencing effect of miR-142a-3p on the series of sensor mRNA encoding GFP fused to various test 3′UTRs (as tabulated in panel D). B - Co-injection of the miR-142a-3p and GFP-*cdh5* 3′UTR mRNA leads to suppression of GFP expression in wildtype zebrafish embryos. C – Co-injection of miR-142a-3p with various test combinations of GFP sensor fused to the miR-142a-3p seed sequence binding sites, engineered in triplicates. Strong miR-142a-3p induced suppression of GFP expression was observed with both the predicted binding sites (GFP-3′UTR*3X* site A and GFP-3′UTR*3X* site B)^.^ However, suppression was relieved on mutating the binding sites (GFP-3′UTR*3X mut* site A and GFP-3′UTR*3X mut* site B). Bright-field image of embryos are shown in the upper row of each panel. DsRed mRNA was co-injected with the test transcripts as a control and is shown in the lower row of each panel. In the middle row of each panel various GFP-3′UTR sensor RNA and miRNA combinations were tested as labeled. Expressions of the reporters were analyzed at 26 to 28 hours post fertilization. Group images of embryos that were co-injected of various GFP- 3′UTR mRNA and miR-142a-3p are compared with embryos receiving respective GFP-3′ UTR sensor only. Clutch of representative embryos (7–9 embryos in each image) were photographed together in a single image to ensure valid comparison of relative green fluorescent intensity between two groups. The images are taken at 2.5× magnification. D - Various combinations of GFP sensor and miRNA were tested and scored using a comparative scale for GFP intensity and are represented either as high or low. The colour schemes of the cdh5 3′ UTR variants follow those mentioned in Figure 6A. The black “X” indicates a mutated miR-142a-3p site. The bioactivity of GFP-Sensor with 4X miR-142a-3p perfect complimentary target (PT) sites and miRNA duplex is shown in Supplementary Figure S5.

To investigate the potential target of miR-142a-3p, we focused our analysis on the 672 genes that have putative expression in blood and blood vessel tissue in zebrafish (see previous section on selection of miRNAs). We searched for complementary seed sequence binding site of miR-142a-3p in the 3′UTR of 672 genes. We observed that 52 genes contain a putative binding site for miR-142a-3p (Table S4). Our attention was drawn to two genes from this list, namely *vegfab* (Entrez gene ID: 558154) and *cdh5* (Entrez gene ID: 445471) as downregulation of these genes were previously known to produce a cranial hemorrhage phenotype in zebrafish [11], [31]. *In silico* miR-142a-3p “seed sequence” search revealed the presence of two potential binding sites in the 3′ UTR of *vegfab* and *cdh5* gene (Figure 6A and S3A).Therefore, we have tested the 3′UTRs of *vegfab* and *cdh5* to check if they harbored bonafied binding site of miR-142a-3p using a GFP sensor assay [22], [24]. No suppression of GFP expression was observed in zebrafish embryos in the sensor assay when 3′UTR of *vegfab* gene was co-injected with the miR-142a-3p duplex (Figure S3B). However when tested with the 3′ UTR of *cdh5,* significant suppression of GFP expression was noticed (Figure 6A and 6B). The specificity of the binding of the 3′ UTR of *cdh5* gene with miR-142a-3p duplex was investigated by microinjecting GFP- *cdh5*3’UTR mRNA, in the presence and absence of miR-142a-3p duplex and a sequence unrelated miR-144 duplex. Zebrafish embryos sequentially injected with GFP- *cdh5*3’UTR mRNA and miR-142a-3p duplex showed suppression of GFP expression, while miR-144 duplex had no effect on the GFP expression (Figure 6B). This suggests that the 3′UTR of *cdh5* specifically interact with miR-142a-3p.

### In Zebrafish, miR-142a-3p Regulates *cdh5*

*In silico* miR-142a-3p “seed sequence” search revealed the presence of two potential binding sites in the 3′ UTR of *cdh5* gene (Figure 6A). We wanted to investigate which of the two predicted binding sites in the 3′UTR of *cdh5* gene was targeted by miR-142a-3p. Therefore, we tested the binding efficiency of miR-142a-3p to its two predicted binding sites using the GFP sensor assay (Figure 6C) [22, [24]. Zebrafish embryos were coinjected with miR-142a-3p duplex and mRNA encoding GFP fused to the individual miR-142a-3p binding sites in triplicate (GFP-3′UTR*3X* site A and GFP-3′UTR*3X* site B). Strong miR-142a-3p induced suppression of GFP expression was observed with both the predicted binding sites present in the 3′UTR of *cdh5* (Figure 6C). The miR-142a-3p mediated repression on the 3′UTR of *cdh5* was abolished when both seed sequences were mutated (GFP-3′UTR*3X mut* siteA and GFP-3′UTR*3X mut* siteB ) to avoid miRNA seed base pairing (Figure 6C). Collectively, these data suggest that miR-142a-3p targets the 3′UTR of *cdh5* gene via the two predicted recognition sites (Figure 6D).

**Figure 7:**
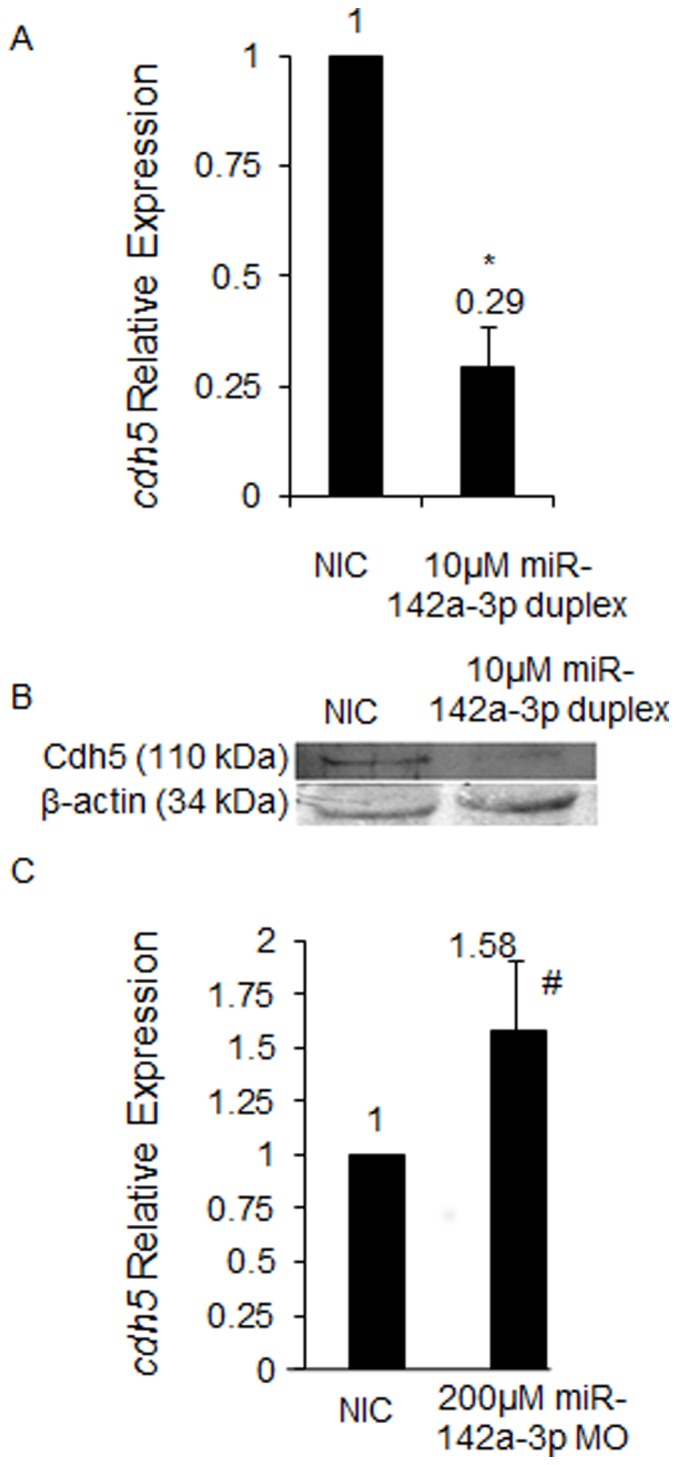
In zebrafish, miR-142a-3p regulates *cdh5.* A - *cdh5* relative expression quantified by QRT–PCR upon overexpression of miR-142a-3p. Total RNA was isolated from 2 dpf non-injected control (NIC) and 10 µM miR-142a-3p duplex injected Tg*(fli1:EGFP, gata1a: dsRed)* zebrafish embryos. 2 ug of total RNA was used for preparing cDNA. Beta-actin was used as an internal control. Data collected from 4 independent experiments is represented as mean fold change ± SD. Asterisk (*) indicates p value of 0.001 as determined by 2-tailed t-test. B - Western Blot analysis for Cdh5 protein in zebrafish embryos using previously tested antibody directed against human VE-cad (110 kDa) [13] in non-injected control (NIC) and 10 µM miR-142a-3p duplex injected 2 dpf zebrafish embryos. Beta-actin was used as a loading control. C - *cdh5* relative expression quantified by QRT–PCR upon downregulation of miR-142a-3p. Total RNA was isolated from 3 dpf non-injected control (NIC) and 200 µM miR-142a-3p MO injected Tg*(fli1:EGFP, gata1a: dsRed)* zebrafish embryos. 2 ug of total RNA was used for preparing cDNA. Beta-actin was used as an internal control. Data collected from 4 independent experiments is represented as mean fold change ± SD. Hash (#) indicates p value of 0.03 as determined by 2-tailed t-test.

We also analyzed the endogenous levels of *cdh5* transcript in zebrafish embryos upon overexpression and downregulation of miR-142a-3p to investigate the direct modulation of *cdh5* expression by miR-142a-3p (Figure 7). Overexpression of miR-142a-3p resulted in the reduction of *cdh5* transcript level by approximately 3.4 fold (0.29±0.15) compared to control embryos (Figure 7A). The western blot analysis of Cdh5 (VE-cadherin) protein showed decrease in endogenous protein level in miR-142a-3p injected zebrafish embryos (Figure 7B) [13] [13]. Next we checked the levels of endogenous *cdh5* transcript upon downregulation of endogenous miR-142a-3p in zebrafish embryos. We found that expression of *cdh5* increased by approximately 1.58±0.32 fold upon morpholino-mediated downregulation of endogenous miR-142a-3p (Figure 7C). In summary we show that miR-142a-3p directly binds with the two predicted two target sites in the 3′UTR of *cdh5* gene and modulates the expression of *cdh5* transcript and protein in zebrafish embryos.

### Cdh5 and miR-142a-3p are Regulated by the Transcription Factor Lmo2 in Zebrafish

We show that miR-142a-3p could regulate *cdh5* gene post-transcriptionally and modulate the levels of *cdh5* transcript and protein in zebrafish embryos. We were intrigued with the possible biological reasons on why miR-142a-3p should regulate *cdh5* gene. Studies conducted on human cancers by other groups from have indicated that LMO2, a transcription factor expressed in blood and vasculature acts as a transcriptional suppressor of miR-142 [32]. LMO2 is also a known to bind to the promoter of *CDH5* gene and regulate its transcription [33]. So we hypothesized that, if *lmo2* gene regulates expression of *cdh5*/miR-142 transcriptionally and miR-142a-3p regulates *cdh5* gene post-transcriptionally, then *lmo2*, *cdh5* and miR-142a-3p may be part of an interacting network that could regulate angiogenesis and vasculature remodeling in zebrafish embryos.

**Figure 8:**
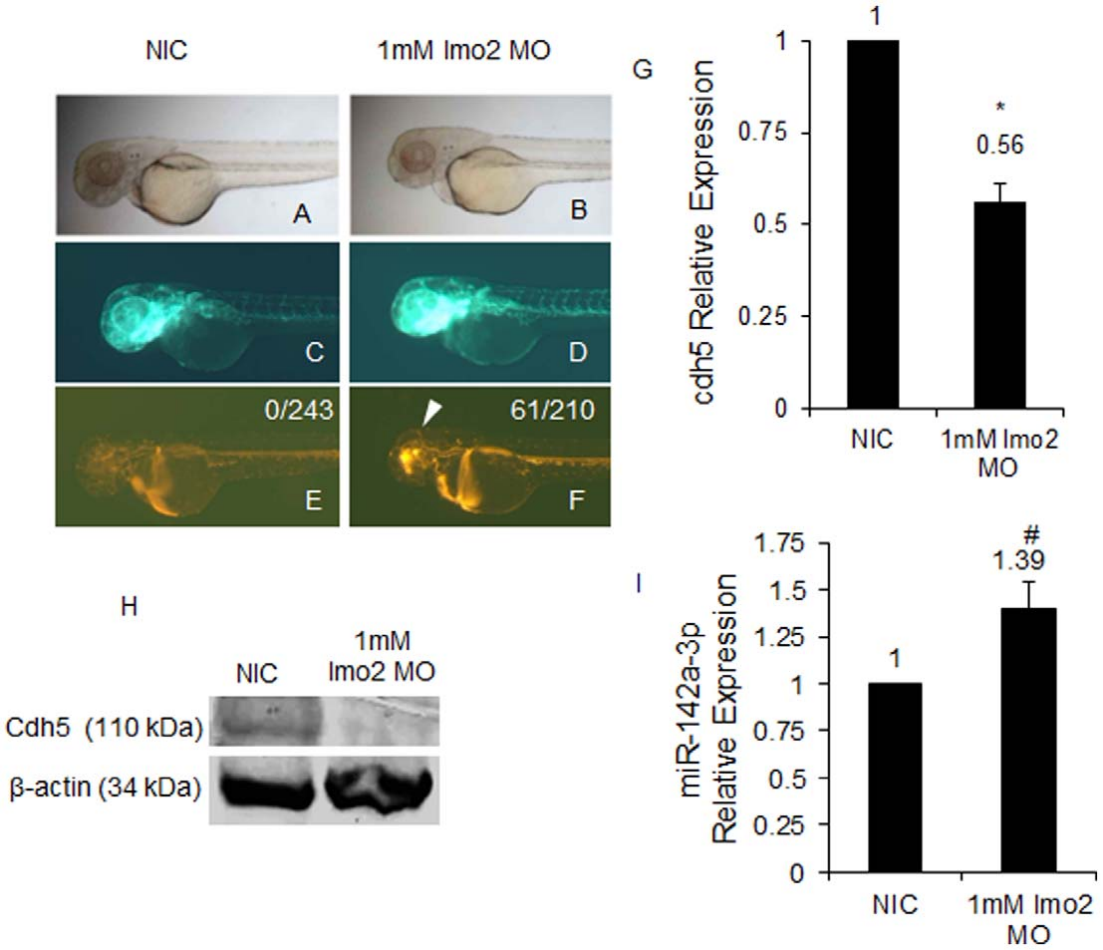
miR-142a-3p and Cdh5 are regulated by the transcription factor Lmo2 in zebrafish. A–F - Morpholino (MO) mediated Lmo2 knockdown induces cerebral hemorrhage phenotype in 2 dpf Tg*(fli1:EGFP, gata1a: dsRed)* zebrafish embryos. A,C,E - non-injected control (NIC) and B,D,F - 1 mM *lmo2* MO injected zebrafish embryos. The embryos were imaged at 2.5× magnification. Arrowheads indicate the site of hemorrhage. G - *cdh5* relative expression quantified by QRT–PCR in *lmo2* knockdown Tg*(fli1:EGFP, gata1a: dsRed)* zebrafish embryos. Data collected from 4 independent experiments is represented as mean fold change ± SD. Asterisk (*) indicates p value of 0.0001 as determined by 2-tailed t-test. H - Western Blot analysis for Cdh5 in 2 dpf NIC and 1 mM *lmo2* MO injected zebrafish embryos. Beta-actin was used as a loading control. I - miR-142a-3p relative expression quantified by QRT–PCR in Lmo2 knockdown zebrafish embryos. The assay was performed as described by manufacturer (QuantiMir kit, SBI, USA). The relative expression of miR-142a-3p was normalized to miR-26a. Data collected from 4 independent experiments is represented as mean fold change ± SD. Hash (#) indicates p value of 0.001 as determined by 2-tailed t-test.

To investigate the role of Lmo2 in mediating transcription of miR-142a and *cdh5*, we injected zebrafish embryos with morpholino to down regulate the endogenous level of Lmo2 protein (Entrez gene ID: 30332). As described previously [34], we have used two ATG morpholinos against *lmo2* gene and observed severe defects in blood cells and blood vessel at 2 dpf Tg*(fli1:EGFP, gata1a: dsRed)* zebrafish embryos. In the double morpholino injected embryos, trunk vessel/SEs are affected and circulating blood cells are reduced in number with anemia as evident by circulating RFP expressing cells and *o-dianisidine* staining respectively (Figure S4). The vascular defects and anemia of the blood cells prevent us from analyzing the vascular integrity phenotype. However a single ATG MO targeted towards the *lmo2* gene generated zebrafish embryos with visible cranial hemorrhage in approximately (61/210) 29% of the MO injected animals (Figure 8A–8F). No visible off-target effects were noticed in the injected embryos. Quantitative real-time PCR analysis in the single MO injected embryos revealed that the expression of *cdh5* decreased by approximately 1.78 fold (0.56±0.05) compared to control and the miR-142a-3p expression increased by approximately 1.39±0.14 fold compared to control (Figure 8G and 8I). We also observed downregulation of *cdh5* protein in *lmo2* MO injected animals (Figure 8H). In summary, we show that knockdown of *lmo2* function in zebrafish embryos leads to an increase in miR-142a-3p transcript levels with a corresponding decrease in the *cdh5* transcript and protein levels.

## Discussion

This study was aimed at elucidating the role of selected miRNAs, conserved between human and zebrafish, in regulating blood vessel development. Of the eight-selected miRNA tested using zebrafish as a model system, we observed specific non-overlapping phenotypes affecting vascular development for three miRNAs, namely miR-1, miR-144 and miR-142a-3p (Figure 2). During zebrafish embryo development, miR-1 is known to express ubiquitously where as miR-144 and miR-142a-3p show strong expression in blood cells [35]. Previous studies of these miRNAs have implicated miR-1 in regulating muscle gene expression and miR-144 in zebrafish embryonic α-globin synthesis [23], [36]. MiR-142a-3p has been shown to regulate RAC1 in hepatocellular carcinoma cells [37]. The negative regulation of miR-142 by the oncogene *lmo2* and its co-factors has also been documented in cell culture models [32]. More recently, the role of miR-142a-3p has been implicated in hematopoiesis, cardiogenesis, and somitegenesis in developing zebrafish embryos through regulating 3′ UTR of *rock2a* transcript [38]. However, to the best of our knowledge distinct functional roles of miR-144, miR-1 and miR-142a-3p in vascular development are yet to be explored. The miRNAs that did not produce any visible vascular phenotypes in our screen include miR-181a, miR-181b, miR-221, miR-222 and miR-451. Recent study on gain-of-function of miR-451 also did not generate a visible phenotype, corroborating with our observations [22]. However the role of miR-451 in erythroid maturation was demonstrated in zebrafish *meunier* mutant background [22]. This suggests that further extensive studies on appropriate transgenic or mutant background may be required to fully understand their role in vascular development.

The results of this study reveal an essential role for miR-142a-3p in angiogenesis and vascular remodeling *in vivo.* The actions of miR-142a-3p reflect its potential to modulate the permeability and remodeling of the endothelium through interaction with endothelial cell-cell adhesion complex. VE-cadherin (*cdh5*) an endothelial-specific transmembrane component of the adherens junction complex and an important regulator of endothelial cell-cell adhesion, serves as a target for repression by miR-142a-3p. In the presence of high levels (overexpression) of miR-142a-3p, *cdh5* levels are suppressed (Figure 7A and 7B). Decreased levels of Cdh5 are known to compromise endothelial cell-cell adhesion thus leading to loss of vascular integrity and remodeling (Figure 4, 7A and 7B) [11]. This effect is rescued, in part by the suppression of miR-142a-3p, restoring proper vascular integrity and remodeling (Figure S2). In the absence (downregulation) of miR-142a-3p, the repressive influence of the miR-142a-3p on *cdh5* is relieved, resulting in abnormal vascular remodeling (Figure 5B –5G, S1 and 7C). Consistent with these findings, zebrafish with impaired *cdh5* function display vascular instability and cranial hemorrhages mimicking those observed in the current study upon suppression of *cdh5* by miR-142a-3p. In our *in silico* analysis, we obtained 52 genes that could potentially bind to miR-142a-3p and influence vascular development (Figure 1, Table S4). In the present study we have tested only *vegfab* and *cdh5* (Figure 6, 7 and S3) and we show that cdh5 gene is one of the bonafied targets of miR-142a-3p in zebarfish embryo. We speculate that endogenous miR-142-3p could have other targets that may be involved in vascular development. Since, it is well documented that miRNAs can regulate multiple genes, the observed vascular phenotype could be a result of miR-142a-3p targeting *cdh5* along with other bonafied target genes. In addition, zebrafish embryos deficient in VE-cad have also been reported to show impair cardiac development because of poor endocardial junction formation resulting in a leak across the endothelial layer [13]. Recently, it has been shown that inhibition of miR-142-3p specifically resulted in abnormal cardiac development in developing zebrafish embryos [38]. Our study also revealed a minor proportion of animals with impaired cardiac development upon suppression of *cdh5* by miR-142a-3p. Collectively, this suggests that miR-142a-3p may also be required for development of heart in zebrafish.

In human cancer studies, VE-cad is known to be up regulated in solid tumors and has been shown to favor angiogenesis and vascular sprouting [39]. In our study we observe that suppression of the repressive influence of miR-142a-3p on *cdh5* also results in higher levels of VE-Cad, leading to abnormal remodeling in the intersegmental vessel (Se) (Figure 5B –5G, 7C and S1B – S1G). Therefore it is possible that the abnormal vascular remodeling observed in the intersegmental vessel (Se) may potentially be a result of abnormal angiogenesis and vascular sprouting (Figure 5D –5H and S1B – S1G). Based on our study, we suggest a potential new function of miR-142-3p in regulating the levels of *cdh5* required for normal angiogenesis and vascular remodeling in zebrafish embryos.

The oncogene *lmo2* has been previously shown to transcriptionally regulate both VE-Cadherin and miR-142 in human cell culture models [32], [33]. Knockdown of Lmo2 expression using a single MO in zebrafish embryos displays a cranial hemorrhage phenotype, similar to those observed upon down regulation of Cdh5 function (Figure 8A–8F) [11]. Animals with impaired *lmo2* function also displayed an increase in miR-142a-3p transcript level and a corresponding decrease in the expression of *cdh5* transcript and protein (Figure 8G–8I). Taken together we speculate that *lmo2*, in addition to its known role as a transcriptional regulator of *CDH5* and miR-142, may also regulates *cdh5* post-transcriptionally through miR-142a-3p in zebrafish embryos. Cdh5 expression is required for tumor angiogenesis and blocking their expression with monoclonal antibodies in mouse tumor models leads to the inhibition of tumor growth [39]. We show that Cdh5 is a potential target of miR-142a-3p and demonstrate that vascular integrity, remodeling and angiogenesis can be influenced through modulation of miR-142a-3p expression, making this a potentially important target for exploring either pro or anti – angiogenesis therapies.

## Materials and Methods

### Ethics Statement

Fish experiments were performed in strict accordance with the recommendations and guidelines laid down by the CSIR Institute of Genomics and Integrative Biology, India. The protocol was approved by the Institutional Animal Ethics Committee (IAEC) of the CSIR Institute of Genomics and Integrative Biology, India (Proposal No 45a). All efforts were made to minimize animal suffering.

### Zebrafish Husbandry

Zebrafish used in this study were housed at the CSIR-Institute of Genomics and Integrative Biology following standard husbandry practices [40]. Wildtype and transgenic zebrafish embryos were obtained by pair wise mating of adult. Tg*(fli1:EGFP)y1* zebrafish line that expresses green fluorescent protein (GFP) in ECs [46] and Tg*(gata1a:dsRed)sd2* that expresses red fluorescent protein in blood cells [47] were used in this study. Double transgenic zebrafish were generated by crossing adult Tg*(fli1:EGFP)y1* and Tg*(gata1a:dsRed)sd2* zebrafish lines.

### Meta-analysis for miRNAs Involved in Vasculature Development

Zebrafish and Human mature miRNA sequences were downloaded from the miRNA database - miRBase version 13.0 (http://www.mirbase.org) [25]. Zebrafish mature miRNA sequences were aligned against Human mature miRNA sequences with stand alone BLAST version 2.2.20 [26]. MiRNAs with 95–100% sequence identity were retained for further analysis. The miRNAs were further analyzed for literature based experimental evidence related to vasculature development. These miRNA were then used for target prediction. A dataset of 672 zebrafish genes with putative expression in blood and blood vessel tissue were retrieved from ZFIN database [27]. The 3′UTR sequences for the 672 zebrafish genes were downloaded from UCSC genome browser Zv8 version [48]. Of the 672 genes retrieved from Zfin, 245 genes did not have any information regarding the 3′ UTR sequences. Therefore these 245 genes were excluded from the analysis. The remaining 427 genes had evidence regarding their 3′ UTR sequences and were included in our analysis. MiRNA target sites were predicted by analyzing the presence of 2–7 miRNA seed sequence in 3′ UTR sequences of 427 genes having putative expression in blood and blood vessel tissues of zebrafish.

### Preparation of miRNA Duplex and Morpholino Oligonucleotide

Single stranded RNA (ssRNA) oligos for miRNA duplex preparation were designed as described [24]. The oligos were obtained from Dharmacon, USA. ssRNA oligos were dissolved in nuclease free water (Ambion, USA) at a concentration of 1 mM prior to use. Oligos were annealed using 5X annealing buffer containing 30 mM HEPES-KOH, pH 7.4, 100 mM KCL, 2 mM MgCl2 and 50 mM NH4Ac. To prepare 20 µM miRNA duplex, 30 µl of each 50 µM ssRNA solution (sense and anti sense) were combined with 15 µl of 5X annealing buffer. Following solutions were incubated at 90°C for 2 min on water bath and allowed for gradual cooling at room temperature for 60 min to make double stranded miRNA duplex. Double stranded miRNA duplexes were stored at −80°C until further use. Morpholino (MO) oligonucleotide (Gene Tools, USA) were dissolved in nuclease free water (Ambion, USA) at a concentration of 1 mM according to the protocols recommended by Gene Tools. 1 mM stocks of MO oligos were stored at –80°C until further use. Working aliquots of MO oligos were prepared and stored at 4°C. Details regarding the oligo sequences and MO are provided in Table S5.

### GFP Reporter Construct

The 3′ UTR of *cdh5* and *vegfab* genes were amplified from cDNA obtained from 2 dpf zebrafish embryos. Primers were designed to amplified 500 bp of *cdh5* 3′UTR (110–610 nts position) containing two predicted miR-142a-3p binding site and 472 bp of *vegfab* 3′ UTR (346–818 nts position) containing two predicted miR-142a-3p binding site respectively. The PCR products were digested with Xho/XbaI and subcloned into pCS 2+ GFP vectors [24]. Cloning of 3X *cdh5* siteA, 3X *cdh5* siteB, 3X *cdh5* mut siteA and 3X *cdh5* mut siteB was accomplished by synthesizing long overlapping oligonucleotide primers. The primers were PCR extended, digested with XhoI/XbaI and subcloned into pCS 2+ GFP. All the GFP reporter constructs were linearized with NotI for *in vitro* transcription by SP6 polymerase using Mmesseage Mmachine kit (Ambion, USA). All constructs were confirmed by sequencing. Details regarding the oligo sequences and genes are provided in Table S5.

### Microinjections into Zebrafish Embryos

GFP reporter constructs**,** miRNA duplexes and MOs injections were performed following a published protocols [24], [29], [41]. Glass capillary (World Precision) micropipettes were pulled using Sutter Instrument (USA) and clipped appropriately to deliver 1–3 nl solution into 1–2 cell zebrafish embryos. For overexpression of miRNAs, one to two nanoliter containing 10–20 µM miRNA duplex was injected at one-cell stage zebrafish embryos. The concentration and dose of miRNA that was microinjected in zebrafish embryos were based on the previous published studies [24], [29], [42]. GFP sensor target validation assays were performed as described previously [22], [24], [42].

### Imaging and Evaluation of Vascular Phenotypes

Fluorescent labeled Tg*(fli1:EGFP, gata1a: dsRed)* embryos were injected as described incubated at 29±1°C. The injected and non-injected control embryos were observed and imaged with an upright Zeiss Axioscope 40 fluorescent microscope (Carl Zeiss, Germany) using 2.5× magnification and 0.075 numerical aperture unless indicated in the figure legend. Embryos were also imaged with optical sectioning microscope using 3D structured light illumination (Carl Zeiss, Germany) where necessary using 10× magnification and 0.3 numerical aperture unless indicated in the figure legend. Confocal microscopy of Tg*(fli1:EGFP, gata1a: dsRed)* animals were performed on Zeiss LSM 510 meta confocal microscope and mounted for imaging on 1% low melting agarose in Danieu’s solution with tricaine. Image stacks were collected with 3.5 µm spacing between planes at 1,024×1,024 pixel resolution. Animals were treated with phenyl thiourea to inhibit pigment formation. Images were processed with Zeiss AxioVision 4.6, ZEN 2011 Zeiss software and Adobe Photoshop CS software. Identical modifications and adjustment were applied to all the images in the same experiment.

### Western Blot

Approximately forty embryos were deyolked in calcium free ringer solution containing 0.1 mM EDTA. Protein preparations were done in lysis buffer containing 1× protease inhibitors (RIPA-PI) and resuspended in 40 µl of RIPA-PI. Protein extracts were quantified by a Bradford protein assay (Sigma, USA) and 40 µg/lane of extract were separated by SDS-PAGE on 4–12% gels. The gels were transferred to 0.45 µm PVDF membranes (MDI, India). The membranes were blocked for overnight in TBS with 3% BSA. Then the membranes were washed with TBS 3 times for 5 min each and incubated at room temperature with rabbit anti-human VE-cadherin antibody (SDI, Newark, DE) at 1∶10,000 in TBS with 3% BSA with constant shaking for 2 hrs. After incubation, membranes were washed with TBS 3 times for 5 min each and incubated with alkaline phosphatase-conjugated anti-rabbit secondary antibody (Santa-Cruz, USA). Membranes were developed by treating with NBT/BCIP substrate. Membranes were subsequently stripped and left in blocking solutions overnight. The membranes were then re-probed with goat anti-actin antibody (Santa-Cruz, USA) in a similar fashion.

### Quantitative Real-Time PCR (QRT-PCR)

RNA derived from treated and control embryos were obtained using TRIZOL reagent (Invitrogen, USA) according to protocol previously described [43]. Two microgram of RNA was reverse transcribed into cDNA using Superscript II (Invitrogen, USA), and 2 µl of reverse transcription reaction was used for mRNA qPCR using Sybr Green mix (Roche, Germany). For miRNA qPCR, 1 µg of total RNA was used for reverse transcription (RT) following the manufacturer’s protocol (QuantiMir kit, SBI, USA). RT products were then used for qPCR using mature miR-142a-3p sequence as forward primer together with the universal (reverse) primer provided with the kit. The relative expression of miR-142a-3p was normalized to miR-26a [44]. QRT-PCR detection was done using Roche Lightcycler LC 480. The oligo sequences are described in Table S5.

### FACS Analysis and RNA Isolation

Tg*(fli1:EGFP, gata1a: dsRed)* embryos at 2 dpf were deyolked by pipetting in calcium free Ringer’s solution and transferred to 29°C pre-warmed 1 ml 0.25% trypsin (phenol red free, Invitrogen). Then 27 µl collagenase (100 mg/1 ml HBSS, Invitrogen) was added to 0.25% trypsin containing embryo and homogenized by pipetting every 5 min for 15–20 min at 29°C to dissociate the cells. Dissociated cells were collected in ice cold PBS by centrifuging at 350 g for 5 min at 4°C followed by two time washing with ice cold PBS. Cellular debris were removed by passing the cell suspension through 40 µm BD falcon cell strainer (cat no-352340). Cell sorting was performed at Institute of Genomics and Integrative Biology Cell Sorter Facility using BD Facs Aria III. Purity of the sorted cells was tested by resorting GFP (+) and GFP (–) cells after first sort. Sorted cells were resuspended into Trizol and total RNA isolated using miRNeasy Mini Qiagen Kit (cat no-217004).

## Supporting Information

Figure S1 **Microinjection of miR-142a-3p morpholino (MO) in zebrafish embryos in Tg** ***(fli1:EGFP, gata1a: dsRed)*** **leads to intersegmental vessel (Se) remodeling.** A - Graphical representation of dose dependent microinjection ranging from 10–500 µM of miR-142a-3p morpholino (MO) in 28–30 hpf Tg*(fli1:EGFP, gata1a: dsRed)* zebrafish embryos. Bar graph showing percentage of embryos with survival (grey) and intersegmental vessel (Se) defect (black). Numbers of embryos analyzed are indicated in parenthesis. B–G Representative image of Tg*(fli1:EGFP, gata1a: dsRed)* zebrafish embryos displaying intersegmental vessels (Se) from non-injected control and 200 µM miR-142a-3p morpholino injected embryos at different developmental stages. Images are arranged in a lateral view and displaying intersegmental vessels (Se) from the trunk region. Arrowheads indicate regions with vascular defects.
(TIF)

Figure S2 **Rescue of miR-142a-3p duplex induced hemorrhage phenotype in zebrafish embryos using antisense morpholino targeting to the mature form of miR-142a-3p.** A - Bar graph showing cerebral hemorrhage phenotype in non-injected control (NIC); 10 µM miR-142a-3p duplex injected; co-injection of 10 µM miR-142a-3p duplex with 100 µM miR-142a-3p morpholino (MO); and 100 µM miR-142a-3p MO injected Tg*(fli1:EGFP, gata1a: dsRed)* zebrafish embryos at 2dpf. Data is represented as mean percentage ± SD (standard deviation) collected over 3 independent experiments. n represents the number embryos analyzed. B-I - Representative images of cerebral hemorrhage phenotype in Tg*(fli1:EGFP, gata1a: dsRed)* zebrafish embryos at 2 dpf. B and C - NIC embryos. D and E - 10 µM miR-142a-3p duplex injected embryos. F and G – Embryos receiving co-injection of 10 µM miR-142a-3p duplex with 100 µM miR-142a-3p MO. H and I - 100 µM miR-142a-3p MO injected embryos. The embryos were imaged at 2.5× magnification. Arrowheads indicate the site of hemorrhage.
(TIF)

Figure S3 **GFP sensor target validation assay for** ***vegfab*** **3′UTR.** A - Schematic of GFP-*vegabf* 3′UTR transcript containing two miR-142a-3p predicted binding site (site “A”, blue; site “B”, purple). Predicted seed complementarity sequences are boxed in red colour. B - Silencing effect of miR-142a-3p on the GFP-*vegfab* 3′UTR gene target. Co-injection of the GFP-*vegfab* 3′UTR mRNA and miR-142a-3p led to moderate suppression of GFP expression in wildtype zebrafish embryos. Expressions of the reporters were analyzed at 26 to 28 hours post fertilization. DsRed mRNA was used as injection controls and is shown in the lower panel. Upper panel displays GFP-*vegfab* 3′UTR sensor RNA and miR-142a-3p combinations. Group images of embryos that were co-injected of various GFP-*vegfab* 3′UTR mRNA and miR-142a-3p are compared with embryos receiving respective GFP-*vegfab* 3′UTR sensor only. Clutch of representative embryos (7–9 embryos in each image) were photographed together in a single image to ensure valid comparison of relative green fluorescent intensity between two groups. The embryos were imaged at 2.5× magnification.
(TIF)

Figure S4 **Microinjection of** ***lmo2*** **1&2 morpholino (MO) in Tg** ***(fli1:EGFP, gata1a: dsRed)*** **zebrafish embryos.** A-H - Morpholino (MO) mediated Lmo2 knockdown induces blood cells and blood vessel defects in 2 dpf Tg*(fli1:EGFP, gata1a: dsRed)* zebrafish embryos. A–D - non-injected control (NIC) and E – H 1.75 ng *lmo2* 1&2 MO injected zebrafish embryos. A–C, E – G Apotome microscope images (GFP/RFP merged) of lmo2 1&2 MO injected Tg*(fli1:EGFP, gata1a: dsRed)* embryos at 2 dpf (Lateral View). A–C - non-injected control embryos and E – G –lmo2 1&2 MO injected embryos. D, H - Embryos stained with *o-dianisidine*. The embryos were imaged at 10× magnification (A–C, E–G) and 2.5× magnification (D, H). Arrowheads indicate the site of defects.
(TIF)

Figure S5 **Bioactivity of GFP sensor with 4**× **miR-142a-3p perfect complimentary target (PT) sites and miRNA duplex.** A - Schematic of miRNA target validation GFP sensor assay. B - Schematic of miR-142a-3p perfect target (PT) sequence complementary with miR-142a-3p sequence. C - Silencing effect of miR-142a-3p on the GFP-4X PT 3′UTR gene target. Co-injection of the miR-142a-3p and GFP-4X PT 3′UTR mRNA led to suppression of GFP expression in wildtype zebrafish embryos. Expressions of the reporters were analyzed at 26 to 28 hours post fertilization. Bright-field image of embryos are shown in the upper row of each panel. DsRed mRNA was used as injection control and is shown in the lower row of each panel. In the middle row of each panel various GFP-4X PT 3′UTR sensor RNA and miR-142a-3p combinations were tested as labeled. Group of images in which embryos with co-injection of various GFP-4X PT 3′UTR mRNA and miR-142a-3p images are compared with embryos receiving respective GFP-4X PT 3′UTR sensor only. Clutch of representative embryos (7–9 embryos in each image) were photographed together in a single image to ensure valid comparison of relative green fluorescent intensity between two groups. The embryos were imaged at 2.5× magnification.
(TIF)

Table S1 MiRNAs that have 95–100% sequence conservation between zebrafish and human.
(DOC)

Table S2 MiRNAs involved in vasculature development.
(DOCX)

Table S3 A dataset of 672 zebrafish genes with putative expression in blood and blood vessel tissue
(DOC)

Table S4 Zebrafish putative miRNA-gene target pairs derived from miRNA seed sequence match analysis.
(DOC)

Table S5 The list of oligos sequence used in the study
(DOC)
