# Supplementary figures and images for: Reverse Genetics Screen in Zebrafish Identifies a Role of miR-142a-3p in Vascular Development and Integrity
Source: PLoS One. 2012 Dec 21;7(12):e52588. doi: 10.1371/journal.pone.0052588 (PMC3528674; doi:10.1371/journal.pone.0052588)

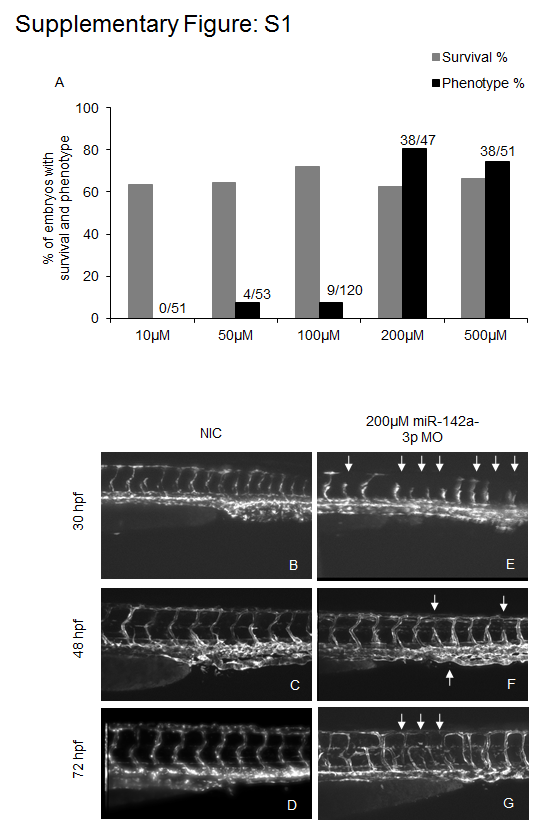

Supplement: Figure S1 — Microinjection of miR-142a-3p morpholino (MO) in zebrafish embryos in Tg (fli1:EGFP, gata1a: dsRed) leads to intersegmental vessel (Se) remodeling. A - Graphical representation of dose dependent microinjection ranging from 10–500 µM of miR-142a-3p morpholino (MO) in 28–30 hpf Tg(fli1:EGFP, gata1a: dsRed) zebrafish embryos. Bar graph showing percentage of embryos with survival (grey) and intersegmental vessel (Se) defect (black). Numbers of embryos analyzed are indicated in parenthesis. B–G Representative image of Tg(fli1:EGFP, gata1a: dsRed) zebrafish embryos displaying intersegmental vessels (Se) from non-injected control and 200 µM miR-142a-3p morpholino injected embryos at different developmental stages. Images are arranged in a lateral view and displaying intersegmental vessels (Se) from the trunk region. Arrowheads indicate regions with vascular defects. (TIF) [file pone.0052588.s001.tif]

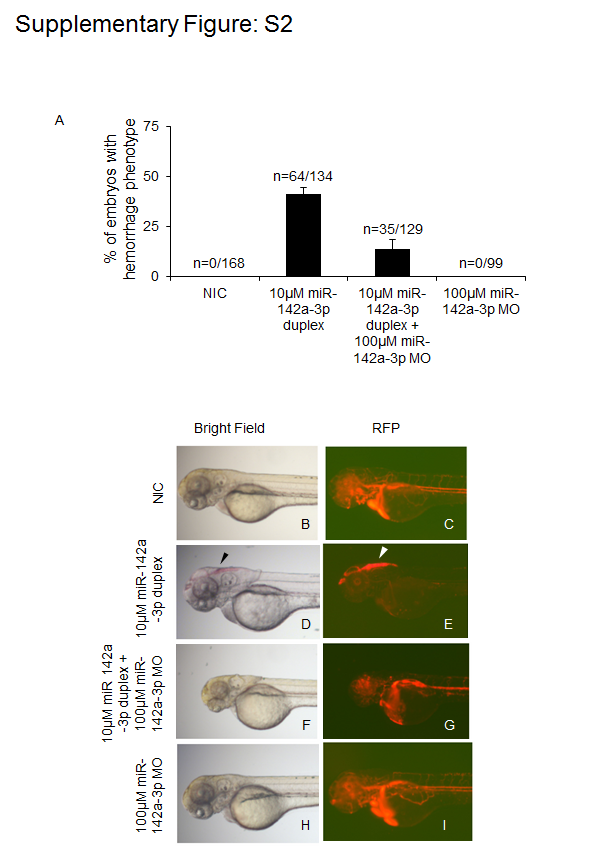

Supplement: Figure S2 — Rescue of miR-142a-3p duplex induced hemorrhage phenotype in zebrafish embryos using antisense morpholino targeting to the mature form of miR-142a-3p. A - Bar graph showing cerebral hemorrhage phenotype in non-injected control (NIC); 10 µM miR-142a-3p duplex injected; co-injection of 10 µM miR-142a-3p duplex with 100 µM miR-142a-3p morpholino (MO); and 100 µM miR-142a-3p MO injected Tg(fli1:EGFP, gata1a: dsRed) zebrafish embryos at 2dpf. Data is represented as mean percentage ± SD (standard deviation) collected over 3 independent experiments. n represents the number embryos analyzed. B-I - Representative images of cerebral hemorrhage phenotype in Tg(fli1:EGFP, gata1a: dsRed) zebrafish embryos at 2 dpf. B and C - NIC embryos. D and E - 10 µM miR-142a-3p duplex injected embryos. F and G – Embryos receiving co-injection of 10 µM miR-142a-3p duplex with 100 µM miR-142a-3p MO. H and I - 100 µM miR-142a-3p MO injected embryos. The embryos were imaged at 2.5× magnification. Arrowheads indicate the site of hemorrhage. (TIF) [file pone.0052588.s002.tif]

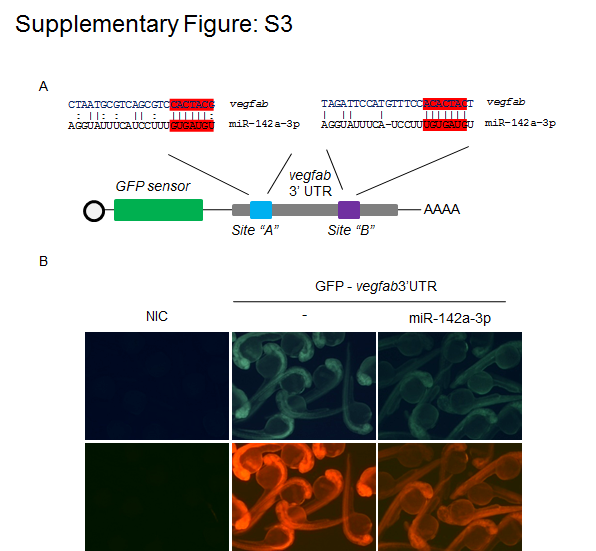

Supplement: Figure S3 — GFP sensor target validation assay for vegfab 3′UTR. A - Schematic of GFP-vegabf 3′UTR transcript containing two miR-142a-3p predicted binding site (site “A”, blue; site “B”, purple). Predicted seed complementarity sequences are boxed in red colour. B - Silencing effect of miR-142a-3p on the GFP-vegfab 3′UTR gene target. Co-injection of the GFP-vegfab 3′UTR mRNA and miR-142a-3p led to moderate suppression of GFP expression in wildtype zebrafish embryos. Expressions of the reporters were analyzed at 26 to 28 hours post fertilization. DsRed mRNA was used as injection controls and is shown in the lower panel. Upper panel displays GFP-vegfab 3′UTR sensor RNA and miR-142a-3p combinations. Group images of embryos that were co-injected of various GFP-vegfab 3′UTR mRNA and miR-142a-3p are compared with embryos receiving respective GFP-vegfab 3′UTR sensor only. Clutch of representative embryos (7–9 embryos in each image) were photographed together in a single image to ensure valid comparison of relative green fluorescent intensity between two groups. The embryos were imaged at 2.5× magnification. (TIF) [file pone.0052588.s003.tif]

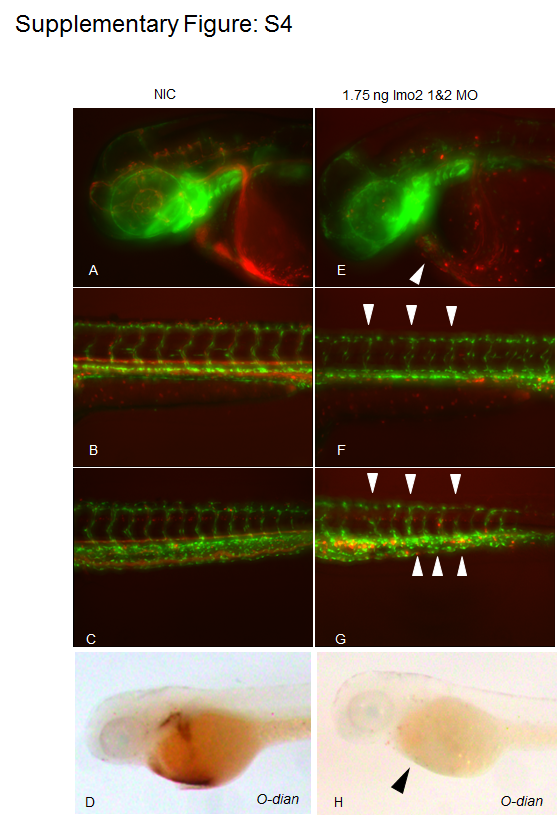

Supplement: Figure S4 — Microinjection of lmo2 1&2 morpholino (MO) in Tg (fli1:EGFP, gata1a: dsRed) zebrafish embryos. A-H - Morpholino (MO) mediated Lmo2 knockdown induces blood cells and blood vessel defects in 2 dpf Tg(fli1:EGFP, gata1a: dsRed) zebrafish embryos. A–D - non-injected control (NIC) and E – H 1.75 ng lmo2 1&2 MO injected zebrafish embryos. A–C, E – G Apotome microscope images (GFP/RFP merged) of lmo2 1&2 MO injected Tg(fli1:EGFP, gata1a: dsRed) embryos at 2 dpf (Lateral View). A–C - non-injected control embryos and E – G –lmo2 1&2 MO injected embryos. D, H - Embryos stained with o-dianisidine. The embryos were imaged at 10× magnification (A–C, E–G) and 2.5× magnification (D, H). Arrowheads indicate the site of defects. (TIF) [file pone.0052588.s004.tif]

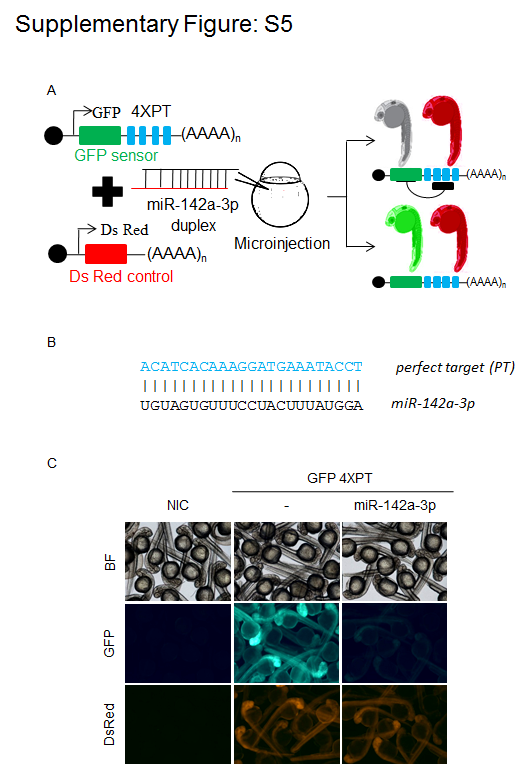

Supplement: Figure S5 — Bioactivity of GFP sensor with 4× miR-142a-3p perfect complimentary target (PT) sites and miRNA duplex. A - Schematic of miRNA target validation GFP sensor assay. B - Schematic of miR-142a-3p perfect target (PT) sequence complementary with miR-142a-3p sequence. C - Silencing effect of miR-142a-3p on the GFP-4X PT 3′UTR gene target. Co-injection of the miR-142a-3p and GFP-4X PT 3′UTR mRNA led to suppression of GFP expression in wildtype zebrafish embryos. Expressions of the reporters were analyzed at 26 to 28 hours post fertilization. Bright-field image of embryos are shown in the upper row of each panel. DsRed mRNA was used as injection control and is shown in the lower row of each panel. In the middle row of each panel various GFP-4X PT 3′UTR sensor RNA and miR-142a-3p combinations were tested as labeled. Group of images in which embryos with co-injection of various GFP-4X PT 3′UTR mRNA and miR-142a-3p images are compared with embryos receiving respective GFP-4X PT 3′UTR sensor only. Clutch of representative embryos (7–9 embryos in each image) were photographed together in a single image to ensure valid comparison of relative green fluorescent intensity between two groups. The embryos were imaged at 2.5× magnification. (TIF) [file pone.0052588.s005.tif]
