# Supplementary material for: Reverse Genetics Screen in Zebrafish Identifies a Role of miR-142a-3p in Vascular Development and Integrity
Source: PLoS One. 2012 Dec 21;7(12):e52588. doi: 10.1371/journal.pone.0052588 (PMC3528674; doi:10.1371/journal.pone.0052588)
Supplement: Table S1 — MiRNAs that have 95–100% sequence conservation between zebrafish and human. (DOC) [file pone.0052588.s006.doc]

**Supplementary** **Table S1:**

MicroRNAs that have 95 -100% sequence conservation between zebrafish and human.

| **S.no.** | **Zebrafish miRNAs** | **Human**  **miRNAs** | **Score** | **E value** | **Identity** |
| --- | --- | --- | --- | --- | --- |
|  | dre‐mir‐103 | hsa‐mir‐103 | 46.1 | 7.00E‐10 | 23/23 (100%) |
|  | dre‐mir‐107 | hsa‐mir‐107 | 46.1 | 7.00E‐10 | 23/23 (100%) |
|  | dre‐mir‐10b | hsa‐mir‐10b | 46.1 | 7.00E‐10 | 23/23 (100%) |
|  | dre‐mir‐135a | hsa‐mir‐135a | 46.1 | 7.00E‐10 | 23/23 (100%) |
|  | dre‐mir‐142a‐3p | hsa‐mir‐142‐3p | 46.1 | 7.00E‐10 | 23/23 (100%) |
|  | dre‐mir‐181a | hsa‐mir‐181a | 46.1 | 7.00E‐10 | 23/23 (100%) |
|  | dre‐mir‐199 | hsa‐mir‐199a‐5p | 46.1 | 7.00E‐10 | 23/23 (100%) |
|  | dre‐mir‐19b | hsa‐mir‐19b | 46.1 | 7.00E‐10 | 23/23 (100%) |
|  | dre‐mir‐20a | hsa‐mir‐20a | 46.1 | 7.00E‐10 | 23/23 (100%) |
|  | dre‐mir‐221 | hsa‐mir‐221 | 46.1 | 7.00E‐10 | 23/23 (100%) |
|  | dre‐mir‐7a | hsa‐mir‐7 | 46.1 | 7.00E‐10 | 23/23 (100%) |
|  | dre‐mir‐9 | hsa‐mir‐9 | 46.1 | 7.00E‐10 | 23/23 (100%) |
|  | dre‐mir‐96 | hsa‐mir‐96 | 46.1 | 7.00E‐10 | 23/23 (100%) |
|  | dre‐let‐7a | hsa‐let‐7a | 44.1 | 3.00E‐09 | 22/22 (100%) |
|  | dre‐let‐7b | hsa‐let‐7b | 44.1 | 3.00E‐09 | 22/22 (100%) |
|  | dre‐let‐7c | hsa‐let‐7c | 44.1 | 3.00E‐09 | 22/22 (100%) |
|  | dre‐let‐7f | hsa‐let‐7f | 44.1 | 3.00E‐09 | 22/22 (100%) |
|  | dre‐let‐7i | hsa‐let‐7i | 44.1 | 3.00E‐09 | 22/22 (100%) |
|  | dre‐mir‐1 | hsa‐mir‐1 | 44.1 | 3.00E‐09 | 22/22 (100%) |
|  | dre‐mir‐100 | hsa‐mir‐100 | 44.1 | 3.00E‐09 | 22/22 (100%) |
|  | dre‐mir‐10a | hsa‐mir‐10a | 44.1 | 3.00E‐09 | 22/22 (100%) |
|  | dre‐mir‐122 | hsa‐mir‐122 | 44.1 | 3.00E‐09 | 22/22 (100%) |
|  | dre‐mir‐125b | hsa‐mir‐125b | 44.1 | 3.00E‐09 | 22/22 (100%) |
|  | dre‐mir‐129* | hsa‐mir‐129‐3p | 44.1 | 3.00E‐09 | 22/22 (100%) |
|  | dre‐mir‐130a | hsa‐mir‐130a | 44.1 | 3.00E‐09 | 22/22 (100%) |
|  | dre‐mir‐132 | hsa‐mir‐132 | 44.1 | 3.00E‐09 | 22/22 (100%) |
|  | dre‐mir‐133a | hsa‐mir‐133a | 44.1 | 3.00E‐09 | 22/22 (100%) |
|  | dre‐mir‐133b | hsa‐mir‐133b | 44.1 | 3.00E‐09 | 22/22 (100%) |
|  | dre‐mir‐137 | hsa‐mir‐137 | 44.1 | 3.00E‐09 | 22/22 (100%) |
|  | dre‐mir‐138 | hsa‐mir‐138 | 44.1 | 3.00E‐09 | 22/22 (100%) |
|  | dre‐mir‐140 | hsa‐mir‐140‐5p | 44.1 | 3.00E‐09 | 22/22 (100%) |
|  | dre‐mir‐145 | hsa‐mir‐145 | 44.1 | 3.00E‐09 | 22/22 (100%) |
|  | dre‐mir‐153a | hsa‐mir‐153 | 44.1 | 3.00E‐09 | 22/22 (100%) |
|  | dre‐mir‐155 | hsa‐mir‐155 | 44.1 | 3.00E‐09 | 22/22 (100%) |
|  | dre‐mir‐17a | hsa‐mir‐17 | 44.1 | 3.00E‐09 | 22/22 (100%) |
|  | dre‐mir‐181a* | hsa‐mir‐181a* | 44.1 | 3.00E‐09 | 22/22 (100%) |
|  | dre‐mir‐181b | hsa‐mir‐181b | 44.1 | 3.00E‐09 | 22/22 (100%) |
|  | dre‐mir‐182 | hsa‐mir‐182 | 44.1 | 3.00E‐09 | 22/22 (100%) |
|  | dre‐mir‐183 | hsa‐mir‐183 | 44.1 | 3.00E‐09 | 22/22 (100%) |
|  | dre‐mir‐18a | hsa‐mir‐18a | 44.1 | 3.00E‐09 | 22/22 (100%) |
|  | dre‐mir‐190 | hsa‐mir‐190 | 44.1 | 3.00E‐09 | 22/22 (100%) |
|  | dre‐mir‐193a | hsa‐mir‐193a‐3p | 44.1 | 3.00E‐09 | 22/22 (100%) |
|  | dre‐mir‐196a | hsa‐mir‐196a | 44.1 | 3.00E‐09 | 22/22 (100%) |
|  | dre‐mir‐200a | hsa‐mir‐200a | 44.1 | 3.00E‐09 | 22/22 (100%) |
|  | dre‐mir‐200b | hsa‐mir‐200b | 44.1 | 3.00E‐09 | 22/22 (100%) |
|  | dre‐mir‐204 | hsa‐mir‐204 | 44.1 | 3.00E‐09 | 22/22 (100%) |
|  | dre‐mir‐205 | hsa‐mir‐205 | 44.1 | 3.00E‐09 | 22/22 (100%) |
|  | dre‐mir‐206 | hsa‐mir‐206 | 44.1 | 3.00E‐09 | 22/22 (100%) |
|  | dre‐mir‐216a | hsa‐mir‐216a | 44.1 | 3.00E‐09 | 22/22 (100%) |
|  | dre‐mir‐217 | hsa‐mir‐217 | 44.1 | 3.00E‐09 | 22/22 (100%) |
|  | dre‐mir‐24 | hsa‐mir‐24 | 44.1 | 3.00E‐09 | 22/22 (100%) |
|  | dre‐mir‐25 | hsa‐mir‐25 | 44.1 | 3.00E‐09 | 22/22 (100%) |
|  | dre‐mir‐26a | hsa‐mir‐26a | 44.1 | 3.00E‐09 | 22/22 (100%) |
|  | dre‐mir‐29a | hsa‐mir‐29c | 44.1 | 3.00E‐09 | 22/22 (100%) |
|  | dre‐mir‐29b | hsa‐mir‐29b | 44.1 | 3.00E‐09 | 22/22 (100%) |
|  | dre‐mir‐301a | hsa‐mir‐301a | 44.1 | 3.00E‐09 | 22/22 (100%) |
|  | dre‐mir‐30b | hsa‐mir‐30b | 44.1 | 3.00E‐09 | 22/22 (100%) |
|  | dre‐mir‐30c | hsa‐mir‐30c | 44.1 | 3.00E‐09 | 22/22 (100%) |
|  | dre‐mir‐30d | hsa‐mir‐30d | 44.1 | 3.00E‐09 | 22/22 (100%) |
|  | dre‐mir‐30e | hsa‐mir‐30e | 44.1 | 3.00E‐09 | 22/22 (100%) |
|  | dre‐mir‐30e* | hsa‐mir‐30a* | 44.1 | 3.00E‐09 | 22/22 (100%) |
|  | dre‐mir‐338 | hsa‐mir‐338‐3p | 44.1 | 3.00E‐09 | 22/22 (100%) |
|  | dre‐mir‐34 | hsa‐mir‐34a | 44.1 | 3.00E‐09 | 22/22 (100%) |
|  | dre‐mir‐363 | hsa‐mir‐363 | 44.1 | 3.00E‐09 | 22/22 (100%) |
|  | dre‐mir‐365 | hsa‐mir‐365 | 44.1 | 3.00E‐09 | 22/22 (100%) |
|  | dre‐mir‐451 | hsa‐mir‐451 | 44.1 | 3.00E‐09 | 22/22 (100%) |
|  | dre‐mir‐454b | hsa‐mir‐454 | 44.1 | 3.00E‐09 | 22/22 (100%) |
|  | dre‐mir‐92a | hsa‐mir‐92a | 44.1 | 3.00E‐09 | 22/22 (100%) |
|  | dre‐mir‐92b | hsa‐mir‐92b | 44.1 | 3.00E‐09 | 22/22 (100%) |
|  | dre‐mir‐99 | hsa‐mir‐99a | 44.1 | 3.00E‐09 | 22/22 (100%) |
|  | dre‐mir‐126 | hsa‐mir‐126 | 42.1 | 1.00E‐08 | 21/21 (100%) |
|  | dre‐mir‐126* | hsa‐mir‐126* | 42.1 | 1.00E‐08 | 21/21 (100%) |
|  | dre‐mir‐128 | hsa‐mir‐128 | 42.1 | 1.00E‐08 | 21/21 (100%) |
|  | dre‐mir‐129 | hsa‐mir‐129‐5p | 42.1 | 1.00E‐08 | 21/21 (100%) |
|  | dre‐mir‐140* | hsa‐mir‐140‐3p | 42.1 | 1.00E‐08 | 21/21 (100%) |
|  | dre‐mir‐141 | hsa‐mir‐200a | 42.1 | 1.00E‐08 | 21/21 (100%) |
|  | dre‐mir‐142a‐5p | hsa‐mir‐142‐5p | 42.1 | 1.00E‐08 | 21/21 (100%) |
|  | dre‐mir‐143 | hsa‐mir‐143 | 42.1 | 1.00E‐08 | 21/21 (100%) |
|  | dre‐mir‐181c | hsa‐mir‐181b | 42.1 | 1.00E‐08 | 21/21 (100%) |
|  | dre‐mir‐182* | hsa‐mir‐182* | 42.1 | 1.00E‐08 | 21/21 (100%) |
|  | dre‐mir‐184 | hsa‐mir‐184 | 42.1 | 1.00E‐08 | 21/21 (100%) |
|  | dre‐mir‐194a | hsa‐mir‐194 | 42.1 | 1.00E‐08 | 21/21 (100%) |
|  | dre‐mir‐199* | hsa‐mir‐199b‐3p | 42.1 | 1.00E‐08 | 21/21 (100%) |
|  | dre‐mir‐19c | hsa‐mir‐19b | 42.1 | 1.00E‐08 | 21/21 (100%) |
|  | dre‐mir‐200c | hsa‐mir‐200b | 42.1 | 1.00E‐08 | 21/21 (100%) |
|  | dre‐mir‐214 | hsa‐mir‐214 | 42.1 | 1.00E‐08 | 21/21 (100%) |
|  | dre‐mir‐218a | hsa‐mir‐218 | 42.1 | 1.00E‐08 | 21/21 (100%) |
|  | dre‐mir‐219 | hsa‐mir‐219‐5p | 42.1 | 1.00E‐08 | 21/21 (100%) |
|  | dre‐mir‐222 | hsa‐mir‐222 | 42.1 | 1.00E‐08 | 21/21 (100%) |
|  | dre‐mir‐223 | hsa‐mir‐223 | 42.1 | 1.00E‐08 | 21/21 (100%) |
|  | dre‐mir‐23a | hsa‐mir‐23a | 42.1 | 1.00E‐08 | 21/21 (100%) |
|  | dre‐mir‐23b | hsa‐mir‐23b | 42.1 | 1.00E‐08 | 21/21 (100%) |
|  | dre‐mir‐27a | hsa‐mir‐27a | 42.1 | 1.00E‐08 | 21/21 (100%) |
|  | dre‐mir‐499 | hsa‐mir‐499‐5p | 42.1 | 1.00E‐08 | 21/21 (100%) |
|  | dre‐mir‐9* | hsa‐mir‐9* | 42.1 | 1.00E‐08 | 21/21 (100%) |
|  | dre‐mir‐124 | hsa‐mir‐124 | 40.1 | 4.00E‐08 | 20/20 (100%) |
|  | dre‐mir‐144 | hsa‐mir‐144 | 40.1 | 4.00E‐08 | 20/20 (100%) |
|  | dre‐mir‐16a | hsa‐mir‐16 | 40.1 | 4.00E‐08 | 20/20 (100%) |
|  | dre‐mir‐16b | hsa‐mir‐16 | 40.1 | 4.00E‐08 | 20/20 (100%) |
|  | dre‐mir‐187 | hsa‐mir‐187 | 40.1 | 4.00E‐08 | 20/20 (100%) |
|  | dre‐mir‐192 | hsa‐mir‐192 | 40.1 | 5.00E‐08 | 20/20 (100%) |
|  | dre‐mir‐19a* | hsa‐mir‐19a* | 40.1 | 4.00E‐08 | 20/20 (100%) |
|  | dre‐mir‐203a | hsa‐mir‐203 | 40.1 | 4.00E‐08 | 20/20 (100%) |
|  | dre‐mir‐210 | hsa‐mir‐210 | 40.1 | 4.00E‐08 | 20/20 (100%) |
|  | dre‐mir‐218b | hsa‐mir‐218 | 40.1 | 4.00E‐08 | 20/20 (100%) |
|  | dre‐mir‐26b | hsa‐mir‐26a | 40.1 | 4.00E‐08 | 20/20 (100%) |
|  | dre‐mir‐375 | hsa‐mir‐375 | 40.1 | 4.00E‐08 | 20/20 (100%) |
|  | dre‐mir‐10d | hsa‐mir‐10b | 38.2 | 2.00E‐07 | 22/23 (95%) |
|  | dre‐mir‐135b | hsa‐mir‐135a | 38.2 | 2.00E‐07 | 19/19 (100%) |
|  | dre‐mir‐146a | hsa‐mir‐146b‐5p | 38.2 | 2.00E‐07 | 19/19 (100%) |
|  | dre‐mir‐152 | hsa‐mir‐152 | 38.2 | 2.00E‐07 | 19/19 (100%) |
|  | dre‐mir‐15b | hsa‐mir‐15b | 38.2 | 2.00E‐07 | 19/19 (100%) |
|  | dre‐mir‐19a | hsa‐mir‐19b | 38.2 | 2.00E‐07 | 22/23 (95%) |
|  | dre‐mir‐19d | hsa‐mir‐19b | 38.2 | 2.00E‐07 | 22/23 (95%) |
|  | dre‐mir‐20b | hsa‐mir‐20b | 38.2 | 2.00E‐07 | 22/23 (95%) |
|  | dre‐mir‐27d | hsa‐mir‐27b | 38.2 | 2.00E‐07 | 19/19 (100%) |
|  | dre‐mir‐301b | hsa‐mir‐301a | 38.2 | 2.00E‐07 | 19/19 (100%) |
|  | dre‐mir‐301c | hsa‐mir‐301a | 38.2 | 2.00E‐07 | 19/19 (100%) |
|  | dre‐let‐7d | hsa‐let‐7c | 36.2 | 7.00E‐07 | 21/22 (95%) |
|  | dre‐let‐7g | hsa‐let‐7g | 36.2 | 7.00E‐07 | 21/22 (95%) |
|  | dre‐let‐7j | hsa‐let‐7g | 36.2 | 7.00E‐07 | 21/22 (95%) |
|  | dre‐mir‐10c | hsa‐mir‐10a | 36.2 | 7.00E‐07 | 21/22 (95%) |
|  | dre‐mir‐125c | hsa‐mir‐125b | 36.2 | 7.00E‐07 | 21/22 (95%) |
|  | dre‐mir‐130b | hsa‐mir‐130b | 36.2 | 7.00E‐07 | 21/22 (95%) |
|  | dre‐mir‐130c | hsa‐mir‐130a | 36.2 | 7.00E‐07 | 21/22 (95%) |
|  | dre‐mir‐133c | hsa‐mir‐133b | 36.2 | 7.00E‐07 | 21/22 (95%) |
|  | dre‐mir‐135c | hsa‐mir‐135a | 36.2 | 7.00E‐07 | 21/22 (95%) |
|  | dre‐mir‐148 | hsa‐mir‐148b | 36.2 | 7.00E‐07 | 21/22 (95%) |
|  | dre‐mir‐150 | hsa‐mir‐150 | 36.2 | 7.00E‐07 | 21/22 (95%) |
|  | dre‐mir‐153c | hsa‐mir‐153 | 36.2 | 7.00E‐07 | 21/22 (95%) |
|  | dre‐mir‐15a | hsa‐mir‐15a | 36.2 | 7.00E‐07 | 21/22 (95%) |
|  | dre‐mir‐18b | hsa‐mir‐18a | 36.2 | 7.00E‐07 | 21/22 (95%) |
|  | dre‐mir‐18b* | hsa‐mir‐18a* | 36.2 | 7.00E‐07 | 21/22 (95%) |
|  | dre‐mir‐193b | hsa‐mir‐193b | 36.2 | 7.00E‐07 | 21/22 (95%) |
|  | dre‐mir‐196b | hsa‐mir‐196a | 36.2 | 7.00E‐07 | 21/22 (95%) |
|  | dre‐mir‐202* | hsa‐mir‐202* | 36.2 | 7.00E‐07 | 21/22 (95%) |
|  | dre‐mir‐22a | hsa‐mir‐22 | 36.2 | 7.00E‐07 | 21/22 (95%) |
|  | dre‐mir‐22b | hsa‐mir‐22 | 36.2 | 7.00E‐07 | 21/22 (95%) |
|  | dre‐mir‐27e | hsa‐mir‐27b | 36.2 | 7.00E‐07 | 18/18 (100%) |
|  | dre‐mir‐30a | hsa‐mir‐30d | 36.2 | 7.00E‐07 | 21/22 (95%) |
|  | dre‐mir‐34b | hsa‐mir‐34c‐5p | 36.2 | 7.00E‐07 | 21/22 (95%) |
|  | dre‐mir‐454a | hsa‐mir‐454 | 36.2 | 7.00E‐07 | 21/22 (95%) |
|  | dre‐mir‐455 | hsa‐mir‐455‐5p | 36.2 | 7.00E‐07 | 21/22 (95%) |
|  | dre‐mir‐101b | hsa‐mir‐101 | 34.2 | 3.00E‐06 | 20/21 (95%) |
|  | dre‐mir‐142b‐5p | hsa‐mir‐142‐5p | 34.2 | 3.00E‐06 | 20/21 (95%) |
|  | dre‐mir‐146b | hsa‐mir‐146a | 34.2 | 3.00E‐06 | 20/21 (95%) |
|  | dre‐mir‐190b | hsa‐mir‐190b | 34.2 | 3.00E‐06 | 20/21 (95%) |
|  | dre‐mir‐21 | hsa‐mir‐21 | 34.2 | 3.00E‐06 | 20/21 (95%) |
|  | dre‐mir‐216b | hsa‐mir‐216b | 34.2 | 3.00E‐06 | 20/21 (95%) |
|  | dre‐mir‐7b | hsa‐mir‐7 | 34.2 | 3.00E‐06 | 20/21 (95%) |
|  | dre‐mir‐93 | hsa‐mir‐93 | 34.2 | 3.00E‐06 | 20/21 (95%) |
|  | dre‐mir‐16c | hsa‐mir‐16 | 32.2 | 1.00E‐05 | 19/20 (95%) |
|  | dre‐mir‐202 | hsa‐mir‐202 | 32.2 | 1.00E‐05 | 19/20 (95%) |
|  | dre‐mir‐429 | hsa‐mir‐429 | 32.2 | 1.00E‐05 | 16/16 (100%) |
|  | dre‐mir‐125a | hsa‐mir‐125a‐5p | 30.2 | 4.00E‐05 | 22/23 (95%) |
|  | dre‐mir‐212 | hsa‐mir‐132 | 30.2 | 4.00E‐05 | 18/19 (94%) |
|  | dre‐mir‐139 | hsa‐mir‐139‐5p | 28.2 | 1.00E‐04 | 17/18 (94%) |
|  | dre‐mir‐18c | hsa‐mir‐18b | 28.2 | 2.00E‐04 | 20/22 (90%) |
|  | dre‐mir‐194b | hsa‐mir‐194 | 28.2 | 2.00E‐04 | 20/22 (90%) |
