# Supplementary material for: Reverse Genetics Screen in Zebrafish Identifies a Role of miR-142a-3p in Vascular Development and Integrity
Source: PLoS One. 2012 Dec 21;7(12):e52588. doi: 10.1371/journal.pone.0052588 (PMC3528674; doi:10.1371/journal.pone.0052588)
Supplement: Table S2 — MiRNAs involved in vasculature development. (DOCX) [file pone.0052588.s007.docx]

**Supplementary Table S2**: MicroRNAs involved in vasculature development

| **S.No.** | **MicroRNA Name** | **Organism** | **Target gene(s)** | **Biological processes affected by the miR** |
| --- | --- | --- | --- | --- |
| 1 | miR 126 | Mouse  Zebrafish  Cell lines | Spred1 and PIK3R2 [1,2]  VCAM-1[3] | Regulates angiogenic signaling, vascular integrity and promotes blood vessel formation[4] |
| 2 | miR 92a | Mouse | ITGA5 [5] | Overexpression inhibits neovascularization and sprout formation |
| 3 | miR 15 | Cell lines | VEGF (15b,16,20b,20a)  uPAR(15b, 16),  COX2(15b,16,20b)  c-MET(15b,16,20b,20a) [6] | Regulate expression of VEGF and other angiogenic factors |
| 4 | miR 16 | Cell lines | VEGF (15b,16,20b,20a)  uPAR(15b, 16),  COX2(15b,16,20b)  c-MET(15b,16,20b,20a) [6] | Regulate expression of VEGF and other angiogenic factors |
| 5 | miR 20a | Cell lines | VEGF (15b,16,20b,20a)  c-MET(15b,16,20b,20a) [6] | Regulate expression of VEGF and other angiogenic factors |
| 6 | miR 20b | Cell lines | VEGF (15b,16,20b,20a)  COX2(15b,16,20b)  c-MET(15b,16,20b,20a) [6] | Regulate expression of VEGF and other angiogenic factors |
| 7 | miR 24 | Cell lines | ALK4 [7] | Expression delays maturation of hematopoietic progenitor cells |
| 8 | miR 181a | Mouse | Prox1 [8] | re-programming of lymphatic endothelial cells  towards a blood vascular phenotype |
| 9 | miR 181b | Human | TCL1 [9] | B-cell chronic lymphocytic leukemia (B-CLL) |
| 10 | miR 181c | cell lines and mouse thymocytes | NA | express in BM CD11b^+^, Gr-1^+^, and B220^+^ cells and thymocytes [10] |
| 11 | miR 1 | Mice  Rat | Hand 2 [11]  Irx5 [12]  HDAC4 [13] | Inhibit myocardin induced contractility of human vascular smooth muscle cells [14]. Expression negatively regulates cardiac growth and promotes myoblast differentiation, apoptosis |
| 12 | miR 15a | Cell lines | c-Myb [15] | Overexpression blocks cells in G1 phase of cell cycle and inhibits erythroid differentiation |
| 13 | miR 27b | Cell lines | ND | Inhibition reduces angiogenic sprouting [16] |
| 14 | miR let 7f | Cell lines | ND | Inhibition reduces angiogenic sprouting [16] |
| 15 | miR 17-92 | Mouse | TSP1(miR-19)  and CTGF(miR-18) [17] | Overexpression promotes cell proliferation, survival and better perfused tumors in vivo |
| 16 | miR 17-92 | Mouse | E2F1[18] (17-5p, 20a),  PTEN and Bim[19] | Overexpression promotes cell proliferation, survival and better perfused tumors in vivo |
| 17 | miR 92a | Mouse | ITGA5 [5] | Overexpression inhibits neovascularization and sprout formation |
| 18 | miR 130a | Cell lines | GAX and HoxA5 [20] | Positive regulator for the angiogenic phenotype in Endothelial Cells (ECs) |
| 19 | miR 133 | Mouse  Rat | Cyclin D2 [21]  SRF [13] | Expression stimulates myoblast proliferation and prevents apoptosis |
| 20 | miR 138 | Zebrafish | aldh1a2 and cspg 2 [22] | Inhibition causes immature ventricular cardio- myocytes |
| 21 | miR 142 | Cell lines [23] | ND | ND |
| 22 | miR 143 | Mouse | Elk-1 [24] | Functions co-operatively with miR-145 to repress Vascular Smooth Muscle Cells (VSMC) proliferation |
| 23 | miR 145 | Mouse  Zebrafish | Klf4 and CamkIIδ [24]  gata 6 [25] | Promotes VSMC differentiation and intestinal maturation. Inhibition causes defective heart and gut development |
| 24 | miR 144 | Zebrafish | Klfd [26] | Regulates embryonic α-globin synthesis |
| 25 | miR 150 | Cell lines | Myb [27] | Regulates Megakaryocyte-Erythrocyte Progenitors (MEPs) fate decision |
| 26 | miR 155 | Cell lines | AT1R [28,29] | Inhibition stimulates Ang II mediated ERK1/2 expression |
| 27 | miR 206 | Mouse  Cell lines | Pola1 [30], cnx 43 [31]  Utrn and Fstl1 [32] | Promotes myogenesis of C2C12 myoblasts |
| 28 | miR 208 | mouse | THRAP 1(PREDICTED) [33] | Regulates fetal adult myosin isoform switching |
| 29 | miR 210 | Cell lines | Ephrin A3 [34] | Upregulation stimulates tubulogenesis and VEGF driven migration |
| 30 | miR 221 | CELL LINES  Rat | c-Kit [35]  p27(Kip1) and p57(Kip2) [36] | Suppress erythropoiesis and erythroleukemic cell growth |
| 31 | miR 222 | CELL LINES  Rat | c-Kit [35]  p27(Kip1) and p57(Kip2) [36] | Suppress erythropoiesis and erythroleukemic cell growth |
| 32 | miR 223 | Cell lines | LMO2 [37] | Downregulation is necessary for erythroid differentiation |
| 33 | miR 296 | Cell lines | HGS [38] | Expression promotes vascularization of tumor xenografts |
| 34 | miR 378 | Cell lines | Sufu and Fus 1b [39] | Expression promotes cell survival and angiogenesis |
| 35 | miR 451 | Zebrafish  Cell lines | gata2 [40] | Facilitates erythrocyte maturation |

Reference List

1. Fish JE, Santoro MM, Morton SU, Yu S, Yeh RF et al. (2008) miR-126 regulates angiogenic signaling and vascular integrity. Dev Cell 15: 272-284.

2. Kuhnert F, Mancuso MR, Hampton J, Stankunas K, Asano T et al. (2008) Attribution of vascular phenotypes of the murine Egfl7 locus to the microRNA miR-126. Development 135: 3989-3993.

3. Harris TA, Yamakuchi M, Ferlito M, Mendell JT, Lowenstein CJ (2008) MicroRNA-126 regulates endothelial expression of vascular cell adhesion molecule 1. Proc Natl Acad Sci U S A 105: 1516-1521.

4. Wang S, Aurora AB, Johnson BA, Qi X, McAnally J et al. (2008) The endothelial-specific microRNA miR-126 governs vascular integrity and angiogenesis. Dev Cell 15: 261-271.

5. Bonauer A, Carmona G, Iwasaki M, Mione M, Koyanagi M et al. (2009) MicroRNA-92a controls angiogenesis and functional recovery of ischemic tissues in mice. Science 324: 1710-1713.

6. Hua Z, Lv Q, Ye W, Wong CK, Cai G et al. (2006) MiRNA-directed regulation of VEGF and other angiogenic factors under hypoxia. PLoS One 1: e116.

7. Wang Q, Huang Z, Xue H, Jin C, Ju XL et al. (2008) MicroRNA miR-24 inhibits erythropoiesis by targeting activin type I receptor ALK4. Blood 111: 588-595.

8. Kazenwadel J, Michael MZ, Harvey NL (2010) Prox1 expression is negatively regulated by miR-181 in endothelial cells. Blood 116: 2395-2401.

9. Pekarsky Y, Santanam U, Cimmino A, Palamarchuk A, Efanov A et al. (2006) Tcl1 expression in chronic lymphocytic leukemia is regulated by miR-29 and miR-181. Cancer Res 66: 11590-11593.

10. Papapetrou EP, Kovalovsky D, Beloeil L, Sant'angelo D, Sadelain M (2009) Harnessing endogenous miR-181a to segregate transgenic antigen receptor expression in developing versus post-thymic T cells in murine hematopoietic chimeras. J Clin Invest 119: 157-168.

11. Zhao Y, Samal E, Srivastava D (2005) Serum response factor regulates a muscle-specific microRNA that targets Hand2 during cardiogenesis. Nature 436: 214-220.

12. Zhao Y, Ransom JF, Li A, Vedantham V, von DM et al. (2007) Dysregulation of cardiogenesis, cardiac conduction, and cell cycle in mice lacking miRNA-1-2. Cell 129: 303-317.

13. Chen JF, Mandel EM, Thomson JM, Wu Q, Callis TE et al. (2006) The role of microRNA-1 and microRNA-133 in skeletal muscle proliferation and differentiation. Nat Genet 38: 228-233.

14. Jiang Y, Yin H, Zheng XL (2010) MicroRNA-1 inhibits myocardin-induced contractility of human vascular smooth muscle cells. J Cell Physiol 225: 506-511.

15. Zhao H, Kalota A, Jin S, Gewirtz AM (2009) The c-myb proto-oncogene and microRNA-15a comprise an active autoregulatory feedback loop in human hematopoietic cells. Blood 113: 505-516.

16. Kuehbacher A, Urbich C, Zeiher AM, Dimmeler S (2007) Role of Dicer and Drosha for endothelial microRNA expression and angiogenesis. Circ Res 101: 59-68.

17. Dews M, Homayouni A, Yu D, Murphy D, Sevignani C et al. (2006) Augmentation of tumor angiogenesis by a Myc-activated microRNA cluster. Nat Genet 38: 1060-1065.

18. O'Donnell KA, Wentzel EA, Zeller KI, Dang CV, Mendell JT (2005) c-Myc-regulated microRNAs modulate E2F1 expression. Nature 435: 839-843.

19. Xiao C, Srinivasan L, Calado DP, Patterson HC, Zhang B et al. (2008) Lymphoproliferative disease and autoimmunity in mice with increased miR-17-92 expression in lymphocytes. Nat Immunol 9: 405-414.

20. Chen Y, Gorski DH (2008) Regulation of angiogenesis through a microRNA (miR-130a) that down-regulates antiangiogenic homeobox genes GAX and HOXA5. Blood 111: 1217-1226.

21. Liu N, Bezprozvannaya S, Williams AH, Qi X, Richardson JA et al. (2008) microRNA-133a regulates cardiomyocyte proliferation and suppresses smooth muscle gene expression in the heart. Genes Dev 22: 3242-3254.

22. Morton SU, Scherz PJ, Cordes KR, Ivey KN, Stainier DY et al. (2008) microRNA-138 modulates cardiac patterning during embryonic development. Proc Natl Acad Sci U S A 105: 17830-17835.

23. Merkerova M, Belickova M, Bruchova H (2008) Differential expression of microRNAs in hematopoietic cell lineages. Eur J Haematol 81: 304-310.

24. Cordes KR, Sheehy NT, White MP, Berry EC, Morton SU et al. (2009) miR-145 and miR-143 regulate smooth muscle cell fate and plasticity. Nature 460: 705-710.

25. Zeng L, Carter AD, Childs SJ (2009) miR-145 directs intestinal maturation in zebrafish. Proc Natl Acad Sci U S A 106: 17793-17798.

26. Fu YF, Du TT, Dong M, Zhu KY, Jing CB et al. (2009) Mir-144 selectively regulates embryonic alpha-hemoglobin synthesis during primitive erythropoiesis. Blood 113: 1340-1349.

27. Lu J, Guo S, Ebert BL, Zhang H, Peng X et al. (2008) MicroRNA-mediated control of cell fate in megakaryocyte-erythrocyte progenitors. Dev Cell 14: 843-853.

28. Martin MM, Lee EJ, Buckenberger JA, Schmittgen TD, Elton TS (2006) MicroRNA-155 regulates human angiotensin II type 1 receptor expression in fibroblasts. J Biol Chem 281: 18277-18284.

29. Martin MM, Buckenberger JA, Jiang J, Malana GE, Nuovo GJ et al. (2007) The human angiotensin II type 1 receptor +1166 A/C polymorphism attenuates microrna-155 binding. J Biol Chem 282: 24262-24269.

30. Kim HK, Lee YS, Sivaprasad U, Malhotra A, Dutta A (2006) Muscle-specific microRNA miR-206 promotes muscle differentiation. J Cell Biol 174: 677-687.

31. Anderson C, Catoe H, Werner R (2006) MIR-206 regulates connexin43 expression during skeletal muscle development. Nucleic Acids Res 34: 5863-5871.

32. Rosenberg MI, Georges SA, Asawachaicharn A, Analau E, Tapscott SJ (2006) MyoD inhibits Fstl1 and Utrn expression by inducing transcription of miR-206. J Cell Biol 175: 77-85.

33. van RE, Sutherland LB, Qi X, Richardson JA, Hill J et al. (2007) Control of stress-dependent cardiac growth and gene expression by a microRNA. Science 316: 575-579.

34. Fasanaro P, D'Alessandra Y, Di S, V, Melchionna R, Romani S et al. (2008) MicroRNA-210 modulates endothelial cell response to hypoxia and inhibits the receptor tyrosine kinase ligand Ephrin-A3. J Biol Chem 283: 15878-15883.

35. Poliseno L, Tuccoli A, Mariani L, Evangelista M, Citti L et al. (2006) MicroRNAs modulate the angiogenic properties of HUVECs. Blood 108: 3068-3071.

36. Liu X, Cheng Y, Zhang S, Lin Y, Yang J et al. (2009) A necessary role of miR-221 and miR-222 in vascular smooth muscle cell proliferation and neointimal hyperplasia. Circ Res 104: 476-487.

37. Felli N, Pedini F, Romania P, Biffoni M, Morsilli O et al. (2009) MicroRNA 223-dependent expression of LMO2 regulates normal erythropoiesis. Haematologica 94: 479-486.

38. Wurdinger T, Tannous BA, Saydam O, Skog J, Grau S et al. (2008) miR-296 regulates growth factor receptor overexpression in angiogenic endothelial cells. Cancer Cell 14: 382-393.

39. Lee DY, Deng Z, Wang CH, Yang BB (2007) MicroRNA-378 promotes cell survival, tumor growth, and angiogenesis by targeting SuFu and Fus-1 expression. Proc Natl Acad Sci U S A 104: 20350-20355.

40. Zhan M, Miller CP, Papayannopoulou T, Stamatoyannopoulos G, Song CZ (2007) MicroRNA expression dynamics during murine and human erythroid differentiation. Exp Hematol 35: 1015-1025.
