# Supplementary material for: Reverse Genetics Screen in Zebrafish Identifies a Role of miR-142a-3p in Vascular Development and Integrity
Source: PLoS One. 2012 Dec 21;7(12):e52588. doi: 10.1371/journal.pone.0052588 (PMC3528674; doi:10.1371/journal.pone.0052588)
Supplement: Table S3 — A dataset of 672 zebrafish genes with putative expression in blood and blood vessel tissue (DOC) [file pone.0052588.s008.doc]

**Supplementary Table S3**: A dataset of 672 zebrafish genes with putative expression in blood and blood vessel tissue

| S.no. | Gene | Gene Name | Zfin ID |
| --- | --- | --- | --- |
| 1 | *abat* | *4-aminobutyrate aminotransferase* | ZDB-GENE-031006-4 |
| 2 | *abca1a* | *ATP-binding cassette, sub-family A (ABC1), member 1A* | ZDB-GENE-031006-12 |
| 3 | *abce1* | *ATP-binding cassette, sub-family E (OABP), member 1* | ZDB-GENE-040426-1995 |
| 4 | *aco1* | *aconitase 1, soluble* | ZDB-GENE-031118-76 |
| 5 | *acss1* | *acyl-CoA synthetase short-chain family member 1* | ZDB-GENE-050320-139 |
| 6 | *adam8a* | *a disintegrin and metalloproteinase domain 8a* | ZDB-GENE-030616-622 |
| 7 | *adka* | *adenosine kinase a* | ZDB-GENE-030425-3 |
| 8 | *adora2aa* | *adenosine A2a receptor a* | ZDB-GENE-080723-28 |
| 9 | *adora2b* | *adenosine A2b receptor* | ZDB-GENE-060511-1 |
| 10 | *agfg1a* | *ArfGAP with FG repeats 1a* | ZDB-GENE-030131-5808 |
| 11 | *ahr2* | *aryl hydrocarbon receptor 2* | ZDB-GENE-990714-16 |
| 12 | *ak3* | *adenylate kinase 3* | ZDB-GENE-040426-2142 |
| 13 | *akap12* | *A kinase (PRKA) anchor protein (gravin) 12b* | ZDB-GENE-030131-9753 |
| 14 | *alas2* | *aminolevulinate, delta-, synthetase 2* | ZDB-GENE-001229-1 |
| 15 | *aldh16a1* | *aldehyde dehydrogenase 16 family, member A1* | ZDB-GENE-070112-2062 |
| 16 | *alp* | *alkaline phosphatase* | ZDB-GENE-040420-1 |
| 17 | *amotl2* | *angiomotin like 2a* | ZDB-GENE-030131-9770 |
| 18 | *ampd3* | *adenosine monophosphate deaminase 3* | ZDB-GENE-030131-5929 |
| 19 | *anp32b* | *acidic (leucine-rich) nuclear phosphoprotein 32 family, member B* | ZDB-GENE-030131-719 |
| 20 | *antxr1* | *anthrax toxin receptor 1* | ZDB-GENE-090514-5 |
| 21 | *anxa11a* | *annexin A11a* | ZDB-GENE-030707-4 |
| 22 | *ap2m1a* | *adaptor-related protein complex 2, mu 1 subunit, a* | ZDB-GENE-030131-9784 |
| 23 | *ap3b1* | *adaptor-related protein complex 3, beta 1 subunit* | ZDB-GENE-031112-8 |
| 24 | *aplnra* | *apelin receptor a* | ZDB-GENE-060929-512 |
| 25 | *aplnrb* | *apelin receptor b* | ZDB-GENE-050913-90 |
| 26 | *apoeb* | *apolipoprotein Eb* | ZDB-GENE-980526-368 |
| 27 | *appb* | *amyloid beta (A4) precursor protein b* | ZDB-GENE-020220-1 |
| 28 | *aqp8a* | *aquaporin 8a, tandem duplicate 1* | ZDB-GENE-040912-106 |
| 29 | *arhgap29a* | *Rho GTPase activating protein 29a* | ZDB-GENE-030131-9510 |
| 30 | *arhgef1* | *Rho guanine nucleotide exchange factor (GEF) 1a* | ZDB-GENE-030722-5 |
| 31 | *arhgef7b* | *Rho guanine nucleotide exchange factor (GEF) 7b* | ZDB-GENE-041212-49 |
| 32 | *arl4a* | *ADP-ribosylation factor-like 4aa* | ZDB-GENE-040426-1878 |
| 33 | *arl4l* | *ADP-ribosylation factor-like 4ab* | ZDB-GENE-030219-167 |
| 34 | *arnt2* | *aryl hydrocarbon receptor nuclear translocator 2* | ZDB-GENE-001207-3 |
| 35 | *arpc1b* | *actin related protein 2/3 complex, subunit 1B* | ZDB-GENE-030131-7414 |
| 36 | *arrb2b* | *arrestin, beta 2b* | ZDB-GENE-040426-1332 |
| 37 | *arrdc2* | *arrestin domain containing 2* | ZDB-GENE-030131-7146 |
| 38 | *aspn* | *asporin (LRR class 1)* | ZDB-GENE-041105-7 |
| 39 | *atp1a1* | *ATPase, Na+/K+ transporting, alpha 1 polypeptide* | ZDB-GENE-001212-1 |
| 40 | *atp1b1a* | *ATPase, Na+/K+ transporting, beta 1a polypeptide* | ZDB-GENE-001127-3 |
| 41 | *atp2b1a* | *ATPase, Ca++ transporting, plasma membrane 1a* | ZDB-GENE-030925-29 |
| 42 | *atp2b1b* | *ATPase, Ca++ transporting, plasma membrane 1b* | ZDB-GENE-080409-1 |
| 43 | *atp2b2* | *ATPase, Ca++ transporting, plasma membrane 2* | ZDB-GENE-061016-1 |
| 44 | *atp2b3a* | *ATPase, Ca++ transporting, plasma membrane 3a* | ZDB-GENE-040718-174 |
| 45 | *atp2b4* | *ATPase, Ca++ transporting, plasma membrane 4* | ZDB-GENE-061027-60 |
| 46 | *atp6v0a1* | *ATPase, H+ transporting, lysosomal V0 subunit a isoform 1a* | ZDB-GENE-030131-3027 |
| 47 | *atp6v0c* | *ATPase, H+ transporting, lysosomal, V0 subunit c, a* | ZDB-GENE-020419-23 |
| 48 | *atp6v1d* | *ATPase, H+ transporting, V1 subunit D* | ZDB-GENE-040426-727 |
| 49 | *ba1* | *ba1 globin* | ZDB-GENE-990415-18 |
| 50 | *ba2l* | *ba2 globin, like* | ZDB-GENE-040901-3 |
| 51 | *bactin1* | *actin, beta 1* | ZDB-GENE-000329-1 |
| 52 | *baxa* | *bcl2-associated X protein, a* | ZDB-GENE-000511-6 |
| 53 | *bcas2* | *breast carcinoma amplified sequence 2* | ZDB-GENE-030408-5 |
| 54 | *bcl2* | *B-cell leukemia/lymphoma 2* | ZDB-GENE-051012-1 |
| 55 | *bcl2l* | *bcl2-like 1* | ZDB-GENE-010730-1 |
| 56 | *bhlhe40* | *basic helix-loop-helix family, member e40* | ZDB-GENE-030131-3133 |
| 57 | *birc2* | *baculoviral IAP repeat-containing 2* | ZDB-GENE-030825-6 |
| 58 | *birc5a* | *baculoviral IAP repeat-containing 5a* | ZDB-GENE-030826-1 |
| 59 | *birc5b* | *baculoviral IAP repeat-containing 5B* | ZDB-GENE-030826-2 |
| 60 | *blf* | *bloody fingers* | ZDB-GENE-050721-1 |
| 61 | *blvrb* | *biliverdin reductase B (flavin reductase (NADPH)* | ZDB-GENE-030131-1516 |
| 62 | *bmp2b* | *bone morphogenetic protein 2b* | ZDB-GENE-980526-474 |
| 63 | *bmp4* | *bone morphogenetic protein 4* | ZDB-GENE-980528-2059 |
| 64 | *bmper* | *BMP binding endothelial regulator* | ZDB-GENE-030219-146 |
| 65 | *bmpr2a* | *bone morphogenetic protein receptor, type II a (serine/threonine kinase)* | ZDB-GENE-070618-1 |
| 66 | *bmpr2b* | bone morphogenetic protein receptor, type II b (serine/threonine kinase) | ZDB-GENE-070618-2 |
| 67 | *brd2a* | *bromodomain-containing 2a* | ZDB-GENE-990415-248 |
| 68 | *bty* | *bloodthirsty* | ZDB-GENE-020620-2 |
| 69 | *bxdc1* | *ribosome production factor 2 homolog (S. cerevisiae)* | ZDB-GENE-040426-2501 |
| 70 | *c10orf119* | *minichromosome maintenance complex binding protein* | ZDB-GENE-030131-9676 |
| 71 | *ca10a* | *carbonic anhydrase Xa* | ZDB-GENE-051030-123 |
| 72 | *ca2* | *carbonic anhydrase II* | ZDB-GENE-031219-5 |
| 73 | *ca5* | *carbonic anhydrase V* | ZDB-GENE-080220-57 |
| 74 | *ca6* | *carbonic anhydrase VI* | ZDB-GENE-030131-7091 |
| 75 | *cahz* | *carbonic anhydrase* | ZDB-GENE-980526-39 |
| 76 | *calcrla* | *calcitonin receptor-like a* | ZDB-GENE-040822-26 |
| 77 | *cald1* | *caldesmon 1* | ZDB-GENE-090313-229 |
| 78 | *calm1a* | *calmodulin 1a* | ZDB-GENE-030131-8308 |
| 79 | *calr* | *calreticulin* | ZDB-GENE-000208-17 |
| 80 | *calub* | *calumenin b* | ZDB-GENE-040426-1251 |
| 81 | *cav1* | *caveolin 1* | ZDB-GENE-030131-2415 |
| 82 | *cbfb* | *core-binding factor, beta subunit* | ZDB-GENE-980526-440 |
| 83 | *ccm2* | *cerebral cavernous malformation 2* | ZDB-GENE-040712-6 |
| 84 | *ccnd1* | *cyclin D1* | ZDB-GENE-980526-176 |
| 85 | *cd247* | *CD247 antigen* | ZDB-GENE-061130-4 |
| 86 | *cd247l* | *CD247 antigen like* | ZDB-GENE-070508-2 |
| 87 | *cd8a* | *CD8 antigen, alpha polypeptide* | ZDB-GENE-060210-2 |
| 88 | *cdc42ep1* | *CDC42 effector protein (Rho GTPase binding) 1* | ZDB-GENE-030131-5813 |
| 89 | *cdh17* | *cadherin 17, LI cadherin (liver-intestine)* | ZDB-GENE-030910-3 |
| 90 | *cdh5* | *cadherin 5* | ZDB-GENE-040816-1 |
| 91 | *cdx4* | *caudal type homeo box transcription factor 4* | ZDB-GENE-980526-330 |
| 92 | *cebp1* | *CCAAT/enhancer binding protein (C/EBP) 1* | ZDB-GENE-010611-1 |
| 93 | *cebpa* | *CCAAT/enhancer binding protein (C/EBP), alpha* | ZDB-GENE-020111-2 |
| 94 | *cebpb* | *CCAAT/enhancer binding protein (C/EBP), beta* | ZDB-GENE-020111-3 |
| 95 | *cldng* | *claudin g* | ZDB-GENE-010328-7 |
| 96 | *clec14a* | *C-type lectin domain family 14, member A* | ZDB-GENE-030131-5536 |
| 97 | *clgn* | *calmegin* | ZDB-GENE-060929-708 |
| 98 | *cltca* | *clathrin, heavy polypeptide a (Hc)* | ZDB-GENE-030131-2299 |
| 99 | *cmyb* | *transcription factor cmyb* | ZDB-GENE-991110-14 |
| 100 | *cndp2* | *CNDP dipeptidase 2 (metallopeptidase M20 family)* | ZDB-GENE-030131-5499 |
| 101 | *cnn2* | *calponin 2* | ZDB-GENE-030131-542 |
| 102 | *cnn3a* | *calponin 3, acidic a* | ZDB-GENE-030131-5130 |
| 103 | *col12a1* | *collagen, type XII, alpha 1* | ZDB-GENE-090728-1 |
| 104 | *copb2* | *coatomer protein complex, subunit beta 2* | ZDB-GENE-010724-7 |
| 105 | *coro1a* | *coronin, actin binding protein, 1A* | ZDB-GENE-030131-9512 |
| 106 | *cotl1* | *coactosin-like 1 (Dictyostelium)* | ZDB-GENE-030131-8325 |
| 107 | *cox17* | *COX17 cytochrome c oxidase assembly homolog (S. cerevisiae)* | ZDB-GENE-040912-91 |
| 108 | *cpa5* | *carboxypeptidase A5* | ZDB-GENE-020514-1 |
| 109 | *cpn1* | *carboxypeptidase N, polypeptide 1* | ZDB-GENE-030131-9116 |
| 110 | *cpne1* | *copine I* | ZDB-GENE-030131-3562 |
| 111 | *cpox* | *coproporphyrinogen oxidase* | ZDB-GENE-030131-9884 |
| 112 | *crim1* | *cysteine rich transmembrane BMP regulator 1 (chordin like)* | ZDB-GENE-040312-2 |
| 113 | *crip2* | *cysteine-rich protein 2* | ZDB-GENE-040426-2889 |
| 114 | *crlf3* | *cytokine receptor-like factor 3* | ZDB-GENE-050417-354 |
| 115 | *csf1r* | *colony stimulating factor 1 receptor, a* | ZDB-GENE-001205-1 |
| 116 | *csrp1* | *cysteine and glycine-rich protein 1a* | ZDB-GENE-030909-4 |
| 117 | *ctsba* | *cathepsin B, a* | ZDB-GENE-040426-2650 |
| 118 | *ctsc* | *cathepsin C* | ZDB-GENE-030619-9 |
| 119 | *ctsl1a* | *cathepsin L, 1 a* | ZDB-GENE-030131-106 |
| 120 | *ctssb.2* | *cathepsin S, b.2* | ZDB-GENE-050626-55 |
| 121 | *ctssl* | *cathepsin S, like* | ZDB-GENE-021031-6 |
| 122 | *ctsz* | *cathepsin Z* | ZDB-GENE-041010-139 |
| 123 | *cwf19l1* | *CWF19-like 1, cell cycle control* | ZDB-GENE-031204-5 |
| 124 | *cx43* | *connexin 43* | ZDB-GENE-991105-4 |
| 125 | *cxcl12b* | *chemokine (C-X-C motif) ligand 12b (stromal cell-derived factor 1)* | ZDB-GENE-030721-1 |
| 126 | *cxcr3.2* | *chemokine (C-X-C motif) receptor 3.2* | ZDB-GENE-041114-186 |
| 127 | *cxcr4a* | *chemokine (C-X-C motif) receptor 4a* | ZDB-GENE-020102-1 |
| 128 | *cyb5r4* | *cytochrome b5 reductase 4* | ZDB-GENE-050522-225 |
| 129 | *cybb* | *cytochrome b-245, beta polypeptide (chronic granulomatous disease)* | ZDB-GENE-040426-1380 |
| 130 | *cyp1a* | *cytochrome P450, family 1, subfamily A* | ZDB-GENE-011219-1 |
| 131 | *dab2* | *disabled homolog 2 (Drosophila)* | ZDB-GENE-040303-1 |
| 132 | *dact2* | *dapper homolog 2, antagonist of beta-catenin (xenopus)* | ZDB-GENE-030131-9975 |
| 133 | *dera* | *2-deoxyribose-5-phosphate aldolase homolog (C. elegans)* | ZDB-GENE-041010-200 |
| 134 | *dfna5* | *deafness, autosomal dominant 5* | ZDB-GENE-030131-7662 |
| 135 | *dhfr* | *dihydrofolate reductase* | ZDB-GENE-010406-5 |
| 136 | *dhrs3b* | *dehydrogenase/reductase (SDR family) member 3b* | ZDB-GENE-041010-172 |
| 137 | *dlc* | *deltaC* | ZDB-GENE-000125-4 |
| 138 | *dll4* | *delta-like 4 (Drosophila)* | ZDB-GENE-041014-73 |
| 139 | *dnajb11* | *DnaJ (Hsp40) homolog, subfamily B, member 11* | ZDB-GENE-031113-9 |
| 140 | *dnajc3* | *DnaJ (Hsp40) homolog, subfamily C, member 3* | ZDB-GENE-030131-1264 |
| 141 | *dnmt4* | *DNA (cytosine-5-)-methyltransferase 4* | ZDB-GENE-050314-1 |
| 142 | *dock2* | *dedicator of cyto-kinesis 2* | ZDB-GENE-031125-1 |
| 143 | *drl* | *draculin* | ZDB-GENE-991213-3 |
| 144 | *dub* | *duboraya* | ZDB-GENE-060825-81 |
| 145 | *dusp1* | *dual specificity phosphatase 1* | ZDB-GENE-040426-2018 |
| 146 | *dusp5* | *dual specificity phosphatase 5* | ZDB-GENE-010625-1 |
| 147 | *efnb2a* | *ephrin B2a* | ZDB-GENE-990415-67 |
| 148 | *elmo1* | *engulfment and cell motility 1 (ced-12 homolog, C. elegans)* | ZDB-GENE-040426-2069 |
| 149 | *elna* | *elastin a* | ZDB-GENE-061212-1 |
| 150 | *elnb* | *elastin b* | ZDB-GENE-061212-2 |
| 151 | *elovl1a* | *elongation of very long chain fatty acids (FEN1/Elo2, SUR4/Elo3, yeast)-like 1a* | ZDB-GENE-041010-66 |
| 152 | *elovl1b* | *elongation of very long chain fatty acids (FEN1/Elo2, SUR4/Elo3, yeast)-like 1b* | ZDB-GENE-040426-2755 |
| 153 | *elovl5* | *ELOVL family member 5, elongation of long chain fatty acids (yeast)* | ZDB-GENE-040407-2 |
| 154 | *emilin1a* | *elastin microfibril interfacer 1a* | ZDB-GENE-041001-191 |
| 155 | *emilin2a* | *elastin microfibril interfacer 2a* | ZDB-GENE-060503-247 |
| 156 | *emilin2b* | *elastin microfibril interfacer 2b* | ZDB-GENE-080416-1 |
| 157 | *entpd1* | *ectonucleoside triphosphate diphosphohydrolase 1* | ZDB-GENE-040801-58 |
| 158 | *eomesa* | *eomesodermin homolog a* | ZDB-GENE-001228-1 |
| 159 | *eomesb* | *eomesodermin homolog b* | ZDB-GENE-070318-1 |
| 160 | *epas1* | *endothelial PAS domain protein 1b* | ZDB-GENE-060607-11 |
| 161 | *epb41* | *erythrocyte membrane protein band 4.1 (elliptocytosis 1, RH-linked)* | ZDB-GENE-030130-1 |
| 162 | *ephb4a* | *eph receptor B4a* | ZDB-GENE-990415-62 |
| 163 | *epor* | *erythropoietin receptor* | ZDB-GENE-071116-1 |
| 164 | *eppk1* | *epiplakin 1* | ZDB-GENE-030416-1 |
| 165 | *erg* | *v-ets erythroblastosis virus E26 oncogene like (avian)* | ZDB-GENE-041212-40 |
| 166 | *ets1a* | *v-ets erythroblastosis virus E26 oncogene homolog 1a* | ZDB-GENE-021115-5 |
| 167 | *etv2* | *ets variant gene 2* | ZDB-GENE-050622-14 |
| 168 | *etv6* | *ets variant gene 6 (TEL oncogene)* | ZDB-GENE-010727-1 |
| 169 | *f10* | *coagulation factor X* | ZDB-GENE-021206-9 |
| 170 | *fabp11a* | *fatty acid binding protein 11a* | ZDB-GENE-040912-132 |
| 171 | *fam46c* | *family with sequence similarity 46, member C* | ZDB-GENE-030131-5365 |
| 172 | *fbn2b* | *fibrillin 2b* | ZDB-GENE-090112-3 |
| 173 | *fbxo5* | *F-box protein 5* | ZDB-GENE-030131-4027 |
| 174 | *fcer1g* | *Fc receptor, IgE, high affinity I, gamma polypeptide* | ZDB-GENE-061130-3 |
| 175 | *fcer1gl* | *Fc receptor, IgE, high affinity I, gamma polypeptide like* | ZDB-GENE-070502-4 |
| 176 | *fech* | *ferrochelatase* | ZDB-GENE-000928-1 |
| 177 | *fev* | *FEV (ETS oncogene family)* | ZDB-GENE-070112-1852 |
| 178 | *fgd5* | *FYVE, RhoGEF and PH domain containing 5* | ZDB-GENE-090428-3 |
| 179 | *fgf13l* | *fibroblast growth factor 13b* | ZDB-GENE-040426-1793 |
| 180 | *fgf19* | *fibroblast growth factor 19* | ZDB-GENE-030729-32 |
| 181 | *fgfrl1b* | *fibroblast growth factor receptor-like 1b* | ZDB-GENE-050201-3 |
| 182 | *fhl* | *four and a half LIM domains* | ZDB-GENE-031219-1 |
| 183 | *fkbp10* | *FK506 binding protein 10* | ZDB-GENE-030131-3101 |
| 184 | *fli1a* | *friend leukemia integration 1a* | ZDB-GENE-980526-426 |
| 185 | *fli1b* | *friend leukemia integration 1b* | ZDB-GENE-031114-3 |
| 186 | *flt1* | *fms-related tyrosine kinase 1 (vascular endothelial growth factor/vascular permeability factor receptor)* | ZDB-GENE-050407-1 |
| 187 | *flt4* | *fms-related tyrosine kinase 4* | ZDB-GENE-980526-326 |
| 188 | *fos* | *v-fos FBJ murine osteosarcoma viral oncogene homolog* | ZDB-GENE-031222-4 |
| 189 | *foxc1a* | *forkhead box C1a* | ZDB-GENE-010302-1 |
| 190 | *freqa* | *neuronal calcium sensor 1a* | ZDB-GENE-021220-1 |
| 191 | *frrs1* | *ferric-chelate reductase 1* | ZDB-GENE-080917-44 |
| 192 | *fstl1b* | *follistatin-like 1b* | ZDB-GENE-030131-3029 |
| 193 | *fth1* | *ferritin, heavy polypeptide 1a* | ZDB-GENE-000831-2 |
| 194 | *ftr82* | *finTRIM family, member 82* | ZDB-GENE-030219-207 |
| 195 | *fuca2* | *fucosidase, alpha-L- 2, plasma* | ZDB-GENE-040822-39 |
| 196 | *fzd2* | *frizzled homolog 2* | ZDB-GENE-990415-224 |
| 197 | *fzd7b* | *frizzled homolog 7b* | ZDB-GENE-990415-229 |
| 198 | *gapdhs* | *glyceraldehyde-3-phosphate dehydrogenase, spermatogenic* | ZDB-GENE-020913-1 |
| 199 | *gata1* | *GATA binding protein 1a* | ZDB-GENE-980526-268 |
| 200 | *gata2a* | *GATA-binding protein 2a* | ZDB-GENE-980526-260 |
| 201 | *gata3* | *GATA-binding protein 3* | ZDB-GENE-990415-82 |
| 202 | *gats* | *GATS protein-like 2* | ZDB-GENE-030131-5505 |
| 203 | *gb:bg305838* | *expressed sequence BG305838* | ZDB-GENE-061214-7 |
| 204 | *gb:bm036580* | *gb:bm036580* | ZDB-GENE-090429-4 |
| 205 | *gbx2* | *gastrulation brain homeo box 2* | ZDB-GENE-020509-2 |
| 206 | *gclc* | *glutamate-cysteine ligase, catalytic subunit* | ZDB-GENE-030131-5056 |
| 207 | *gcm2* | *glial cells missing homolog 2 (Drosophila)* | ZDB-GENE-050127-1 |
| 208 | *gdf6a* | *growth differentiation factor 6a* | ZDB-GENE-980526-373 |
| 209 | *gfap* | *glial fibrillary acidic protein* | ZDB-GENE-990914-3 |
| 210 | *gfi1.1* | *growth factor independent 1.1* | ZDB-GENE-050522-534 |
| 211 | *gipc1* | *GIPC PDZ domain containing family, member 1* | ZDB-GENE-060726-1 |
| 212 | *glrx5* | *glutaredoxin 5 homolog (S. cerevisiae)* | ZDB-GENE-040426-1957 |
| 213 | *gltscr1* | *glioma tumor suppressor candidate region gene 1* | ZDB-GENE-031116-2 |
| 214 | *glud1a* | *glutamate dehydrogenase 1a* | ZDB-GENE-030114-2 |
| 215 | *glula* | *glutamate-ammonia ligase (glutamine synthase) a* | ZDB-GENE-030131-688 |
| 216 | *glulb* | *glutamate-ammonia ligase (glutamine synthase) b* | ZDB-GENE-030131-8417 |
| 217 | *gnai2l* | *guanine nucleotide binding protein (G protein), alpha inhibiting activity polypeptide 2, like* | ZDB-GENE-030131-8365 |
| 218 | *gng2* | *guanine nucleotide binding protein (G protein), gamma 2* | ZDB-GENE-050417-59 |
| 219 | *gpd1b* | *glycerol-3-phosphate dehydrogenase 1b* | ZDB-GENE-030131-3906 |
| 220 | *gpr182* | *G protein-coupled receptor 182* | ZDB-GENE-030131-2361 |
| 221 | *grcfp* | *grcfp* | ZDB-EFG-081124-1 |
| 222 | *grna* | *granulin a* | ZDB-GENE-030131-8434 |
| 223 | *grnb* | *granulin b* | ZDB-GENE-030131-7393 |
| 224 | *gtpbp1* | *GTP binding protein 1* | ZDB-GENE-030909-12 |
| 225 | *gygl* | *glycogenin, like* | ZDB-GENE-040625-30 |
| 226 | *gypc* | *glycophorin C (Gerbich blood group)* | ZDB-GENE-030131-7930 |
| 227 | *hapln1b* | *hyaluronan and proteoglycan link protein 1b* | ZDB-GENE-050920-1 |
| 228 | *hapln3* | *hyaluronan and proteoglycan link protein 3* | ZDB-GENE-040426-2089 |
| 229 | *hbaa1* | *hemoglobin alpha adult-1* | ZDB-GENE-980526-79 |
| 230 | *hbae1* | *hemoglobin alpha embryonic-1* | ZDB-GENE-980526-80 |
| 231 | *hbae3* | *hemoglobin alpha embryonic-3* | ZDB-GENE-990706-3 |
| 232 | *hbbe1.1* | *hemoglobin beta embryonic-1.1* | ZDB-GENE-030616-7 |
| 233 | *hbbe2* | *hemoglobin beta embryonic-2* | ZDB-GENE-040702-1 |
| 234 | *hbbe3* | *hemoglobin beta embryonic-3* | ZDB-GENE-980526-287 |
| 235 | *hcst* | *hematopoietic cell signal transducer* | ZDB-GENE-061130-1 |
| 236 | *hdr* | *hematopoietic death receptor* | ZDB-GENE-030826-5 |
| 237 | *heg* | *heart of glass* | ZDB-GENE-040714-1 |
| 238 | *helb* | *helicase (DNA) B* | ZDB-GENE-030729-16 |
| 239 | *her4.1* | *hairy-related 4.1* | ZDB-GENE-980526-521 |
| 240 | *hey1* | *hairy/enhancer-of-split related with YRPW motif 1* | ZDB-GENE-000607-70 |
| 241 | *hey2* | *hairy/enhancer-of-split related with YRPW motif 2* | ZDB-GENE-000526-1 |
| 242 | *hhex* | *hematopoietically expressed homeobox* | ZDB-GENE-980526-299 |
| 243 | *hif1ab* | *hypoxia-inducible factor 1, alpha subunit (basic helix-loop-helix transcription factor) b* | ZDB-GENE-040426-706 |
| 244 | *hk2* | *hexokinase 2* | ZDB-GENE-040426-2017 |
| 245 | *hmbsb* | *hydroxymethylbilane synthase, b* | ZDB-GENE-050522-491 |
| 246 | *hmox1* | *heme oxygenase (decycling) 1* | ZDB-GENE-030131-3102 |
| 247 | *hoxc9a* | *homeo box C9a* | ZDB-GENE-000328-5 |
| 248 | *hsd3b7* | *hydroxy-delta-5-steroid dehydrogenase, 3 beta- and steroid delta-isomerase* | ZDB-GENE-030131-5673 |
| 249 | *hspa12b* | *heat shock protein 12B* | ZDB-GENE-070226-1 |
| 250 | *hspa5* | *heat shock protein 5* | ZDB-GENE-031001-11 |
| 251 | *hspa9* | *heat shock protein 9* | ZDB-GENE-030828-12 |
| 252 | *hspg2* | *heparan sulfate proteoglycan 2* | ZDB-GENE-080807-4 |
| 253 | *htt* | *huntingtin* | ZDB-GENE-990415-131 |
| 254 | *id:ibd2600* | id:ibd2600 | ZDB-GENE-010319-10 |
| 255 | *id:ibd5037* | id:ibd5037 | ZDB-GENE-000607-80 |
| 256 | *ifi30* | *interferon gamma inducible protein 30* | ZDB-GENE-030131-8447 |
| 257 | *igfbp1a* | *insulin-like growth factor binding protein 1a* | ZDB-GENE-021231-1 |
| 258 | *igfbp2a* | *insulin-like growth factor binding protein 2a* | ZDB-GENE-000125-12 |
| 259 | *ikzf1* | *IKAROS family zinc finger 1 (Ikaros)* | ZDB-GENE-980526-304 |
| 260 | *il13ra2* | *interleukin 13 receptor, alpha 2* | ZDB-GENE-030521-10 |
| 261 | *ildr2* | *immunoglobulin-like domain containing receptor 2* | ZDB-GENE-050706-83 |
| 262 | *ilk* | *integrin linked kinase* | ZDB-GENE-040426-1435 |
| 263 | *illr1* | *immune-related, lectin-like receptor 1* | ZDB-GENE-050311-2 |
| 264 | *illr3* | *immune-related, lectin-like receptor 3* | ZDB-GENE-050311-4 |
| 265 | *illr4* | *immune-related, lectin-like receptor 4* | ZDB-GENE-050311-5 |
| 266 | *im:4729603* | im:4729603 | ZDB-GENE-051220-2 |
| 267 | *im:5377142* | im:5377142 | ZDB-GENE-051220-3 |
| 268 | *im:5629217* | *mannose receptor, C type 1a* | ZDB-GENE-090915-4 |
| 269 | *im:6911889* | im:6911889 | ZDB-GENE-050208-14 |
| 270 | *im:7137402* | im:7137402 | ZDB-GENE-051214-1 |
| 271 | *im:7137497* | im:7137497 | ZDB-GENE-041008-105 |
| 272 | *im:7148949* | im:7148949 | ZDB-GENE-080225-5 |
| 273 | *im:7149055* | im:7149055 | ZDB-GENE-041111-256 |
| 274 | *im:7150531* | im:7150531 | ZDB-GENE-060810-42 |
| 275 | *im:7151244* | im:7151244 | ZDB-GENE-041111-278 |
| 276 | *im:7152141* | im:7152141 | ZDB-GENE-050309-217 |
| 277 | *im:7152557* | im:7152557 | ZDB-GENE-041111-311 |
| 278 | *im:7153558* | im:7153558 | ZDB-GENE-080225-12 |
| 279 | *im:7154036* | im:7154036 | ZDB-GENE-080917-11 |
| 280 | *im:7156501* | im:7156501 | ZDB-GENE-080225-21 |
| 281 | *im:7159770* | im:7159770 | ZDB-GENE-080917-38 |
| 282 | *inka1b* | *family with sequence similarity 212, member Ab* | ZDB-GENE-030131-5539 |
| 283 | *ipo9* | *importin 9* | ZDB-GENE-040426-2953 |
| 284 | *ireb2* | *iron-responsive element binding protein 2* | ZDB-GENE-051205-1 |
| 285 | *itga2b* | *integrin, alpha 2b (platelet glycoprotein IIb of IIb/IIIa complex, antigen CD41B)* | ZDB-GENE-051031-1 |
| 286 | *itga5* | *integrin, alpha 5 (fibronectin receptor, alpha polypeptide)* | ZDB-GENE-031116-52 |
| 287 | *itgav* | *integrin, alpha V* | ZDB-GENE-060616-382 |
| 288 | *itgb1b* | *integrin, beta 1b* | ZDB-GENE-030909-10 |
| 289 | *itgb3b* | *integrin beta 3b* | ZDB-GENE-071207-2 |
| 290 | *itm2bb* | *integral membrane protein 2Bb* | ZDB-GENE-040426-2139 |
| 291 | *jak1* | *Janus kinase 1* | ZDB-GENE-980526-142 |
| 292 | *jak2a* | *Janus kinase 2a* | ZDB-GENE-980526-481 |
| 293 | *jam2* | *junctional adhesion molecule 2a* | ZDB-GENE-031204-3 |
| 294 | *junb* | *jun B proto-oncogene a* | ZDB-GENE-040426-2172 |
| 295 | *kdr* | *kinase insert domain receptor (a type III receptor tyrosine kinase)* | ZDB-GENE-041001-112 |
| 296 | *kdrl* | *kinase insert domain receptor like* | ZDB-GENE-000705-1 |
| 297 | *kitlgb* | *kit ligand b* | ZDB-GENE-070424-2 |
| 298 | *klf4* | *Kruppel-like factor 4* | ZDB-GENE-010129-1 |
| 299 | *klfd* | *Kruppel-like factor d* | ZDB-GENE-980526-55 |
| 300 | *kpnb3* | *karyopherin (importin) beta 3* | ZDB-GENE-030424-2 |
| 301 | *krcp* | *kelch repeat-containing protein* | ZDB-GENE-030131-2126 |
| 302 | *krit1* | *KRIT1, ankyrin repeat containing* | ZDB-GENE-030131-555 |
| 303 | *krt18* | *keratin 18* | ZDB-GENE-030411-6 |
| 304 | *krt8* | *keratin 8* | ZDB-GENE-030411-5 |
| 305 | *lama4* | *laminin, alpha 4* | ZDB-GENE-040724-213 |
| 306 | *lamp2* | *lysosomal membrane glycoprotein 2* | ZDB-GENE-030729-9 |
| 307 | *lck* | *lymphocyte-specific protein tyrosine kinase* | ZDB-GENE-040617-1 |
| 308 | *lcp1* | *lymphocyte cytosolic plastin 1* | ZDB-GENE-991213-5 |
| 309 | *ldb2a* | *LIM-domain binding factor 2a* | ZDB-GENE-990415-136 |
| 310 | *lgals2a* | *lectin, galactoside-binding, soluble, 2a* | ZDB-GENE-050318-2 |
| 311 | *lgals2b* | *lectin, galactoside-binding, soluble, 2b* | ZDB-GENE-040426-1590 |
| 312 | *lgals3bpb* | *lectin, galactoside-binding, soluble, 3 binding protein b* | ZDB-GENE-040426-2262 |
| 313 | *lgals9l1* | *lectin, galactoside-binding, soluble, 9 (galectin 9)-like 1* | ZDB-GENE-030131-9543 |
| 314 | *lgmn* | *legumain* | ZDB-GENE-021030-1 |
| 315 | *lima1* | *LIM domain and actin binding 1* | ZDB-GENE-001120-1 |
| 316 | *limk1* | *LIM domain kinase 1* | ZDB-GENE-061212-3 |
| 317 | *limk2* | *LIM domain kinase 2* | ZDB-GENE-040718-398 |
| 318 | *llgl2* | *lethal giant larvae homolog 2 (Drosophila)* | ZDB-GENE-030131-9877 |
| 319 | *lmo2* | *LIM domain only 2 (rhombotin-like 1)* | ZDB-GENE-980526-419 |
| 320 | *lmo4* | *LIM domain only 4a* | ZDB-GENE-010702-1 |
| 321 | *lndc1* | *ly6/neurotoxin domain containing 1* | ZDB-GENE-030131-7871 |
| 322 | *lpar1* | *lysophosphatidic acid receptor 1* | ZDB-GENE-030616-499 |
| 323 | *lpp* | *LIM domain containing preferred translocation partner in lipoma* | ZDB-GENE-040426-918 |
| 324 | *lrrc15* | *leucine rich repeat containing 15* | ZDB-GENE-031113-8 |
| 325 | *lrrc33* | *leucine rich repeat containing 33* | ZDB-GENE-040808-36 |
| 326 | *lta4h* | *leukotriene A4 hydrolase* | ZDB-GENE-040426-247 |
| 327 | *lyve1l* | *lymphatic vessel endothelial hyaluronic acid receptor 1* | ZDB-GENE-030131-9516 |
| 328 | *lyz* | *lysozyme* | ZDB-GENE-020515-2 |
| 329 | *mafbb* | *v-maf musculoaponeurotic fibrosarcoma oncogene family, protein B, duplicate b* | ZDB-GENE-010605-4 |
| 330 | *mak10* | *N(alpha)-acetyltransferase 35, NatC auxiliary subunit* | ZDB-GENE-030131-306 |
| 331 | *map1lc3b* | *microtubule-associated protein 1 light chain 3 beta* | ZDB-GENE-030131-1145 |
| 332 | *mapk1* | *mitogen-activated protein kinase 1* | ZDB-GENE-030722-2 |
| 333 | *mb* | *myoglobin* | ZDB-GENE-040426-1430 |
| 334 | *mcam* | *melanoma cell adhesion molecule b* | ZDB-GENE-030219-213 |
| 335 | *mcm3* | *MCM3 minichromosome maintenance deficient 3 (S. cerevisiae)* | ZDB-GENE-020419-4 |
| 336 | *mdkb* | *midkine-related growth factor b* | ZDB-GENE-010131-6 |
| 337 | *mdm2* | *transformed 3T3 cell double minute 2 homolog (mouse)* | ZDB-GENE-990415-153 |
| 338 | *mfap2* | *microfibrillar-associated protein 2* | ZDB-GENE-030131-6710 |
| 339 | *micall2* | *mical-like 2a* | ZDB-GENE-030131-5409 |
| 340 | *mll5* | *myeloid/lymphoid or mixed-lineage leukemia 5 (trithorax homolog, Drosophila)* | ZDB-GENE-030131-4120 |
| 341 | *mmp13* | *matrix metalloproteinase 13a* | ZDB-GENE-031202-2 |
| 342 | *mmp2* | *matrix metalloproteinase 2* | ZDB-GENE-030131-9123 |
| 343 | *mmp9* | *matrix metalloproteinase 9* | ZDB-GENE-040426-2132 |
| 344 | *mmrn2a* | *multimerin 2a* | ZDB-GENE-080424-3 |
| 345 | *mpeg1* | *macrophage expressed 1* | ZDB-GENE-030131-7347 |
| 346 | *mpll* | *myeloproliferative leukemia virus oncogene* | ZDB-GENE-060421-1 |
| 347 | *mpp1* | *membrane protein, palmitoylated 1* | ZDB-GENE-031113-4 |
| 348 | *mpx* | *myeloid-specific peroxidase* | ZDB-GENE-030131-9460 |
| 349 | *ms4a17a.* |  |  |
| 350 | *mt* | *metallothionein* | ZDB-GENE-990415-159 |
| 351 | *mt2* | *metallothionein 2* | ZDB-GENE-030131-4174 |
| 352 | *mtmr8* | *myotubularin related protein 8* | ZDB-GENE-040426-1016 |
| 353 | *myb* | *myeloblastosis oncogene* | ZDB-GENE-980526-529 |
| 354 | *mybl1* | *v-myb myeloblastosis viral oncogene homolog (avian)-like 1* | ZDB-GENE-041111-281 |
| 355 | *myca* | *myelocytomatosis oncogene a* | ZDB-GENE-990415-162 |
| 356 | *mycb* | *myelocytomatosis oncogene b* | ZDB-GENE-040426-780 |
| 357 | *mych* | *myelocytomatosis oncogene homolog* | ZDB-GENE-030219-51 |
| 358 | *myct1* | *myc target 1* | ZDB-GENE-041001-143 |
| 359 | *nat13* | *N(alpha)-acetyltransferase 50, NatE catalytic subunit* | ZDB-GENE-040801-142 |
| 360 | *ncam1* | *neural cell adhesion molecule 1a* | ZDB-GENE-990415-31 |
| 361 | *nccrp1* | *nonspecific cytotoxic cell receptor protein 1* | ZDB-GENE-000210-13 |
| 362 | *ncf1* | *neutrophil cytosolic factor 1* | ZDB-GENE-031006-6 |
| 363 | *ncor2* | *nuclear receptor co-repressor 2* | ZDB-GENE-030616-81 |
| 364 | *nfe2* | *nuclear factor, erythroid-derived 2* | ZDB-GENE-030124-1 |
| 365 | *nitr9* | *novel immune-type receptor 9* | ZDB-GENE-041001-6 |
| 366 | *nop58* | *NOP58 ribonucleoprotein homolog (yeast)* | ZDB-GENE-040426-2140 |
| 367 | *notch3* | *notch homolog 3* | ZDB-GENE-000329-5 |
| 368 | *npl* | *N-acetylneuraminate pyruvate lyase (dihydrodipicolinate synthase)* | ZDB-GENE-030131-926 |
| 369 | *npsnl* | *nephrosin-like* | ZDB-GENE-050318-7 |
| 370 | *nr2f1b* | *nuclear receptor subfamily 2, group F, member 1b* | ZDB-GENE-040426-1438 |
| 371 | *nr4a2b* | *nuclear receptor subfamily 4, group A, member 2b* | ZDB-GENE-040718-103 |
| 372 | *nrp1a* | *neuropilin 1a* | ZDB-GENE-030519-2 |
| 373 | *nrp2a* | *neuropilin 2a* | ZDB-GENE-040611-2 |
| 374 | *nt5c2l1* | *5'-nucleotidase, cytosolic II, like 1* | ZDB-GENE-031006-8 |
| 375 | *nucb2a* | *nucleobindin 2a* | ZDB-GENE-030826-14 |
| 376 | *nutf2l* | *nuclear transport factor 2, like* | ZDB-GENE-020416-1 |
| 377 | *orc6l* | *origin recognition complex, subunit 6* | ZDB-GENE-030131-2976 |
| 378 | *osr1* | *odd-skipped related 1 (Drosophila)* | ZDB-GENE-070321-1 |
| 379 | *pak2a* | *p21 (CDKN1A)-activated kinase 2a* | ZDB-GENE-021011-2 |
| 380 | *pak2b* | *p21 (CDKN1A)-activated kinase 2b* | ZDB-GENE-030131-3759 |
| 381 | *pald* | *paladin* | ZDB-GENE-030804-26 |
| 382 | *papss2* | *3'-phosphoadenosine 5'-phosphosulfate synthase 2b* | ZDB-GENE-010323-5 |
| 383 | *pcna* | *proliferating cell nuclear antigen* | ZDB-GENE-000210-8 |
| 384 | *pde7a* | *phosphodiesterase 7A* | ZDB-GENE-031222-10 |
| 385 | *pdlim1* | *PDZ and LIM domain 1 (elfin)* | ZDB-GENE-030131-5227 |
| 386 | *pdlim2* | *PDZ and LIM domain 2 (mystique)* | ZDB-GENE-070308-4 |
| 387 | *pdlim3a* | *PDZ and LIM domain 3a* | ZDB-GENE-050505-1 |
| 388 | *pdlim3b* | *PDZ and LIM domain 3b* | ZDB-GENE-060130-104 |
| 389 | *pdlim4* | *PDZ and LIM domain 4* | ZDB-GENE-070308-5 |
| 390 | *pdlim5* | *PDZ and LIM domain 5a* | ZDB-GENE-040718-401 |
| 391 | *pepd* | *peptidase D* | ZDB-GENE-030131-9444 |
| 392 | *pfkfb4l* | *6-phosphofructo-2-kinase/fructose-2,6-biphosphatase 4, like* | ZDB-GENE-031031-4 |
| 393 | *pfn1* | *profilin 1* | ZDB-GENE-031002-33 |
| 394 | *pfn2* | *profilin 2* | ZDB-GENE-040115-4 |
| 395 | *pgd* | *phosphogluconate hydrogenase* | ZDB-GENE-040426-2807 |
| 396 | *pglyrp2* | *peptidoglycan recognition protein 2* | ZDB-GENE-071227-1 |
| 397 | *pglyrp5* | *peptidoglycan recognition protein 5* | ZDB-GENE-050419-71 |
| 398 | *pglyrp6* | *peptidoglycan recognition protein 6* | ZDB-GENE-071227-2 |
| 399 | *pgm3* | *phosphoglucomutase 3* | ZDB-GENE-041024-13 |
| 400 | *phyhipl* | *phytanoyl-CoA 2-hydroxylase interacting protein-like b* | ZDB-GENE-040927-27 |
| 401 | *pigq* | *phosphatidylinositol glycan, class Q* | ZDB-GENE-030131-9793 |
| 402 | *pitpna* | *phosphatidylinositol transfer protein, alpha a* | ZDB-GENE-040426-744 |
| 403 | *plag1* | *pleiomorphic adenoma gene 1* | ZDB-GENE-060302-2 |
| 404 | *plagl2* | *pleiomorphic adenoma gene-like 2* | ZDB-GENE-020806-1 |
| 405 | *plagx* | *pleiomorphic adenoma gene X* | ZDB-GENE-030131-839 |
| 406 | *plcg1* | *phospholipase C, gamma 1* | ZDB-GENE-030421-3 |
| 407 | *plek* | *pleckstrin* | ZDB-GENE-040426-1506 |
| 408 | *plekhg5* | *pleckstrin homology domain containing, family G (with RhoGef domain) member 5a* | ZDB-GENE-060503-766 |
| 409 | *plekhh1* | *pleckstrin homology domain containing, family H (with MyTH4 domain) member 1* | ZDB-GENE-061219-1 |
| 410 | *pls1* | *plastin 1 (I isoform)* | ZDB-GENE-030131-6205 |
| 411 | *plxnd1* | *plexin D1* | ZDB-GENE-040426-1828 |
| 412 | *pnrc2* | *proline-rich nuclear receptor coactivator 2* | ZDB-GENE-030131-5475 |
| 413 | *ppox* | *protoporphyrinogen oxidase* | ZDB-GENE-051120-90 |
| 414 | *ppp1r14b* | *protein phosphatase 1, regulatory (inhibitor) subunit 14Bb* | ZDB-GENE-030616-595 |
| 415 | *prcp* | *prolylcarboxypeptidase (angiotensinase C)* | ZDB-GENE-040718-447 |
| 416 | *prdm15* | *PR domain containing 15* | ZDB-GENE-080618-4 |
| 417 | *prdx2* | *peroxiredoxin 2* | ZDB-GENE-030326-2 |
| 418 | *prdx3* | *peroxiredoxin 3* | ZDB-GENE-030826-18 |
| 419 | *prdx5* | *peroxiredoxin 5* | ZDB-GENE-050522-159 |
| 420 | *prim1* | *primase polypeptide 1* | ZDB-GENE-990603-6 |
| 421 | *prkcd* | *protein kinase C, delta a* | ZDB-GENE-030131-6503 |
| 422 | *prkci* | *protein kinase C, iota* | ZDB-GENE-011105-1 |
| 423 | *prox1* | *prospero-related homeobox gene 1a* | ZDB-GENE-980526-397 |
| 424 | *psap* | *prosaposin* | ZDB-GENE-020108-1 |
| 425 | *ptena* | *phosphatase and tensin homolog A* | ZDB-GENE-030131-3776 |
| 426 | *ptgs1* | *prostaglandin-endoperoxide synthase 1* | ZDB-GENE-020530-1 |
| 427 | *ptplad1* | *protein tyrosine phosphatase-like A domain containing 1* | ZDB-GENE-040426-1200 |
| 428 | *ptpn2l* | *protein tyrosine phosphatase, non-receptor type 2, b* | ZDB-GENE-030909-8 |
| 429 | *ptpn6* | *protein tyrosine phosphatase, non-receptor type 6* | ZDB-GENE-030131-7513 |
| 430 | *ptprc* | *protein tyrosine phosphatase, receptor type, C* | ZDB-GENE-050208-585 |
| 431 | *ptprja* | *protein tyrosine phosphatase, receptor type, J a* | ZDB-GENE-030131-8301 |
| 432 | *ptprjb* | *protein tyrosine phosphatase, receptor type, J b* | ZDB-GENE-090304-1 |
| 433 | *ptprk* | *protein tyrosine phosphatase, receptor type, K* | ZDB-GENE-030131-9834 |
| 434 | *ptprm* | *protein tyrosine phosphatase, receptor type, M, a* | ZDB-GENE-031113-24 |
| 435 | *ptrf* | *polymerase I and transcript release factor b* | ZDB-GENE-030131-5509 |
| 436 | *rab13* | *RAB13, member RAS oncogene family* | ZDB-GENE-030826-30 |
| 437 | *rab14* | *RAB14, member RAS oncogene family* | ZDB-GENE-030826-20 |
| 438 | *rab5c* | *RAB5C, member RAS oncogene family* | ZDB-GENE-031118-30 |
| 439 | *rag1* | *recombination activating gene 1* | ZDB-GENE-990415-234 |
| 440 | *rag2* | *recombination activating gene 2* | ZDB-GENE-990415-235 |
| 441 | *rap1b* | *RAS related protein 1b* | ZDB-GENE-030131-9662 |
| 442 | *rasgrp3* | *RAS guanyl releasing protein 3 (calcium and DAG-regulated)* | ZDB-GENE-070424-82 |
| 443 | *rassf1* | *Ras association (RalGDS/AF-6) domain family 1* | ZDB-GENE-040912-14 |
| 444 | *rbp4* | *retinol binding protein 4, plasma* | ZDB-GENE-000210-19 |
| 445 | *rcn3* | *reticulocalbin 3, EF-hand calcium binding domain* | ZDB-GENE-040625-175 |
| 446 | *rdh1l* | *dehydrogenase/reductase (SDR family) member 9* | ZDB-GENE-030131-1249 |
| 447 | *rdx* | *moesin a* | ZDB-GENE-021211-2 |
| 448 | *rh30* | *Rh blood group, D antigen* | ZDB-GENE-051213-1 |
| 449 | *rhag* | *Rhesus blood group-associated glycoprotein* | ZDB-GENE-030131-8229 |
| 450 | *rhof* | *ras homolog gene family, member F* | ZDB-GENE-050522-280 |
| 451 | *rhogb* | *ras homolog gene family, member Gb* | ZDB-GENE-030131-8877 |
| 452 | *rhoua* | *ras homolog gene family, member Ua* | ZDB-GENE-040618-3 |
| 453 | *rnaseh2b* | *ribonuclease H2, subunit B* | ZDB-GENE-040426-1090 |
| 454 | *rnasel1* | *ribonuclease like 1* | ZDB-GENE-030131-1049 |
| 455 | *robo4* | *roundabout homolog 4* | ZDB-GENE-020809-1 |
| 456 | *runx1* | *runt-related transcription factor 1* | ZDB-GENE-000605-1 |
| 457 | *samsn1a* | *SAM domain, SH3 domain and nuclear localisation signals, 1a* | ZDB-GENE-030131-8639 |
| 458 | *sb:cb101* | sb:cb101 | ZDB-GENE-030131-9648 |
| 459 | *sb:cb1043* | sb:cb1043 | ZDB-GENE-040108-6 |
| 460 | *sb:cb1067* | sb:cb1067 | ZDB-GENE-040108-10 |
| 461 | *sb:cb124* | *si:dkey-27i16.2* | ZDB-GENE-030131-9667 |
| 462 | *sb:cb166* | sb:cb166 | ZDB-GENE-030131-9690 |
| 463 | *sb:cb176* | sb:cb176 | ZDB-GENE-030131-9699 |
| 464 | *sb:cb234* | sb:cb234 | ZDB-GENE-030131-9731 |
| 465 | *sb:cb247* | sb:cb247 | ZDB-GENE-030131-9739 |
| 466 | *sb:cb31* | sb:cb31 | ZDB-GENE-030131-9771 |
| 467 | *sb:cb375* | *si:ch73-9j13.1* | ZDB-GENE-030131-9805 |
| 468 | *sb:cb429* | sb:cb429 | ZDB-GENE-030131-9836 |
| 469 | *sb:cb458* | *si:ch211-204c21.1* | ZDB-GENE-030429-35 |
| 470 | *sb:cb662* | *2',3'-cyclic nucleotide 3' phosphodiesterase* | ZDB-GENE-030521-29 |
| 471 | *sb:cb90* | *zgc:101748* | ZDB-GENE-040912-53 |
| 472 | *sb:cb932* | *actin filament associated protein 1-like 1b* | ZDB-GENE-061013-428 |
| 473 | *sb:cb975* | *si:ch211-197h24.9* | ZDB-GENE-031116-15 |
| 474 | *sb:eu1033* | *sb:eu1033* | ZDB-GENE-060130-30 |
| 475 | *sb:eu434* | sb:eu434 | ZDB-GENE-060130-57 |
| 476 | *sb:eu551* | sb:eu551 | ZDB-GENE-060130-99 |
| 477 | *sb:eu718* | *endothelium-specific receptor tyrosine kinase 1* | ZDB-GENE-990415-55 |
| 478 | *sb:eu768* | sb:eu768 | ZDB-GENE-060130-172 |
| 479 | *sc:d0823* | *roundabout homolog 4* | ZDB-GENE-020809-1 |
| 480 | *sc:d808* | sc:d808 | ZDB-GENE-080303-19 |
| 481 | *scpep1* | *serine carboxypeptidase 1* | ZDB-GENE-040426-890 |
| 482 | *sdc2* | *syndecan 2* | ZDB-GENE-021206-3 |
| 483 | *selenbp1* | *selenium binding protein 1* | ZDB-GENE-040426-1436 |
| 484 | *selj* | *selenoprotein J* | ZDB-GENE-030131-4163 |
| 485 | *selt1a* | *selenoprotein T, 1a* | ZDB-GENE-030327-6 |
| 486 | *selt1b* | *selenoprotein T, 1b* | ZDB-GENE-030411-1 |
| 487 | *seph* | *selenoprotein H* | ZDB-GENE-030411-2 |
| 488 | *sepp1a* | *selenoprotein P, plasma, 1a* | ZDB-GENE-030311-1 |
| 489 | *nr4a2b* | *nuclear receptor subfamily 4, group A, member 2b* | ZDB-GENE-040718-103 |
| 490 | *slc4a1* | *solute carrier family 4, anion exchanger, member 1a* | ZDB-GENE-010525-1 |
| 491 | *sept9a* | *septin 9a* | ZDB-GENE-030131-9187 |
| 492 | *setd2* | *SET domain containing 2* | ZDB-GENE-030131-2140 |
| 493 | *sfrp5* | *secreted frizzled-related protein 5* | ZDB-GENE-011108-2 |
| 494 | *sgk1* | *serum/glucocorticoid regulated kinase 1* | ZDB-GENE-030131-2860 |
| 495 | *sh3bp5* | *SH3-domain binding protein 5b (BTK-associated)* | ZDB-GENE-040426-2813 |
| 496 | *sh3gl1b* | *SH3-domain GRB2-like 1b* | ZDB-GENE-031001-6 |
| 497 | *sh3gl3* | *SH3-domain GRB2-like 3* | ZDB-GENE-040121-4 |
| 498 | *shbg* | *sex hormone binding globulin* | ZDB-GENE-030131-1324 |
| 499 | *si:ch211-14k19.8* | si:ch211-14k19.8 | ZDB-GENE-051214-4 |
| 500 | *si:ch211-212m21.5* | si:ch211-212m21.5 | ZDB-GENE-030131-9807 |
| 501 | *si:ch211-215f19.1* | *adrenergic receptor, beta 3a* | ZDB-GENE-080917-21 |
| 502 | *si:ch211-217g15.2* | si:ch211-217g15.2 | ZDB-GENE-030616-618 |
| 503 | *si:ch211-268b13.2* | si:ch211-268b13.2 | ZDB-GENE-030219-15 |
| 504 | *si:dkey-146n1.1* | *podocalyxin-like* | ZDB-GENE-030131-2805 |
| 505 | *si:dkey-204f11.66* | si:dkey-204f11.66 | ZDB-GENE-040724-129 |
| 506 | *si:dkey-220f10.6* | si:dkey-220f10.6 | ZDB-GENE-041111-253 |
| 507 | *si:dkey-222f8.3* | si:dkey-222f8.3 | ZDB-GENE-030131-3658 |
| 508 | *si:dkey-261h17.1* | si:dkey-261h17.1 | ZDB-GENE-030829-14 |
| 509 | *si:dkey-33c9.4* | si:dkey-33c9.4 | ZDB-GENE-070727-1 |
| 510 | *si:dkey-76k16.7* | si:dkey-76k16.7 | ZDB-GENE-030131-9795 |
| 511 | *si:dkey-78p20.2* | si:dkey-78p20.2 | ZDB-GENE-050309-166 |
| 512 | *si:dkey-8l13.4* | si:dkey-8l13.4 | ZDB-GENE-030131-3587 |
| 513 | *si:dkey-91i17.1* | *NOP2 nucleolar protein homolog (yeast)* | ZDB-GENE-050309-7 |
| 514 | *sid4* | *secreted immunoglobulin domain 4* | ZDB-GENE-050726-3 |
| 515 | *skp2* | *S-phase kinase-associated protein 2 (p45)* | ZDB-GENE-030219-158 |
| 516 | *sla1* | *Src-like-adaptor 1* | ZDB-GENE-050904-3 |
| 517 | *slc10a4* | *solute carrier family 10 (sodium/bile acid cotransporter family), member 4* | ZDB-GENE-041014-249 |
| 518 | *slc11a2* | *solute carrier family 11 (proton-coupled divalent metal ion transporters), member 2* | ZDB-GENE-021115-1 |
| 519 | *slc12a10.1* | *solute carrier family 12 (sodium/potassium/chloride transporters), member 10.1* | ZDB-GENE-060503-330 |
| 520 | *slc12a3* | *solute carrier family 12 (sodium/chloride transporters), member 3* | ZDB-GENE-030131-9505 |
| 521 | *slc20a1a* | *solute carrier family 20, member 1a* | ZDB-GENE-040426-2217 |
| 522 | *slc25a37* | *solute carrier family 25, member 37* | ZDB-GENE-031118-202 |
| 523 | *slc2a1* | *solute carrier family 2 (facilitated glucose transporter), member 1a* | ZDB-GENE-030131-3158 |
| 524 | *slc43a1a* | *solute carrier family 43, member 1a* | ZDB-GENE-030131-6327 |
| 525 | *slc4a1* | *solute carrier family 4, anion exchanger, member 1a* | ZDB-GENE-010525-1 |
| 526 | *slc4a2a* | *solute carrier family 4, anion exchanger, member 2a* | ZDB-GENE-051101-2 |
| 527 | *slc4a2b* | *solute carrier family 4, anion exchanger, member 2b* | ZDB-GENE-030429-14 |
| 528 | *slc6a6* | *solute carrier family 6 (neurotransmitter transporter, taurine), member 6b* | ZDB-GENE-030131-3077 |
| 529 | *slc8a1b* | *solute carrier family 8 (sodium/calcium exchanger), member 4b* | ZDB-GENE-050809-103 |
| 530 | *slc8a4a* | *solute carrier family 8 (sodium/calcium exchanger), member 4a* | ZDB-GENE-060110-2 |
| 531 | *slc9a1* | *solute carrier family 9 (sodium/hydrogen exchanger), member 1* | ZDB-GENE-080225-42 |
| 532 | *slc9a8* | *solute carrier family 9 (sodium/hydrogen exchanger), member 8* | ZDB-GENE-041212-7 |
| 533 | *slmo2* | *slowmo homolog 2 (Drosophila)* | ZDB-GENE-031002-13 |
| 534 | *smad5* | *MAD homolog 5 (Drosophila)* | ZDB-GENE-990603-9 |
| 535 | *smc2* | *structural maintenance of chromosomes 2* | ZDB-GENE-030131-105 |
| 536 | *smchd1* | *structural maintenance of chromosomes flexible hinge domain containing 1* | ZDB-GENE-050211-6 |
| 537 | *smox* | *spermine oxidase* | ZDB-GENE-031201-3 |
| 538 | *snrk1* | *SNF related kinase 1* | ZDB-GENE-040426-1724 |
| 539 | *snx1* | *sorting nexin 1* | ZDB-GENE-060302-3 |
| 540 | *snx5* | *sorting nexin 5* | ZDB-GENE-040426-2857 |
| 541 | *snx8* | *sorting nexin 8* | ZDB-GENE-031202-1 |
| 542 | *sox18* | *SRY-box containing gene 18* | ZDB-GENE-080725-1 |
| 543 | *sox7* | *SRY-box containing gene 7* | ZDB-GENE-040109-4 |
| 544 | *spi1* | *spleen focus forming virus (SFFV) proviral integration oncogene spi1* | ZDB-GENE-980526-164 |
| 545 | *spns2* | *spinster homolog 2 (Drosophila)* | ZDB-GENE-030131-5843 |
| 546 | *sptb* | *spectrin, beta, erythrocytic* | ZDB-GENE-000906-1 |
| 547 | *ssr3* | *signal sequence receptor, gamma* | ZDB-GENE-030131-9134 |
| 548 | *stab1l* | *stabilin 1-like* | ZDB-GENE-050208-554 |
| 549 | *sult2st1* | *sulfotransferase family 2, cytosolic sulfotransferase 1* | ZDB-GENE-030219-114 |
| 550 | *sult6b1* | *sulfotransferase family, cytosolic, 6b, member 1* | ZDB-GENE-050417-228 |
| 551 | *suv39h1b* | *suppressor of variegation 3-9 homolog 1b* | ZDB-GENE-030131-5105 |
| 552 | *tagln2* | *transgelin 2* | ZDB-GENE-020802-2 |
| 553 | *tal1* | *T-cell acute lymphocytic leukemia 1* | ZDB-GENE-980526-501 |
| 554 | *tbx20* | *T-box 20* | ZDB-GENE-000427-7 |
| 555 | *tbx6* | *T-box gene 6* | ZDB-GENE-020416-5 |
| 556 | *tfe3a* | *transcription factor binding to IGHM enhancer 3a* | ZDB-GENE-010919-2 |
| 557 | *tfr1a* | *transferrin receptor 1a* | ZDB-GENE-041220-1 |
| 558 | *tgfbr2* | *transforming growth factor, beta receptor II* | ZDB-GENE-980526-375 |
| 559 | *thbs1* | *thrombospondin 1* | ZDB-GENE-020708-1 |
| 560 | *tie1* | *endothelium-specific receptor tyrosine kinase 1* | ZDB-GENE-990415-55 |
| 561 | *tie2* | *endothelium-specific receptor tyrosine kinase 2* | ZDB-GENE-990415-56 |
| 562 | *tjp1* | *tight junction protein 1 (zona occludens 1)* | ZDB-GENE-031001-2 |
| 563 | *tjp1b* | *tight junction protein 1b* | ZDB-GENE-070925-1 |
| 564 | *tjp2a* | *tight junction protein 2a (zona occludens 2)* | ZDB-GENE-070925-2 |
| 565 | *tjp3* | *tight junction protein 3* | ZDB-GENE-030828-10 |
| 566 | *tln1* | *talin 1* | ZDB-GENE-031002-48 |
| 567 | *tlr18* | *toll-like receptor 18* | ZDB-GENE-040220-2 |
| 568 | *tlr3* | *toll-like receptor 3* | ZDB-GENE-040219-7 |
| 569 | *tlr4a* | *toll-like receptor 4b, duplicate a* | ZDB-GENE-040219-8 |
| 570 | *tlr4b* | *toll-like receptor 4b, duplicate b* | ZDB-GENE-040219-9 |
| 571 | *tlr7* | *toll-like receptor 7* | ZDB-GENE-040219-11 |
| 572 | *tlr8a* | *toll-like receptor 8a* | ZDB-GENE-040219-13 |
| 573 | *tlr8b* | *toll-like receptor 8b* | ZDB-GENE-040219-12 |
| 574 | *tlr9* | *toll-like receptor 9* | ZDB-GENE-040219-10 |
| 575 | *tmem88a* | *transmembrane protein 88 a* | ZDB-GENE-040426-1808 |
| 576 | *tnfa* | *tumor necrosis factor a (TNF superfamily, member 2)* | ZDB-GENE-050317-1 |
| 577 | *tpte* | *transmembrane phosphatase with tensin homology* | ZDB-GENE-030131-5503 |
| 578 | *trac* | *T cell receptor alpha constant* | ZDB-GENE-030616-200 |
| 579 | *traf4a* | *tnf receptor-associated factor 4a* | ZDB-GENE-040308-1 |
| 580 | *trim33* | *tripartite motif-containing 33* | ZDB-GENE-030131-2773 |
| 581 | *trpc6* | *transient receptor potential cation channel, subfamily C, member 6* | ZDB-GENE-040724-114 |
| 582 | *twist1a* | *twist1a* | ZDB-GENE-000210-6 |
| 583 | *twist1b* | twist1b | ZDB-GENE-050417-357 |
| 584 | *twist2* | twist2 | ZDB-GENE-980526-235 |
| 585 | *txnip* | *thioredoxin interacting protein a* | ZDB-GENE-030804-10 |
| 586 | *txnrd1* | *thioredoxin reductase 1* | ZDB-GENE-030327-2 |
| 587 | *txnrd3* | *thioredoxin reductase 3* | ZDB-GENE-030327-3 |
| 588 | *tyms* | *thymidylate synthase* | ZDB-GENE-040426-59 |
| 589 | *tyrobp* | *TYRO protein tyrosine kinase binding protein* | ZDB-GENE-061130-2 |
| 590 | *ucp2* | *uncoupling protein 2* | ZDB-GENE-990708-8 |
| 591 | *udu* | *gon-4-like (C.elegans)* | ZDB-GENE-070117-2447 |
| 592 | *ugt1aa* | *UDP glucuronosyltransferase 1 family, polypeptide B1* | ZDB-GENE-080227-10 |
| 593 | *urod* | *uroporphyrinogen decarboxylase* | ZDB-GENE-000208-18 |
| 594 | *uros* | *uroporphyrinogen III synthase* | ZDB-GENE-040323-2 |
| 595 | *vasn* | *vasorin* | ZDB-GENE-050522-43 |
| 596 | *vegfaa* | *vascular endothelial growth factor Aa* | ZDB-GENE-990415-273 |
| 597 | *vegfab* | *vascular endothelial growth factor Ab* | ZDB-GENE-030131-4605 |
| 598 | *vegfc* | *vascular endothelial growth factor c* | ZDB-GENE-040303-4 |
| 599 | *vsg1* | *vessel-specific 1* | ZDB-GENE-060616-96 |
| 600 | *wasa* | *Wiskott-Aldrich syndrome (eczema-thrombocytopenia) a* | ZDB-GENE-081104-419 |
| 601 | *wasb* | *Wiskott-Aldrich syndrome (eczema-thrombocytopenia) b* | ZDB-GENE-030131-7098 |
| 602 | *wu:cegs2569* | *DIP2 disco-interacting protein 2 homolog C (Drosophila)* | ZDB-GENE-031118-39 |
| 603 | *wu:fb17g07* | wu:fb17g07 | ZDB-GENE-030131-226 |
| 604 | *wu:fb18c02* | wu:fb18c02 | ZDB-GENE-030131-247 |
| 605 | *wu:fb23h02* | wu:fb23h02 | ZDB-GENE-050429-1 |
| 606 | *wu:fb62f03* | *zgc:92745* | ZDB-GENE-040801-12 |
| 607 | *wu:fc25e04* | wu:fc25e04 | ZDB-GENE-030131-3039 |
| 608 | *wu:fd56d05* | wu:fd56d05 | ZDB-GENE-030131-4728 |
| 609 | *wu:fj67h05* | wu:fj67h05 | ZDB-GENE-030131-9394 |
| 610 | *wu:fj78f01* | *si:dkeyp-66d7.2* | ZDB-GENE-030131-9438 |
| 611 | *yars* | *tyrosyl-tRNA synthetase* | ZDB-GENE-030425-2 |
| 612 | *yrk* | *Yes-related kinase* | ZDB-GENE-030131-9517 |
| 613 | *zbtb2b* | *zinc finger and BTB domain containing 2b* | ZDB-GENE-040426-2753 |
| 614 | *zfp36l1* | *zinc finger protein 36, C3H type-like 1a* | ZDB-GENE-030131-9860 |
| 615 | *zfpm1* | *zinc finger protein, multitype 1* | ZDB-GENE-050419-238 |
| 616 | *zgc:101000* | zgc:101000 | ZDB-GENE-040808-35 |
| 617 | *zgc:101650* | zgc:101650 | ZDB-GENE-041212-68 |
| 618 | *zgc:103419* | *claudin 5b* | ZDB-GENE-041010-140 |
| 619 | *zgc:103456* | *translocator protein* | ZDB-GENE-041010-125 |
| 620 | *zgc:103467* | *myosin, light chain 9a, regulatory* | ZDB-GENE-041010-120 |
| 621 | *zgc:110010* | *glutathione reductase* | ZDB-GENE-050522-116 |
| 622 | *zgc:110219* | *aminolevulinate dehydratase* | ZDB-GENE-050417-123 |
| 623 | *zgc:110239* | zgc:110239 | ZDB-GENE-050417-107 |
| 624 | *zgc:110340* | zgc:110340 | ZDB-GENE-050320-36 |
| 625 | *zgc:110459* | *pleckstrin homology-like domain, family A, member 2* | ZDB-GENE-050522-73 |
| 626 | *zgc:112009* | zgc:112009 | ZDB-GENE-050417-172 |
| 627 | *zgc:112062* | *cysteinyl leukotriene receptor 1* | ZDB-GENE-050522-250 |
| 628 | *zgc:112397* | *tripartite motif containing 35-7* | ZDB-GENE-050522-333 |
| 629 | *zgc:152830* | zgc:152830 | ZDB-GENE-090312-55 |
| 630 | *zgc:153077* | *solute carrier family 17 (anion/sugar transporter), member 5* | ZDB-GENE-060929-1158 |
| 631 | *zgc:153439* | *transmembrane protein 14C* | ZDB-GENE-060825-156 |
| 632 | *zgc:171802* | *G protein-coupled receptor 84* | ZDB-GENE-070928-33 |
| 633 | *zgc:55262* | zgc:55262 | ZDB-GENE-040426-2814 |
| 634 | *zgc:55307* | *nuclear receptor coactivator 4* | ZDB-GENE-040426-689 |
| 635 | *zgc:55891* | zgc:55891 | ZDB-GENE-040426-2022 |
| 636 | *zgc:56310* | *replication protein A3* | ZDB-GENE-040426-977 |
| 637 | *zgc:56419* | zgc:56419 | ZDB-GENE-030131-8741 |
| 638 | *zgc:56530* | zgc:56530 | ZDB-GENE-040426-1065 |
| 639 | *zgc:63474* | zgc:63474 | ZDB-GENE-040426-1129 |
| 640 | *zgc:63495* | *serine/threonine kinase 10* | ZDB-GENE-040426-1136 |
| 641 | *fcer1gl* | *Fc receptor, IgE, high affinity I, gamma polypeptide like* | ZDB-GENE-070502-4 |
| 642 | *zgc:63602* | *cytochrome P450, family 46, subfamily A, polypeptide 2* | ZDB-GENE-040426-1184 |
| 643 | *zgc:63629* | zgc:63629 | ZDB-GENE-040426-2689 |
| 644 | *zgc:63633* | *RAS-like, family 12* | ZDB-GENE-040426-1201 |
| 645 | *zgc:63700* | *aquaporin 7* | ZDB-GENE-030131-6461 |
| 646 | *zgc:63734* | *tropomyosin 4b* | ZDB-GENE-030131-2964 |
| 647 | *zgc:64022* | zgc:64022 | ZDB-GENE-040426-1340 |
| 648 | *zgc:64051* | zgc:64051 | ZDB-GENE-040426-1349 |
| 649 | *zgc:64166* | *transmembrane protein 231* | ZDB-GENE-040426-1386 |
| 650 | *zgc:66433* | zgc:66433 | ZDB-GENE-030131-9832 |
| 651 | *zgc:66441* | zgc:66441 | ZDB-GENE-030131-5559 |
| 652 | *zgc:73134* | zgc:73134 | ZDB-GENE-030131-4311 |
| 653 | *zgc:73136* | *Yip1 interacting factor homolog A (S. cerevisiae)* | ZDB-GENE-030131-6922 |
| 654 | *zgc:77001* | zgc:77001 | ZDB-GENE-040426-2565 |
| 655 | *zgc:77051* | *phospholipid scramblase 3b* | ZDB-GENE-040426-2517 |
| 656 | *zgc:77076* | *microfibrillar-associated protein 4* | ZDB-GENE-040426-2246 |
| 657 | *zgc:77234* | zgc:77234 | ZDB-GENE-040426-1807 |
| 658 | *zgc:77517* | zgc:77517 | ZDB-GENE-040426-1508 |
| 659 | *zgc:77744* | zgc:77744 | ZDB-GENE-040426-2289 |
| 660 | *zgc:77775* | zgc:77775 | ZDB-GENE-040426-1871 |
| 661 | *zgc:85890* | *aquaporin 1a, tandem duplicate 1* | ZDB-GENE-030131-7764 |
| 662 | *zgc:86776* | zgc:86776 | ZDB-GENE-040625-130 |
| 663 | *zgc:91860* | zgc:91860 | ZDB-GENE-040704-34 |
| 664 | *zgc:92470* | zgc:92470 | ZDB-GENE-040912-183 |
| 665 | *zgc:92608* | *lysozyme g-like 1* | ZDB-GENE-040718-461 |
| 666 | *zgc:92647* | zgc:92647 | ZDB-GENE-040718-131 |
| 667 | *zgc:92762* | *chloride intracellular channel 2* | ZDB-GENE-040718-299 |
| 668 | *zgc:92772* | *RAB11B, member RAS oncogene family, b* | ZDB-GENE-040718-293 |
| 669 | *znf148* | *zinc finger protein 148* | ZDB-GENE-030131-8769 |
| 670 | *znf703* | *zinc finger protein 703* | ZDB-GENE-010717-1 |
| 671 | *znfl2a* | *zinc finger-like gene 2a* | ZDB-GENE-030828-7 |
| 672 | *znfl2b* | *zinc finger-like gene 2b* | ZDB-GENE-061204-3 |
