# Supplementary material for: Reverse Genetics Screen in Zebrafish Identifies a Role of miR-142a-3p in Vascular Development and Integrity
Source: PLoS One. 2012 Dec 21;7(12):e52588. doi: 10.1371/journal.pone.0052588 (PMC3528674; doi:10.1371/journal.pone.0052588)
Supplement: Table S4 — Zebrafish putative miRNA-gene target pairs derived from miRNA seed sequence match analysis. (DOC) [file pone.0052588.s009.doc]

**Supplementary** **Table S4**: Zebrafish putative miRNA-gene target pairs derived from miRNA seed sequence match analysis.

| S.no. | miRNA Name | genes |
| --- | --- | --- |
| 1 | miR-1 | cnn3a zgc:55262 mtmr8 mdkb ptplad1 prkci bcl2l gbx2 hspa9 calm1a arrdc2 grna cyb5r4 snrk1 lamp2 ikzf1 ap2m1a dlc atp6v0c slc4a1 zgc:77744 mmp2 zgc:63700 pgd atp6v1d arrb2b csrp1 osr1 anp32b nr2f1b vegfab limk2 atp6v0a1 nutf2l tbx6 rhogb si:dkey-78p20.2 ccnd1 lcp1 bmpr2a cx43 ildr2 rcn3 bactin1 agfg1a gapdhs sox7 glcci1 prdx3 pdlim1 rap1b fli1b txnip rab13 tagln2 pls1 zgc:86776 gnai2l plagx cav1 cbfb dnajc3 nccrp1 cnn2 sgk1 zgc:56419 dfna5 slc4a2a pcna hmbsb zgc:64022 zgc:112062 prox1 htt fstl1b appb ak3 elovl1b cxcr4a entpd1 papss2 rab14 selt1b fgfrl1b zgc:153077 pak2a sh3gl1b zgc:63734 hey1 slc25a37 pfn2 hhex akap12 atp2b1b crip2 |
| 2 | miR-126 | adam8a atp1b1a prkci gats atp2b1a tjp3 gata3 cdx4 bmpr2a psap tal1 pnrc2 si:ch211-212m21.5 ahr2 rasgrp3 |
| 3 | miR-130a | slc8a1b adam8a heg eomesa atp1b1a smc2 slmo2 calub kdrl npl gats calm1a cahz cyb5r4 sh3bp5 lamp2 dnajb11 baxa zgc:66441 blvrb dlc atp6v0c fgf13l mmp2 zgc:92470 zgc:63700 hif1ab pgd mcam tfe3a arrb2b ctsc thbs1 zgc:55307 csrp1 pdlim3a osr1 lpp limk2 itga5 bhlhe40 abat nutf2l tbx6 vasn zgc:103456 si:dkey-78p20.2 hey2 arhgef1 brd2a atp2b4 tgfbr2 rab5c bmpr2a cx43 ildr2 agfg1a prdx3 itgav psap rap1b ppp1r14b txnip rab13 copb2 pls1 lgmn fli1a plagx ampd3 cbfb anxa11a prdx2 tyms mt2 epb41 kdr tlr4b arl4l cebpa mdm2 plag1 calr rhof htt ctsz fuca2 elovl1b cxcr4a vsg1 sla1 entpd1 pnrc2 mybl1 mafbb pdlim2 mpp1 zgc:171802 si:dkey-146n1.1 zgc:77051 dll4 atp2b3a cpox zgc:66433 si:dkey-220f10.6 uros pfn2 akap12 ahr2 bcl2 cd247l rasgrp3 |
| 4 | miR-133a | cnn3a lama4 prkci bcl2l gats gbx2 zgc:91860 ctsl1a gclc snx5 mmp2 krt18 mcam zgc:55307 osr1 glulb igfbp1a abat sept9a ccnd1 arhgef1 rab5c bmpr2a bactin1 copb2 twist1b pls1 spi1 hif1ab fli1a plagx dnmt4 cxcr3.2 cbfb nccrp1 ncf1 dfna5 llgl2 pgm3 ca10a krt8 calr htt atp1a1 zbtb2b cd8a si:dkey-8l13.4 fhl papss2 myct1 pdlim2 mpp1 si:dkey-146n1.1 cdh5 cpne1 alp si:ch211-212m21.5 pfn2 akap12 slc43a1a atp2b1b nrp1a birc5a |
| 5 | miR-133b | cnn3a lama4 prkci bcl2l gats gbx2 zgc:91860 ctsl1a gclc snx5 mmp2 krt18 mcam zgc:55307 osr1 glulb igfbp1a abat sept9a ccnd1 arhgef1 rab5c bmpr2a bactin1 copb2 twist1b pls1 spi1 hif1ab fli1a plagx dnmt4 cxcr3.2 cbfb nccrp1 ncf1 dfna5 llgl2 pgm3 ca10a krt8 calr htt atp1a1 zbtb2b cd8a si:dkey-8l13.4 fhl papss2 myct1 pdlim2 mpp1 si:dkey-146n1.1 cdh5 cpne1 alp si:ch211-212m21.5 pfn2 akap12 slc43a1a atp2b1b nrp1a birc5a |
| 6 | miR-133c | cnn3a lama4 prkci bcl2l gats gbx2 zgc:91860 ctsl1a gclc snx5 mmp2 krt18 mcam zgc:55307 osr1 glulb igfbp1a abat sept9a ccnd1 arhgef1 rab5c bmpr2a bactin1 copb2 twist1b pls1 spi1 hif1ab fli1a plagx dnmt4 cxcr3.2 cbfb nccrp1 ncf1 dfna5 llgl2 pgm3 ca10a krt8 calr htt atp1a1 zbtb2b cd8a si:dkey-8l13.4 fhl papss2 myct1 pdlim2 mpp1 si:dkey-146n1.1 cdh5 cpne1 alp si:ch211-212m21.5 pfn2 akap12 slc43a1a atp2b1b nrp1a birc5a |
| 7 | miR-138 | slmo2 kdrl traf4a cyb5r4 itga2b rhag baxa ap2m1a atp6v0c slc4a1 hif1ab atp6v1d csrp1 osr1 bhlhe40 tbx6 vasn ptpn2l pitpna prdx3 lmo2 zgc:92772 fli1a twist1a ncf1 gypc kdr hmbsb plag1 calr hapln3 nitr9 crlf3 lpar1 fzd2 gtpbp1 si:dkey-146n1.1 zgc:64051 sid4 slc25a37 wu:fb17g07 dll4 csf1r akap12 slc43a1a prim1 |
| 8 | miR-142a-3p | heg atp1b1a calub kdrl ipo9 dusp1 npl bcl2l tlr3 tjp3 dnajb11 gfap krt18 csrp1 vegfab limk2 sept9a calm1a hey2 ccnd1 atp2b4 pigq rab5c cx43 itgav zgc:56530 ppp1r14b rab13 il13ra2 pls1 plagx rhoua llgl2 prcp ilk suv39h1b hmbsb hapln3 htt crlf3 ifi30 mybl1 txnrd1 mpp1 si:dkey-146n1.1 cdh5 hey1 slc25a37 hspa12b pfn2 crip2 rasgrp3 |
| 9 | miR-142a-5p | adam8a zgc:55262 tyrobp map1lc3b eomesa atp1b1a nitr9 sh3gl3 smc2 calub ldb2a ipo9 npl prkci bcl2l gats cahz sh3bp5 atp2b1a zgc:91860 dhfr lamp2 hspa5 tlr3 klfd zgc:73136 rhag dnajb11 zgc:66441 ctsl1a atp6v0c slc4a1 gfap tnfa gata3 zgc:92762 mmp2 zgc:92470 hif1ab trim33 glud1a flt4 mcam arrb2b ctsc mapk1 hmox1 pdlim3a fabp11a nr2f1b glulb zgc:77517 atp6v0a1 igfbp1a sept9a vasn arl4a rhogb rdh1l mcm3 ccnd1 zgc:92647 pitpna arhgef1 ireb2 brd2a atp2b4 tgfbr2 bmpr2a cx43 znf703 rap1b krcp ppp1r14b pls1 smox hsd3b7 zgc:86776 plagx rhoua lgals9l1 sult2st1 cbfb cnn2 pak2b ncf1 sgk1 gypc prkcd zgc:56419 arl4l sptb cebpa prcp lmo4 snx1 ilk suv39h1b hapln3 zgc:64022 glrx5 znf148 zgc:92608 seph micall2 ctsz fstl1b ak3 tal1 ca5 zbtb2b fzd2 entpd1 cd8a pnrc2 bxdc1 rab14 gng2 aplnra gtpbp1 fgfrl1b zgc:153077 mafbb pdlim2 mpp1 zgc:63734 si:dkey-146n1.1 hey1 pdlim4 slc25a37 drl si:ch211-212m21.5 hspa12b hoxc9a zgc:66433 si:dkey-220f10.6 uros grnb pfn2 slc43a1a atp2b1b ahr2 birc5a plagl2 zgc:55891 rasgrp3 |
| 10 | miR-143 | jak2a map1lc3b calub ptplad1 traf4a bcl2l arrdc2 sh3bp5 atp2b1a elovl1a slc4a1 gfap gata3 snx5 zgc:92470 tbx20 krt18 atp6v1d mapk1 limk2 bhlhe40 abat nutf2l ccnd1 zgc:110010 pitpna arhgef1 atp2b4 rab5c cx43 itgav rap1b nat13 aplnrb zgc:92772 pls1 fli1a cndp2 plagx dnmt4 cbfb pak2b zgc:56419 cebpa gata2a lgals2a plag1 cybb epas1 micall2 freqa tal1 blf zbtb2b rab14 mafbb pak2a itm2bb si:dkey-146n1.1 zgc:64051 hey1 slc25a37 si:ch211-212m21.5 pfkfb4l si:dkey-220f10.6 akap12 hbae3 zgc:63633 birc5a mych |
| 11 | miR-144 | slc8a1b adam8a ncor2 zgc:55262 lama4 csf1r heg mycb atp1b1a calub kdrl ptplad1 ldb2a ipo9 slc12a3 slc20a1a dusp1 abce1 traf4a npl prkci gats hspa9 cyb5r4 sh3bp5 zgc:91860 dhfr klfd rhag baxa ctsl1a zgc:63629 atp6v0c gclc zgc:92762 fgf13l zgc:63700 zgc:103419 krt18 atp6v1d glud1a mcam tfe3a ctsc zgc:55307 csrp1 pdlim3a zgc:56310 lta4h plek dera zgc:77775 igfbp1a sept9a si:dkey-78p20.2 mcm3 hey2 calm1a arhgef1 brd2a cx43 ildr2 mpll gapdhs itgav rap1b krcp ppp1r14b txnip mpx nat13 aplnrb smox sept2 fli1a gnai2l plagx rhoua cav1 smad5 dnmt4 pak2b sgk1 gypc adka rassf1 arl4l llgl2 pgm3 cebpa prcp lmo4 ilk suv39h1b zgc:64166 hapln3 zgc:112062 seph rhof htt crlf3 fstl1b cebpb ak3 bmp4 aspn cxcr4a vsg1 zbtb2b entpd1 si:dkey-8l13.4 papss2 bxdc1 mybl1 rab14 aplnra dhrs3b fgfrl1b mpp1 zgc:171802 sh3gl1b zgc:63734 si:dkey-146n1.1 lgals3bpb cdh5 hey1 alp setd2 glula si:ch211-212m21.5 pfkfb4l pglyrp2 lrrc15 hcst hhex akap12 crip2 ahr2 zgc:63633 mych |
| 12 | miR-145 | cnn3a jak2a heg nt5c2l1 slmo2 kdrl ptplad1 itgb3b ipo9 prkci cahz snrk1 atp2b1a klfd zgc:66441 nop58 fgf13l snx5 tbx20 zgc:63700 trim33 mcam zgc:55307 mapk1 osr1 orc6l vegfab glulb zgc:85890 limk2 slc10a4 itga5 zgc:73134 abat sept9a vasn rhogb pitpna pigq rab5c bactin1 glcci1 psap zgc:56530 clec14a lmo2 ppp1r14b pls1 fli1a twist1a plagx rhoua cav1 cyp1a cnn2 gypc rassf1 kdr arl4l pgm3 cebpa suv39h1b tmem88a calr zgc:64022 znf148 seph freqa hmbsb ak3 tal1 aspn ca5 sla1 selt1a entpd1 pnrc2 pglyrp5 mybl1 myct1 selt1b gcm2 zgc:153077 mpp1 zgc:171802 si:dkey-146n1.1 hey1 cpne1 zgc:77051 dll4 cpox setd2 glula hspa12b pfkfb4l clgn akap12 nrp1a mych |
| 13 | miR-150 | adam8a cnn3a eomesb gbx2 hspa9 arrdc2 cyb5r4 sh3bp5 atp2b1a baxa elovl1a zgc:63700 vegfab ssr3 si:dkey-78p20.2 calm1a yars clec14a ppp1r14b nat13 twist1a kpnb3 cav1 smad5 nccrp1 sfrp5 pgm3 mdm2 plag1 hapln3 her4.1 cybb micall2 cmyb freqa vsg1 pnrc2 mybl1 zgc:153077 si:dkey-146n1.1 wu:fb17g07 setd2 akap12 |
| 14 | miR-155 | spi1 ncor2 jak2a nitr9 sh3gl3 gata1 arrdc2 klfd baxa zgc:63629 dlc slc4a1 shbg gfap zgc:77744 gata3 snx5 zgc:92470 zgc:63700 hif1ab pgd arrb2b zgc:55307 glulb limk2 sept9a rhogb brd2a rab5c sox7 yars ppp1r14b txnip rab13 mpx il13ra2 aplnrb twist1b fos smox fli1a rhoua dnmt4 lgals9l1 cxcr3.2 sult2st1 dfna5 arl4l llgl2 cebpa ilk suv39h1b rag2 hmbsb c10orf119 znf148 cybb prox1 seph htt crlf3 bmp4 aspn fzd2 pnrc2 papss2 selt1b fgfrl1b mpp1 si:dkey-146n1.1 cpne1 slc25a37 id:ibd5037 pfkfb4l uros pfn2 illr1 ctsc birc5a |
| 15 | miR-15a | ncor2 cnn3a znfl2a jak2a map1lc3b slmo2 npl gats gbx2 gata1 snrk1 lamp2 hspa5 zgc:73136 dlc sept6 zgc:77744 zgc:63700 hif1ab trim33 glud1a ctsc flt1 csrp1 dab2 orc6l vegfab fth1 limk2 bhlhe40 slc20a1a vasn pitpna atp2b4 rab5c cx43 itgav pdlim1 fli1b txnip rab13 nat13 adora2aa spi1 cndp2 cbfb sgk1 kdr pgm3 hmbsb plag1 calr cybb seph rhof atp1a1 fuca2 fstl1b tal1 cxcr4a fzd2 pnrc2 mybl1 selt1b gtpbp1 fgfrl1b illr4 mpp1 zgc:171802 itm2bb sh3gl1b si:dkey-146n1.1 amotl2 lyve1l hey1 cpox setd2 si:ch211-212m21.5 hspa12b clgn si:dkey-220f10.6 pfn2 akap12 slc43a1a crip2 ahr2 vegfc fbxo5 |
| 16 | miR-16a | ncor2 cnn3a znfl2a jak2a map1lc3b slmo2 npl gats gbx2 gata1 snrk1 lamp2 hspa5 zgc:73136 dlc sept6 zgc:77744 zgc:63700 hif1ab trim33 glud1a ctsc flt1 csrp1 dab2 orc6l vegfab fth1 limk2 bhlhe40 slc20a1a vasn pitpna atp2b4 rab5c cx43 itgav pdlim1 fli1b txnip rab13 nat13 adora2aa spi1 cndp2 cbfb sgk1 kdr pgm3 hmbsb plag1 calr cybb seph rhof atp1a1 fuca2 fstl1b tal1 cxcr4a fzd2 pnrc2 mybl1 selt1b gtpbp1 fgfrl1b illr4 mpp1 zgc:171802 itm2bb sh3gl1b si:dkey-146n1.1 amotl2 lyve1l hey1 cpox setd2 si:ch211-212m21.5 hspa12b clgn si:dkey-220f10.6 pfn2 akap12 slc43a1a crip2 ahr2 vegfc fbxo5 |
| 17 | miR-16b | ncor2 cnn3a znfl2a jak2a map1lc3b slmo2 npl gats gbx2 gata1 snrk1 lamp2 hspa5 zgc:73136 dlc sept6 zgc:77744 zgc:63700 hif1ab trim33 glud1a ctsc flt1 csrp1 dab2 orc6l vegfab fth1 limk2 bhlhe40 slc20a1a vasn pitpna atp2b4 rab5c cx43 itgav pdlim1 fli1b txnip rab13 nat13 adora2aa spi1 cndp2 cbfb sgk1 kdr pgm3 hmbsb plag1 calr cybb seph rhof atp1a1 fuca2 fstl1b tal1 cxcr4a fzd2 pnrc2 mybl1 selt1b gtpbp1 fgfrl1b illr4 mpp1 zgc:171802 itm2bb sh3gl1b si:dkey-146n1.1 amotl2 lyve1l hey1 cpox setd2 si:ch211-212m21.5 hspa12b clgn si:dkey-220f10.6 pfn2 akap12 slc43a1a crip2 ahr2 vegfc fbxo5 |
| 18 | miR-16c | ncor2 cnn3a znfl2a jak2a map1lc3b slmo2 npl gats gbx2 gata1 snrk1 lamp2 hspa5 zgc:73136 dlc sept6 zgc:77744 zgc:63700 hif1ab trim33 glud1a ctsc flt1 csrp1 dab2 orc6l vegfab fth1 limk2 bhlhe40 slc20a1a vasn pitpna atp2b4 rab5c cx43 itgav pdlim1 fli1b txnip rab13 nat13 adora2aa spi1 cndp2 cbfb sgk1 kdr pgm3 hmbsb plag1 calr cybb seph rhof atp1a1 fuca2 fstl1b tal1 cxcr4a fzd2 pnrc2 mybl1 selt1b gtpbp1 fgfrl1b illr4 mpp1 zgc:171802 itm2bb sh3gl1b si:dkey-146n1.1 amotl2 lyve1l hey1 cpox setd2 si:ch211-212m21.5 hspa12b clgn si:dkey-220f10.6 pfn2 akap12 slc43a1a crip2 ahr2 vegfc fbxo5 |
| 19 | miR-181a | adam8a zgc:55262 mdkb heg map1lc3b atp1b1a zgc:110239 sh3gl3 slmo2 calub ptplad1 dusp1 traf4a npl prkci bcl2l gats arrdc2 cahz sh3bp5 atp2b1a zgc:91860 ftr82 klfd rhag dnajb11 nop58 zgc:63629 dlc slc4a1 gfap sept6 zgc:77744 fgf13l zgc:92470 hif1ab glud1a mcam ctsc hk2 zgc:55307 csrp1 nr2f1b vegfab limk2 itga5 igfbp1a bhlhe40 sept9a tbx6 rh30 arl4a si:dkey-78p20.2 cdh17 calm1a arhgef1 brd2a tgfbr2 lcp1 agfg1a sox7 rap1b fzd7b ppp1r14b txnip nat13 twist1b fos spi1 hsd3b7 fli1a twist1a zgc:86776 fbxo5 plagx rhoua cyp1a lgals9l1 lgals2b gypc prkcd adka kdr tlr4b arl4l alas2 llgl2 sptb pgm3 cebpa prcp lmo4 snx1 ilk suv39h1b tpte zgc:64022 znf148 cybb si:dkey-222f8.3 rhof htt micall2 ctsz hbbe1.1 crlf3 mmp9 appb ak3 cxcr4a cd8a si:dkey-8l13.4 fhl pnrc2 pglyrp5 papss2 bxdc1 mybl1 rab14 myct1 gcm2 mpp1 lrrc33 itm2bb elna si:dkey-146n1.1 zgc:64051 hey1 cpne1 pdlim4 cox17 slc25a37 wu:fb17g07 dll4 atp2b3a alp si:ch211-212m21.5 pfkfb4l si:dkey-220f10.6 grnb pglyrp2 pfn2 akap12 slc43a1a crip2 ahr2 zgc:55891 mych |
| 20 | miR-206 | cnn3a zgc:55262 mtmr8 mdkb ptplad1 prkci bcl2l gbx2 hspa9 calm1a arrdc2 grna cyb5r4 snrk1 lamp2 ikzf1 ap2m1a dlc atp6v0c slc4a1 zgc:77744 mmp2 zgc:63700 pgd atp6v1d arrb2b csrp1 osr1 anp32b nr2f1b vegfab limk2 atp6v0a1 nutf2l tbx6 rhogb si:dkey-78p20.2 ccnd1 lcp1 bmpr2a cx43 ildr2 rcn3 bactin1 agfg1a gapdhs sox7 glcci1 prdx3 pdlim1 rap1b fli1b txnip rab13 tagln2 pls1 zgc:86776 gnai2l plagx cav1 cbfb dnajc3 nccrp1 cnn2 sgk1 zgc:56419 dfna5 slc4a2a pcna hmbsb zgc:64022 zgc:112062 prox1 htt fstl1b appb ak3 elovl1b cxcr4a entpd1 papss2 rab14 selt1b fgfrl1b zgc:153077 pak2a sh3gl1b zgc:63734 hey1 slc25a37 pfn2 hhex akap12 atp2b1b crip2 |
| 21 | miR-20a | slc8a1b adam8a csf1r map1lc3b eomesa elovl5 sh3gl3 smc2 ldb2a slc12a3 npl prkci gats gata1 calm1a arrdc2 grna cyb5r4 dhfr lamp2 klfd rhag dnajb11 baxa ap2m1a atp6v0c gfap zgc:77744 cwf19l1 snx5 mmp2 tbx20 krt18 hif1ab atp6v1d flt4 arrb2b ctsc hk2 csrp1 gipc1 pdlim3a osr1 dab2 orc6l lta4h nr2f1b vegfab zgc:77517 limk2 itga5 atp6v0a1 abat tbx6 arl4a si:dkey-78p20.2 ccnd1 pitpna arhgef1 atp2b4 tgfbr2 cdx4 bmpr2a cx43 bactin1 znf703 glcci1 prdx3 scpep1 rap1b krcp ppp1r14b txnip rab13 nat13 twist1b zgc:92772 pls1 lgmn aldh16a1 plagx rhoua kpnb3 cav1 smad5 cbfb cnn2 pak2b sgk1 adka kdr slc4a2a arl4l alas2 pgm3 cebpa gata2a igfbp2a ilk suv39h1b plag1 calr znf148 cybb si:dkey-222f8.3 seph htt micall2 ctsz fstl1b appb tal1 zbtb2b entpd1 pnrc2 pglyrp5 samsn1a papss2 bxdc1 mybl1 rab14 selt1b gtpbp1 fgfrl1b zgc:153077 pdlim2 mpp1 sh3gl1b si:dkey-146n1.1 sid4 hey1 cpne1 pdlim4 slc25a37 si:ch211-212m21.5 hspa12b clgn si:dkey-220f10.6 uros slc43a1a crip2 ahr2 bcl2 prim1 gygl birc5a plagl2 |
| 22 | miR-20b | slc8a1b adam8a csf1r map1lc3b eomesa elovl5 sh3gl3 smc2 ldb2a slc12a3 npl prkci gats gata1 calm1a arrdc2 grna cyb5r4 dhfr lamp2 klfd rhag dnajb11 baxa ap2m1a atp6v0c gfap zgc:77744 cwf19l1 snx5 mmp2 tbx20 krt18 hif1ab atp6v1d flt4 arrb2b ctsc hk2 csrp1 gipc1 pdlim3a osr1 dab2 orc6l lta4h nr2f1b vegfab zgc:77517 limk2 itga5 atp6v0a1 abat tbx6 arl4a si:dkey-78p20.2 ccnd1 pitpna arhgef1 atp2b4 tgfbr2 cdx4 bmpr2a cx43 bactin1 znf703 glcci1 prdx3 scpep1 rap1b krcp ppp1r14b txnip rab13 nat13 twist1b zgc:92772 pls1 lgmn aldh16a1 plagx rhoua kpnb3 cav1 smad5 cbfb cnn2 pak2b sgk1 adka kdr slc4a2a arl4l alas2 pgm3 cebpa gata2a igfbp2a ilk suv39h1b plag1 calr znf148 cybb si:dkey-222f8.3 seph htt micall2 ctsz fstl1b appb tal1 zbtb2b entpd1 pnrc2 pglyrp5 samsn1a papss2 bxdc1 mybl1 rab14 selt1b gtpbp1 fgfrl1b zgc:153077 pdlim2 mpp1 sh3gl1b si:dkey-146n1.1 sid4 hey1 cpne1 pdlim4 slc25a37 si:ch211-212m21.5 hspa12b clgn si:dkey-220f10.6 uros slc43a1a crip2 ahr2 bcl2 prim1 gygl birc5a plagl2 |
| 23 | miR-210 | slc8a1b jak2a elovl5 slmo2 prdx5 calm1a gata3 zgc:92470 hif1ab arrb2b thbs1 zgc:55307 csrp1 dera glulb limk2 zgc:77775 agfg1a glcci1 psap pdlim1 rap1b tagln2 spi1 fbxo5 adka arl4l krt8 plag1 seph appb zbtb2b papss2 bxdc1 mybl1 rab14 aplnra pdlim2 mpp1 cdh5 hey1 cpne1 slc25a37 setd2 pfkfb4l clgn akap12 crip2 ahr2 bcl2 plagl2 cd247l |
| 24 | miR-221 | mdkb heg atp1b1a dusp1 traf4a npl prkci urod sh3bp5 lamp2 rhag atp6v0c gfap gclc zgc:77744 fgf13l zgc:63700 hif1ab flt4 ctsc zgc:55307 anp32b zgc:77517 limk2 zgc:77775 atp6v0a1 bhlhe40 vasn pigq znf703 sox7 psap rap1b fli1b ppp1r14b si:ch211-217g15.2 mpx jak1 fos dnmt4 sult2st1 pak2b sgk1 dfna5 slc4a2a arl4l cebpa hapln3 her4.1 zgc:64022 epas1 prox1 mmp9 hmbsb fzd2 entpd1 pnrc2 pglyrp5 mybl1 gng2 gtpbp1 fgfrl1b itm2bb si:dkey-146n1.1 zgc:64051 hey1 cpne1 setd2 si:ch211-212m21.5 hspa12b pfkfb4l si:dkey-220f10.6 uros ahr2 |
| 25 | miR-222 | mdkb heg atp1b1a dusp1 traf4a npl prkci urod sh3bp5 lamp2 rhag atp6v0c gfap gclc zgc:77744 fgf13l zgc:63700 hif1ab flt4 ctsc zgc:55307 anp32b zgc:77517 limk2 zgc:77775 atp6v0a1 bhlhe40 vasn pigq znf703 sox7 psap rap1b fli1b ppp1r14b si:ch211-217g15.2 mpx jak1 fos dnmt4 sult2st1 pak2b sgk1 dfna5 slc4a2a arl4l cebpa hapln3 her4.1 zgc:64022 epas1 prox1 mmp9 hmbsb fzd2 entpd1 pnrc2 pglyrp5 mybl1 gng2 gtpbp1 fgfrl1b itm2bb si:dkey-146n1.1 zgc:64051 hey1 cpne1 setd2 si:ch211-212m21.5 hspa12b pfkfb4l si:dkey-220f10.6 uros ahr2 |
| 26 | miR-223 | adam8a ncor2 cnn3a heg elovl5 prdx5 slc12a3 slc20a1a abce1 prkci bcl2l calm1a sh3bp5 zgc:91860 lamp2 ftr82 klfd rhag dnajb11 gfap gclc snx5 flt4 vegfab limk2 sept9a tbx6 cdh17 pigq bmpr2a cx43 bactin1 sox7 fli1b etv2 nat13 tagln2 spi1 lgmn plagx cav1 lgals9l1 lgals2b nccrp1 dfna5 arl4l alas2 cebpa gata2a ca10a ilk birc5b suv39h1b krt8 hmbsb plag1 her4.1 cybb zgc:112062 epas1 prox1 appb ak3 elovl1b tal1 entpd1 mybl1 dhrs3b fgfrl1b mpp1 sh3gl1b slc25a37 limk1 glula clgn uros grnb pglyrp2 hcst akap12 gygl plagl2 |
| 27 | miR-24 | heg atp1b1a sh3gl3 traf4a lamp2 klfd rhag baxa zgc:66441 ptpn6 krt18 ctsc zgc:55307 tbx6 vasn calm1a rap1b fzd7b fli1b txnip mpx nat13 tagln2 pls1 lgmn fli1a cndp2 kpnb3 cnn2 prkcd kdr calr dub si:dkey-222f8.3 seph cxcr4a vsg1 selt1a fgfrl1b mpp1 si:dkey-146n1.1 cpox zgc:66433 uros pfn2 slc43a1a ahr2 plagl2 zgc:55891 rasgrp3 |
| 28 | miR-27b | slc8a1b znfl2a jak2a heg nt5c2l1 atp1b1a elovl5 kdrl slc12a3 slc20a1a abce1 traf4a prkci gats gata1 arrdc2 atp2b1a lamp2 hspa5 cpn1 klfd zgc:73136 dnajb11 baxa nop58 ctsl1a dlc gfap gata3 fgf13l snx5 mcam csrp1 osr1 vegfab limk2 slc10a4 atp6v0a1 bhlhe40 vasn si:dkey-78p20.2 pitpna brd2a pigq rab5c cdx4 cx43 agfg1a itgav krcp fzd7b fli1b ppp1r14b mpx copb2 tagln2 aplnrb fos pls1 spi1 plagx rhoua cav1 dnmt4 cyp1a lgals9l1 cbfb dnajc3 prdx2 ncf1 arl4l lmo4 ilk plag1 seph micall2 ctsz crlf3 freqa appb ak3 tal1 vsg1 zbtb2b lpar1 entpd1 fhl pnrc2 zgc:110219 gcm2 fgfrl1b pak2a mpp1 itm2bb lgals3bpb hey1 cpne1 slc25a37 cpox drl si:ch211-212m21.5 pfkfb4l clgn si:dkey-220f10.6 uros pglyrp2 akap12 slc43a1a ahr2 bcl2 prim1 gygl birc5a cebp1 |
| 29 | miR-451 | slc8a1b adam8a heg map1lc3b nitr9 sh3gl3 slmo2 itgb3b mmp13 ipo9 slc12a3 traf4a npl gats hspa9 cahz atp2b1a zgc:91860 dhfr lamp2 tln1 ap2m1a ctsl1a dlc shbg snx5 mmp2 zgc:63700 krt18 hif1ab atp6v1d glud1a dab2 dera dusp5 rh30 arl4a si:dkey-78p20.2 mcm3 zgc:110010 cdx4 bactin1 agfg1a lmo2 fzd7b ppp1r14b txnip nat13 fli1a twist1a cndp2 kpnb3 cav1 smad5 lgals9l1 ncf1 gpr182 arpc1b adka slc4a2a pgm3 cebpa prcp ilk suv39h1b plag1 calr hapln3 zgc:64022 znf148 si:dkey-222f8.3 prox1 seph htt crlf3 fstl1b nrp2a tal1 bmp4 aspn cxcr4a sla1 zbtb2b selt1a fhl mybl1 rab14 myct1 zgc:110219 fgfrl1b mafbb pdlim2 zgc:63734 inka1b hey1 cpne1 slc25a37 dll4 alp si:ch211-212m21.5 zgc:66433 si:dkey-220f10.6 akap12 ahr2 gygl |
