# Supplementary material for: Reverse Genetics Screen in Zebrafish Identifies a Role of miR-142a-3p in Vascular Development and Integrity
Source: PLoS One. 2012 Dec 21;7(12):e52588. doi: 10.1371/journal.pone.0052588 (PMC3528674; doi:10.1371/journal.pone.0052588)
Supplement: Table S5 — The list of oligos sequence used in the study (DOC) [file pone.0052588.s010.doc]

**Supplementary Table S5**: The list of oligos sequence used in the study.

| **S.no.** | **Oligo Name** | **Sequence** |
| --- | --- | --- |
| 1 | miR-142a-3p sense | UGUAGUGUUUCCUACUUUAUGGAdTdT |
| 2 | miR-142a-3p anti- sense | UCCAUAAAGUAGGAAACACUAGAdTdT |
| 3 | miR-144 sense | UACAGUAUAGAUGAUGUACUdTdT |
| 4 | miR-144 anti- sense | AGUACAUCAUCUAUACUGAAdTdT |
| 5 | miR-451 sense | AAACCGUUACCAUUACUGAGUUdTdT |
| 6 | miR-451 anti- sense | AACUCAGUAAUGGUAACGGUAUdTdT |
| 7 | miR-221 sense | AGCUACAUUGUCUGCUGGGUUUCdTdT |
| 8 | miR-221 anti- sense | GAAACCCAGCAGACAAUGUAGGUdTdT |
| 9 | miR-222 sense | AGCUACAUCUGGCUACUGGGUCUCdTdT |
| 10 | miR-222 anti- sense | GAGACCCAGUAGCCAGAUGUAGGUdTdT |
| 11 | miR-181a sense | AACAUUCAACGCUGUCGGUGAGUdTdT |
| 12 | miR-181a anti- sense | ACUCACCGACAGCGUUGAAUGAUdTdT |
| 13 | miR-181b sense | AACAUUCAUUGCUGUCGGUGGGdTdT |
| 14 | miR-181b anti- sense | CCCACCGACAGCAAUGAAUGAUdTdT |
| 15 | miR-1 sense | UGGAAUGUAAAGAAGUAUGUAUdTdT |
| 16 | miR-1 anti- sense | AUACAUACUUCUUUACAUUCGAdTdT |
| 17 | 3’ UTR Cdh5 forward primer (p1361) | AAACTCGAGCCTCCATGCATTGTTATAATG |
| 18 | 3’ UTR Cdh5 reverse primer (p1362) | AAATCTAGAATCAATCATAAGCACGTGCCA |
| 19 | miR-142a-3p PT Forward oligo p1196 | AAACTCGAGATCCATAAAGTAGGAAACACTACATAGCTAATCCATAAAGTAGGAAACACTACATAGCTAATCCATAAAGTAGGAAACACTACA |
| 20 | miR-142a-3p PT reverse oligo p1197 | AAATCTAGAATGTAGTGTTTCCTACTTTATGGATTAGCTATGTAGTGTTTCCTACTTTATGGATTAGCTATGTAGTGTTTCCTACTTTATGGA |
| 21 | 3X site B Forward oligo | AAACTCGAGCCTCCATGCATTGTTATAATGAACACTACTTAGCTAACCTCCATGCATTGTTATAATGAACACTACTTAGCTAACCTCCATGCATTGTTATAATGAACACTACT |
| 22 | 3X site B reverse oligo | AAATCTAGAAGTAGTGTTCATTATAACAATGCATGGAGGTTAGCTAAGTAGTGTTCATTATAACAATGCATGGAGGTTAGCTAAGTAGTGTTCATTATAACAATGCATGGAGG |
| 23 | 3X site A Forward oligo | AAACTCGAGCTACGCACAGTTAAAAGATTTAGCACTACAGTAGCTAACTACGCACAGTTAAAAGATTTAGCACTACAGTAGCTAACTACGCACAGTTAAAAGATTTAGCACTACAG |
| 24 | 3X site A reverse oligo | AAATCTAGACTGTAGTGCTAAATCTTTTAACTGTGCGTAGTTAGCTACTGTAGTGCTAAATCTTTTAACTGTGCGTAGTTAGCTACTGTAGTGCTAAATCTTTTAACTGTGCGTAG |
| 25 | 3X mut site B Forward oligo | AAACTCGAGCCTCCATGCATTGTTATAATGATGTCAAGATAGCTAACCTCCATGCATTGTTATAATGATGTCAAGATAGCTAACCTCCATGCATTGTTATAATGATGTCAAGA |
| 26 | 3X mut site B reverse oligo | AAATCTAGATCTTGACATCATTATAACAATGCATGGAGGTTAGCTATCTTGACATCATTATAACAATGCATGGAGGTTAGCTATCTTGACATCATTATAACAATGCATGGAGG |
| 27 | 3X mut site A Forward oligo | AAACTCGAGCTACGCACAGTTAAAAGATTTAGGTCAAGTGTAGCTAACTACGCACAGTTAAAAGATTTAGGTCAAGTGTAGCTAACTACGCACAGTTAAAAGATTTAGGTCAAGTG |
| 28 | 3X mut site A reverse oligo | AAATCTAGACACTTGACCTAAATCTTTTAACTGTGCGTAGTTAGCTACACTTGACCTAAATCTTTTAACTGTGCGTAGTTAGCTACACTTGACCTAAATCTTTTAACTGTGCGTAG |
| 29 | RT-PCR Cdh5 forward primer(p1457) | TTCGGAGGAATATGTGCTGG |
| 30 | RT-PCR Cdh5 reverse primer(p1458) | GATACAGAGAAGGATGGCGA |
| 31 | miR-142a-3p MO | TCCATAAAGTAGGAAACACTACA |
| 32 | 3’ UTR vegfab forward primer (p1371) | AAACTCGAGGGTCTGCTGTGCAGTTTGTTT |
| 33 | 3’ UTR vegfab reverse primer (p1372) | AAATCTAGACTAAAATTCCAAGACGAGCTG |
| 34 | Cdh5_F in situ (p1719) | CTGTCATTGGAGCTGTTTCAGCA |
| 35 | Cdh5_F in situ (p1720) | CTTTCAAAAAGCGGTAGCATGGG |
| 36 | Lmo2 MO_ATG_1 | GTAGAAGCCATTTTCAATATGATTC |
| 37 | Lmo2 MO_ATG_2 | CAGTGGCGTTGTTCAATTTCTCCGG |
